# Expanding the chemical functionality of DNA nanomaterials generated by rolling circle amplification

**DOI:** 10.1093/nar/gkab720

**Published:** 2021-08-17

**Authors:** Ysobel R Baker, Liyiwen Yuan, Jinfeng Chen, Roman Belle, Robert Carlisle, Afaf H El-Sagheer, Tom Brown

**Affiliations:** Chemistry Research Laboratory, University of Oxford, 12 Mansfield Road, Oxford OX1 3TA, UK; Chemistry Research Laboratory, University of Oxford, 12 Mansfield Road, Oxford OX1 3TA, UK; Chemistry Research Laboratory, University of Oxford, 12 Mansfield Road, Oxford OX1 3TA, UK; Chemistry Research Laboratory, University of Oxford, 12 Mansfield Road, Oxford OX1 3TA, UK; Institute of Biomedical Engineering, University of Oxford, Oxford, OX3 7DQ, UK; Chemistry Research Laboratory, University of Oxford, 12 Mansfield Road, Oxford OX1 3TA, UK; Chemistry Branch Department of Science and Mathematics, Suez University, Suez 43721, Egypt; Chemistry Research Laboratory, University of Oxford, 12 Mansfield Road, Oxford OX1 3TA, UK

## Abstract

Rolling circle amplification (RCA) is a powerful tool for the construction of DNA nanomaterials such as hydrogels, high-performance scaffolds and DNA nanoflowers (DNFs), hybrid materials formed of DNA and magnesium pyrophosphate. Such DNA nanomaterials have great potential in therapeutics, imaging, protein immobilisation, and drug delivery, yet limited chemistry is available to expand their functionality. Here, we present orthogonal strategies to produce densely modified RCA products and DNFs. We provide methods to selectively modify the DNA component and/or the protein cargo of these materials, thereby greatly expanding the range of chemical functionalities available to these systems. We have used our methodology to construct DNFs bearing multiple surface aptamers and peptides capable of binding to cancer cells that overexpress the HER2 oncobiomarker, demonstrating their potential for diagnostic and therapeutic applications.

## Graphical Abstract

**Graphical Abstract:**
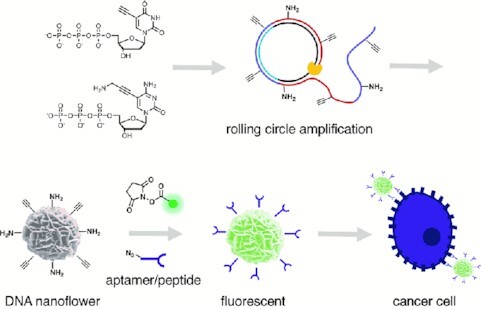
Combining modified dNTPs with rolling circle amplification to expand the functionality of DNA nanomaterials.

## INTRODUCTION

DNA provides an excellent basis for the fabrication of novel nanomaterials (1), having many advantageous properties including biocompatibility, water solubility, structural programmability through Watson–Crick base pairing, and complementary target recognition. DNA-based nanomaterials are expected to have a major impact in the biomedical field, particularly in therapeutics and diagnostics, due to their high target specificity, extended half-life, and efficient eukaryotic cell uptake, the latter in contrast to free oligonucleotides, which do not enter cells efficiently (2,3).

Depending on the desired properties of the DNA nanomaterials, the DNA component can be synthetic (4,5), isolated from biological sources (e.g. bacteriophages) (6), or produced using amplification techniques such as the polymerase chain reaction (PCR) (7,8) or rolling circle amplification (RCA) (9). These alternative approaches produce DNA on varying length scales and with different characteristics. Uniquely, RCA produces long concatemers of single stranded DNA (ssDNA) that can be assembled into a variety of nanomaterials (10) including nanoribbons (11), nanosprings (12), and templating nanoscaffolds (13).

**Figure 1.**
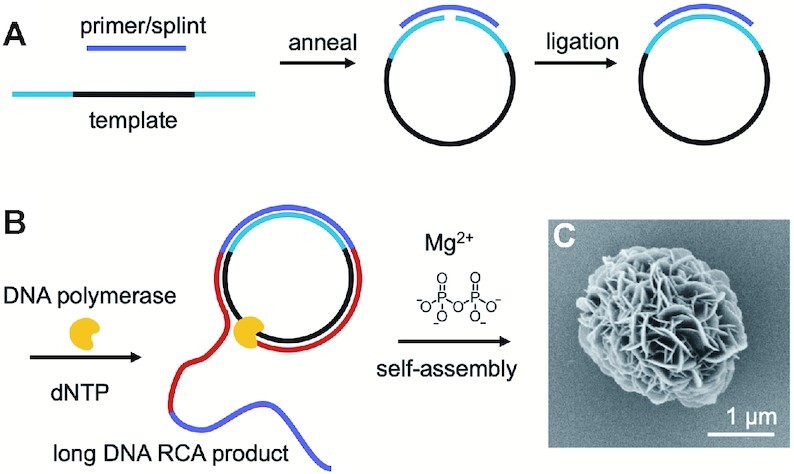
Enzymatic preparation of DNFs. (**A**) The linear DNA template with a 5′ phosphate is annealed to a DNA splint and cyclised using T4 DNA ligase. (**B**) The splint is extended using a DNA polymerase with strand displacement activity (yellow) to give long ssDNA formed of repeating units (red and purple). The DNA then co-crystallises with the pyrophosphate by-product and the Mg^2+^ ions present in the amplification buffer to give DNFs. (**C**) Scanning electron microscopy (SEM) image of a DNF prepared using unmodified dNTPs.

RCA can also be used to synthesise DNA nanoflowers (DNFs), which are DNA-inorganic hybrid superstructures with flower-like morphology (Figure 1) (14). During RCA, a DNA polymerase, typically the highly processive strand-displacement polymerase phi29, traverses a circular DNA template very many times, producing long single strands of DNA of repeating sequence, which are complementary to the template. A pyrophosphate anion (PPi) is released with each dNTP incorporation, and these poly-anions co-crystallise with both the Mg^2+^ cations present in the amplification buffer and the growing DNA chains to form hybrid nanostructures (15). Analogous to this, rolling circle transcription can be used to prepare materials termed RNA microsponges, consisting of RNA, PPi and Mg^2+^ ions (16). DNFs benefit from the favourable biological properties and programmability of DNA combined with the structure and stability associated with Mg_2_P_2_O_7_ complexation.

DNFs have promise as therapeutic and diagnostic agents owing to their enhanced cellular uptake, potential cell targeting ability, and resistance to nuclease degradation (14,17). Through design of the circular template, DNFs can be programmed to contain specific functionalities such as aptamers for cell targeting (14), immuno-stimulatory regions (18), DNAzymes for targeted RNA degradation (19), and drug loading sequences (17). The size of the particles can be controlled to a degree by altering the amplification time (17), adjusting the Mg^2+^ concentration (20), or by condensing the resulting particles (21). DNFs can be prepared that contain proteins, including enzymes, for intracellular delivery and catalytic enhancement (22), and the inorganic core of the particles can be altered by changing the reaction buffer (23,24). Despite these advances, there is limited chemistry available to modify the DNA component of these particles, or to attach extra functionality to the nanoconstructs.

Here, we present a simple 2-step approach that utilises modified dNTPs with small chemical handles which are added at high concentration during the construction of the DNFs. Many materials generated by RCA rely on Watson–Crick base pairing for function and structure, hence we decided that the non-hydrogen bonding faces of the nucleobases would be the optimum positions to attach functionality. Avoiding blocking the base pairing hydrogen-bonding faces of the nucleobases is also important in achieving efficient and accurate template copying during RCA. We demonstrate the effectiveness of this approach in DNF synthesis, and show that the choice of modified dNTP and labelling conditions significantly affect the outcome of RCA as well as the nature of the materials produced. Our methodology can be used to attach a range of chemistries to the surface of the DNFs, and to modify the protein cargo of DNFs, leaving the DNA component intact. The concept of functionalising protein cargo encapsulated inside DNFs has great potential as it provides additional orthogonality for DNF functionalisation.

To evaluate the clinical potential of our DNF modification methodology we have utilised a system of diagnostic and therapeutic relevance, the human epidermal growth factor receptor (HER2) which is over-expressed in >20% of breast cancers. We have investigated chemically modified DNFs capable of enhanced binding to HER2^+^ cancer cells, hypothesising that functionalising the surface of a DNF with a HER2 targeting aptamer (25) might improve its interaction with HER2^+^ cells due to cooperativity. We also performed a similar study using a HER2-recognition peptide and achieved enhanced cell binding.

## MATERIALS AND METHODS

General protocols for the synthesis of oligonucleotides, cyclisation of templates, and analytical techniques along with the sequences of oligonucleotides (Supplementary Table S1) and peptides (Supplementary Table S2) are given in the Supplementary Information.

### General RCA procedure

A 10 μl solution containing cyclised template (6 pmol), splint (12 pmol), 2 μl of 10× NexGen phi29 reaction buffer (500 mM Tris–HCl, 100 mM (NH_4_)_2_SO_4_, 40 mM dithiothreitol (DTT), 100 mM MgCl_2_, pH 7.5 @ 25°C) and 1 μl of 100 mM MgSO_4_ in MilliQ water in a 200 μl PCR tube was heated to 95°C for 5 min before cooling to 25°C at a rate of 0.5°C/min. To this solution was added the dNTP mix in MilliQ water (9 μl, see dNTP concentrations below) followed by phi29 (1 μl, 10 000 U/ml). The reaction was incubated in a thermocycler at 30°C for 20 h (unless otherwise stated), heated to 65°C for 10 min, and cooled to room temperature.

### Preparation of functionalised DNFs

Modified DNFs were synthesised following the general RCA procedure described above. The dNTP mix was prepared using 100 mM stock solutions of dATP, dTTP, dGTP, dCTP and modified dNTP, and the total volume was adjusted to 9 μl with MilliQ water. Example: the dNTP mixture for a 50% ethynyl-dUTP **2** DNF would consist of 0.4 μl of 100 mM dATP, 0.4 μl of 100 mM dGTP, 0.4 μl of 100 mM dCTP, 0.2 μl of 100 mM dTTP, 0.2 μl of 100 mM ethynyl-dUTP **2**, and 7.4 μl of water. To avoid errors associated with pipetting small volumes, dNTP mixes were prepared in larger volumes and 9 μl was taken from this.

After the RCA step, the samples were diluted with water (80 μl) and transferred to a larger 1.5 ml Eppendorf tube. The PCR tube was washed with 200 μl of water, which was added to the Eppendorf tube. This was repeated twice. The DNFs were then collected by centrifugation and the supernatant was discarded. The resulting pellet was resuspended in MilliQ water (0.75 ml) and collected by centrifugation. The process was repeated three times.

### Strain promoted alkyne-azide cycloaddition (SPAAC) labelling of azide-DNFs

DNFs were prepared using the appropriate azide-functionalised-dUTP. The DNFs were then suspended in 20 μl of labelling buffer (10 mM HEPES, pH 8.0, 10 mM MgSO_4_, 50% v/v DMSO) containing 1 mM of a bicyclo[6.1.0]nonyne (BCN) or a dibenzocyclooctyne (DBCO) functionalised moiety and gently agitated at room temperature for 2 h. The DNFs were then collected by centrifugation and the supernatant discarded. The resulting pellet was resuspended in 100 μl of labelling buffer and collected by centrifugation. The process was repeated twice with 50% (v/v) DMSO in MilliQ water and twice with MilliQ water to ensure all salts and small molecules were removed.

### Copper catalysed alkyne-azide cycloaddition (CuAAC) labelling of azide and alkyne DNFs

DNFs were prepared using the appropriate dUTPs. The DNFs were then suspended in 20 μl of labelling buffer (10 mM HEPES, pH 8.0, 10 mM MgSO_4_, 50% v/v DMSO) containing 1 mM alkyne or azide, 0.5 mM CuSO_4_, 0.5 mM tris-hydroxypropyltriazolylmethylamine (THPTA) and 2.5 mM sodium ascorbate (NaAsc) and gently agitated at room temperature for 2 h. The DNFs were then collected by centrifugation and the supernatant was discarded. The resulting pellet was resuspended in 100 μl of labelling buffer and collected by centrifugation. The process was repeated twice with 50% (v/v) DMSO in MilliQ water and twice with MilliQ water to ensure all salts and small molecules were removed.

### *N*-Hydroxysuccinimide (NHS) ester labelling of amino-DNFs

DNFs were prepared using the appropriate amino dUTPs, which were suspended in 20 μl of labelling buffer (10 mM HEPES, pH 8.0, 10 mM MgSO_4_, 50% v/v DMSO) containing 1 mM of the NHS active ester. The mixture was incubated at room temperature for 4 h with gentle agitation. The DNFs were then collected by centrifugation and the supernatant was discarded. The resulting pellet was resuspended in 100 μl of labelling buffer and collected by centrifugation. The process was repeated twice with 50% (v/v) DMSO in MilliQ water and twice with MilliQ water to ensure all salts and small molecules were removed.

### Dual labelling of DNFs using CuAAC and NHS active esters

DNFs were prepared using the appropriate dUTPs. They were then suspended in 20 μl of labelling buffer (10 mM HEPES, pH 8.0, 10 mM MgSO_4_, 50% v/v DMSO) containing 1 mM alkyne or azide, 1 mM of the NHS active ester, 0.5 mM CuSO_4_, 0.5 mM THPTA and 2.5 mM NaAsc, and this was incubated at room temperature for 2 h with gentle agitation. The DNFs were then collected by centrifugation and the supernatant was discarded. The resulting pellet was resuspended in 100 μl of labelling buffer and collected by centrifugation. The process was repeated twice with 50% (v/v) DMSO in MilliQ water and twice with MilliQ water to ensure all salts and small molecules were removed.

### Dual labelling of DNFs using SPAAC and NHS active ester labels

DNFs were prepared using the appropriate modified dNTPs. The DNFs were then suspended in 20 μl of labelling buffer (10 mM HEPES, pH 8.0, 10 mM MgSO_4_, 50% v/v DMSO) containing 2.5 mM of the BCN or DBCO functionalised label and 1 mM of the NHS active ester and this was incubated at room temperature for 2 h with gentle agitation. The DNFs were then collected by centrifugation and the supernatant was discarded. The resulting pellet was resuspended in 100 μl of labelling buffer and collected by centrifugation. The process was repeated twice with 50% (v/v) DMSO in MilliQ water and twice with MilliQ water to ensure all salts and small molecules were removed.

## RESULTS AND DISCUSSION

### Overview of the strategy used to generate chemically modified RCA nanomaterials

**Figure 2.**
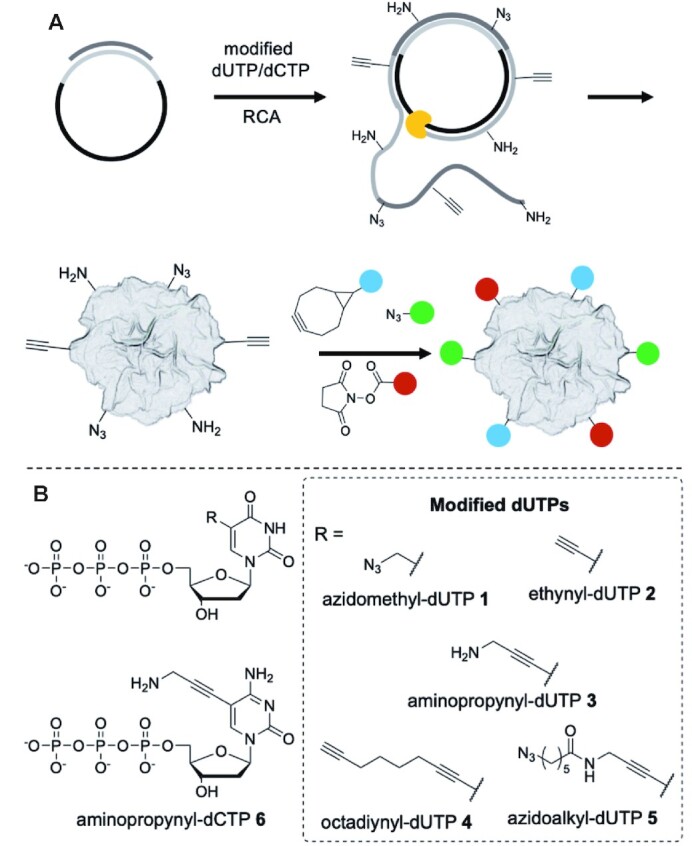
(**A**) Enzymatic preparation of amine, alkyne and azide-functionalised DNFs and subsequent orthogonal labelling. (**B**) Modified dNTPs investigated in this study.

We have used modified dNTPs in RCA to generate long ssDNA with azide, alkyne and/or amine handles (Figure 2). The ssDNA co-crystallises with PPi and Mg^2+^ to give DNFs with chemical handles that can be used for post-synthetic functionalisation, either through CuAAC (26,27), SPAAC (28) using a cyclooctyne such as BCN (29), or NHS ester mediated amine labelling. The five modified dNTPs **1–5** are derivatives of dTTP, whereas **6** is a derivative of dCTP. By altering the ratio of modified to unmodified dNTPs it is possible to control the degree of labelling, and using separate dCTP and dTTP analogues with different chemical handles enables high levels of dual labelling within the same DNA construct. We prepared dNTPs **1** (30), **3** (31) and **5** (32) according to literature procedures and converted these to their sodium salts before use. The dNTPs **1**–**4** and **6** are commercially available and aminopropynyl-dUTP **3** has been used with phi29 previously (33). The sequences and cyclisation protocols of the oligonucleotides used in this study are presented in the supplementary information.

### The nature of the side-chain on the modified dNTPs alters the quantity of DNA produced during DNF synthesis

To achieve a high level of DNF functionality we needed to identify modified dNTPs that could be incorporated into DNA at high density without greatly disrupting the quantity of DNA that is produced and incorporated into the DNFs. Whilst modified dNTPs have been used previously to add functionality to RCA products, phi29 does not incorporate bulky modified dNTPs without a reduction in polymerase efficiency (33–37). Hence, a standard approach in RCA is to introduce modified dNTPs at low concentration in the reaction mixture (typically 40 μM) in the presence of a much higher concentration of the four canonical A, G, C, T triphosphates (typically 2 mM of each) (38). This approach obviously gives very low labelling density, so we investigated much higher concentrations of modified dNTPs in an attempt to increase the level of functionalisation.

We first examined the effect of modified dNTPs **1**–**6** (Figure 2B) on the DNA produced using agarose gel electrophoresis analysis (Supplementary Figures S1–S6). In these experiments we used a previously reported DNF 83-mer template (39) (Supplementary Table S1, template 1) and carried out DNF synthesis substituting the unmodified dNTP with varying percentages of corresponding modified dNTP whilst maintaining a total dNTP concentration of 2 mM. We kept the total reaction volumes and concentrations of all other components the same to allow direct comparisons. Following RCA, we separated the DNA product into DNA that assembled into precipitate, which we assume contained DNFs, and ‘free’ DNA that remained in solution. At this stage we did not differentiate between amorphous precipitate and structured DNFs, but later we examined this in detail. We isolated the insoluble DNA from the RCA mixture by centrifugation, and visualised the RCA products using agarose gel electrophoresis. All modified dNTPs tested gave what we assumed to be very long extended DNA, as visualised in the wells of the gel, even with complete dNTP substitution. However, we cannot be certain that the material in the wells was not short aggregated DNA. In some cases, discrete bands with higher gel mobility were observed, but the yields of this band varied significantly. The discrete faster moving bands were more apparent in the ‘free’ DNA and we hypothesise that they are too short for efficient incorporation into DNFs.

**Figure 3.**
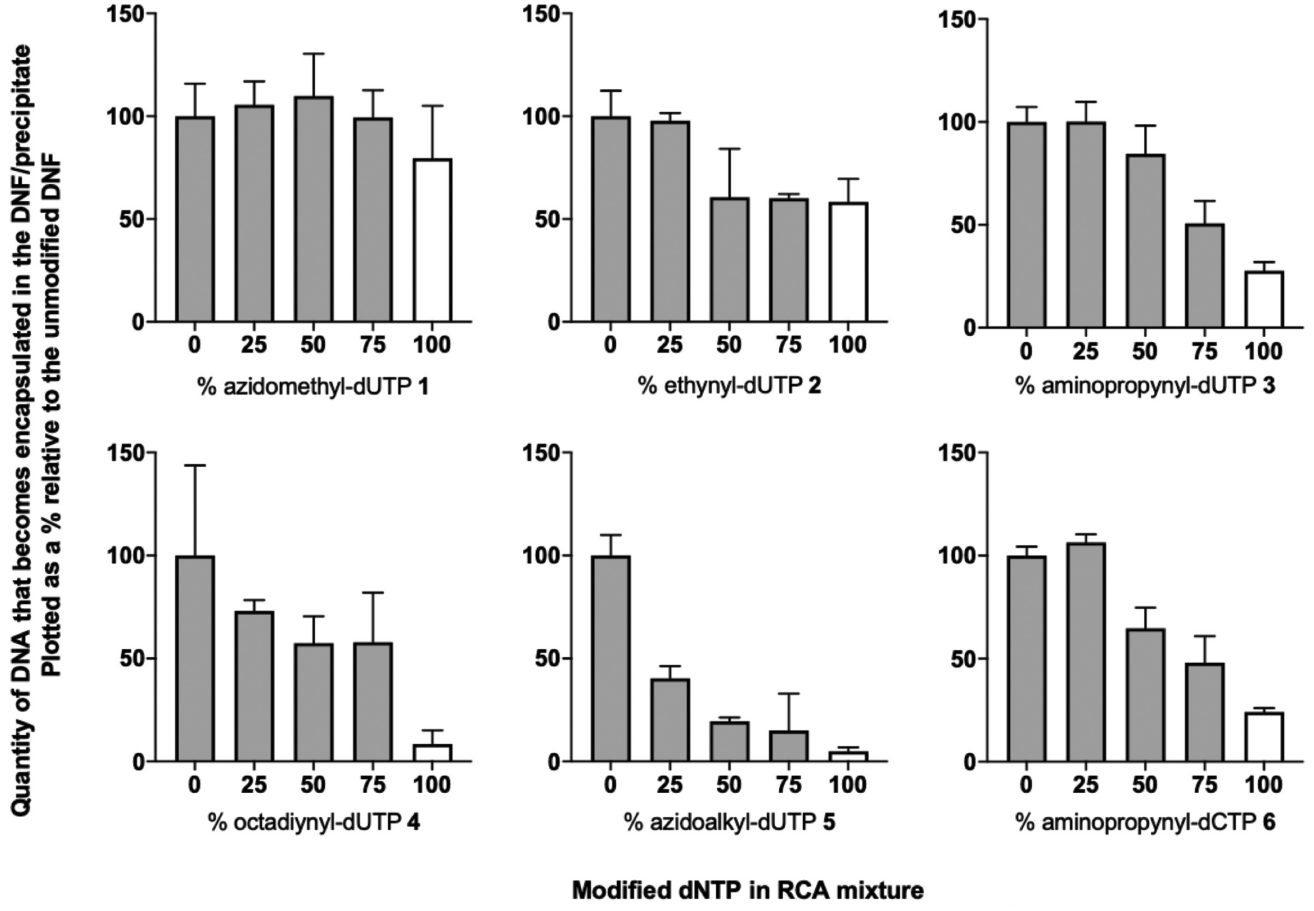
SYBR gold fluorescence-based comparison of the relative amounts of DNA that becomes encapsulated in the DNFs/precipitate during phi29-catalysed RCA when dTTP or dCTP were substituted with different percentages of each of the modified dNTPs in the amplification step. In all cases the total dNTP concentration was kept at 2 mM and the percentage of modified dNTP is indicated on the x-axis. Tris-borate-ethylenediaminetetraacetic acid (TBE) was added prior to incubation with SYBR gold to break down the DNFs and release the entrapped DNA. SEM images before and after TBE treatment are given in the supplementary information (Supplementary Figure S7). Error bars represent the standard deviation of three different RCA reactions, and two separate independent data sets were combined. The sequence of the template and splint used are given in the SI (Supplementary Table S1, template 1, splint 1). Data is plotted as a % relative to unmodified RCA reactions.

The bulk of the precipitated DNA was too long to migrate from the wells of the gel. Hence only limited information was obtained on the effect of the modified dNTPs on the yield of DNFs/precipitated DNA. To investigate this further we compared the amount of DNA incorporated into the DNFs or precipitate by measuring total fluorescence after staining with SYBR gold, a fluorescent dye that binds both single and double stranded DNA (Figure 3) (23). Complete substitution of dUTP with azidomethyl-dUTP **1** resulted in only 10% reduction in the DNA incorporated into the DNFs/precipitate. In contrast, full substitution of dUTP with the larger azidoalkyl-dUTP **5** led to a 95% reduction in the amount of DNA present in the precipitate. This is the least useful of all the modified dNTPs analysed as it appears to reduce the amount of DNA present in the precipitate at just 25% substitution. We hypothesise that the incorporation of azidoalkyl-dUTP **5** results in a particularly hydrophobic RCA product, which precipitates or aggregates, preventing further extension of the DNA chain. We observed a similar size-related trend when comparing ethynyl-dUTP **2** and the bulkier octadiynyl-dUTP **4**, suggesting that phi29 tolerates small nucleobase modifications better than larger ones. Efficient incorporation of the short side-chain aminopropynyl-dUTP **3** and aminopropynyl-dCTP **6** was observed, with only a 50% reduction in total precipitated DNA, even at 75% substitution. These results agree with other reports not related to DNFs (33–37).

At 100% substitution there is an inverse correlation between the size (and flexibility) of the side-chain of the pyrimidine base and the amount DNF/precipitate that forms i.e. yield of precipitate/DNF formation = unmodified > azidomethyl > ethynyl > aminopropynyl > octadiynyl > azidoalkyl (compare white bars in Figure 3). The flexibility of azidomethyl leads to it performing slightly better than the rigid ethynyl side chain, perhaps because the former is more readily accommodated in the polymerase active site. The data at 100% substitution is particularly important as we can be sure that the modified dNTP is actually being incorporated into the precipitate/DNFs. At all other percentages of modified base the unmodified dNTP might be selectively utilised by the polymerase, leading to lower than predicted incorporation of the modification.

To summarise, the sterically undemanding ethynyl, azidomethyl and aminopropynyl-dNTPs all performed well and gave high levels of functionalised DNA both in solution and importantly in the precipitated products.

### The choice of modified dNTPs has a significant effect on the structure and size of DNFs

**Figure 4.**
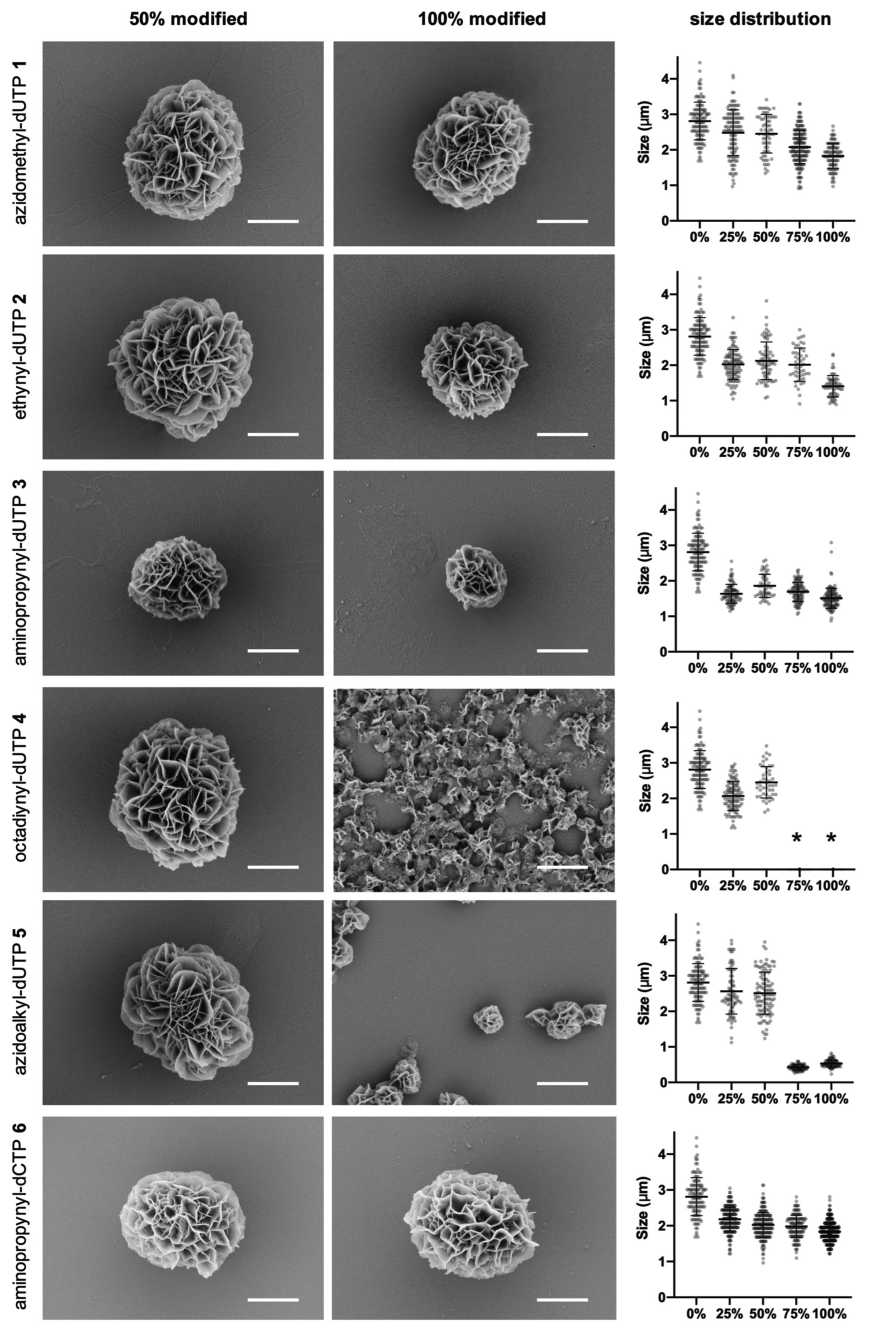
SEM images and particle size distribution of DNFs produced with the different modified dNTPs as a function of percentage modified dNTP used. For SEM, we prepared DNFs using 1:1 modified dNTP to corresponding unmodified dNTP (50% modified) or with complete replacement of the unmodified dNTP (100% modified). Scale bars represent 1 μm. Figure 1C shows a typical unmodified DNF. Further images (low magnification, 25% and 75% modified) are shown in the supplementary information (Supplementary Figures S8–S14). The size distributions are plotted against % modified dNTP along the x-axis. Images used for the size distribution measurements are given in the supplementary information (Supplementary Figures S15–S37). * indicates no distinct DNF structures.

The above experiments do not allow us to differentiate between DNA incorporated into DNFs and DNA precipitated as an amorphous solid. Hence it was important to carry out imaging studies to examine morphology. For this we used Scanning Electron Microscopy (SEM), which also allowed us to compare the impact of the nucleotide modifications on DNF size (Figure 4, and Figures S8–S14). At 100% substitution, the least bulky azidomethyl and ethynyl dU triphosphates **1** and **2** had a minimal effect on structure, but they slightly reduced the size of the DNFs. In contrast, the bulky hydrophobic octadiynyl dU modification **4** prevented the formation of full DNFs, and a mesh like precipitate was observed. Long chain azido-dU modification **5** resulted in very small particles, presumably due to the significant reduction in DNA that precipitated during the RCA (Figure 3). It is noteworthy that modified dNTPs **4** and **5** both have long hydrophobic chains attached to the nucleobase which are more likely to affect the physical properties of the DNFs, as well as affecting the ability of the modified DNA products to self-assemble.

In general, the sizes of particles formed were inversely proportional to the percentage of the modified triphosphate used. This is probably a consequence of the reduced DNA yields (Figure 3). Both the aminopropynyl modified dNTPs **3** and **6** gave a marked decrease in DNF size at 25% substitution, and the size was consistent with increasing percentages of modified dNTP. This could be due to interactions of the cationic amine with the anionic phosphodiesters in the DNA (37) or with the pyrophosphate component of the DNFs, leading to condensed structures. As the SEM imaging techniques used were not quantitative, the images presented cannot be used to draw conclusions on numbers of DNFs produced; however, it is satisfying that all the modified dNTPs produced structured DNFs at very high (50%) substitution.

The results shown in Figures 3 and 4 highlight the importance of understanding how dNTP modifications can alter the properties of DNA nanostructures generated during RCA. In particular, the nature of the modified dNTPs clearly enables control of the size of the resulting DNFs. This is important, as the size of a nanomaterial is one of the factors that determines its fate *in vivo* (40). Hence, this is a promising strategy to pursue when developing novel therapeutic agents.

### Functionalisation of RCA generated DNA nanomaterials using orthogonal labelling chemistry

Having established that we can use the modified dNTPs **1**, **2**, **3** and **6** at the highest level of substitution to prepare structured DNFs, we then investigated if the functional handles (azide, alkyne and amine) could then be reacted with chemical labels. We explored attachment of fluorescent dyes to the DNFs to provide a simple measurable property.

#### NHS ester labelling

**Figure 5.**
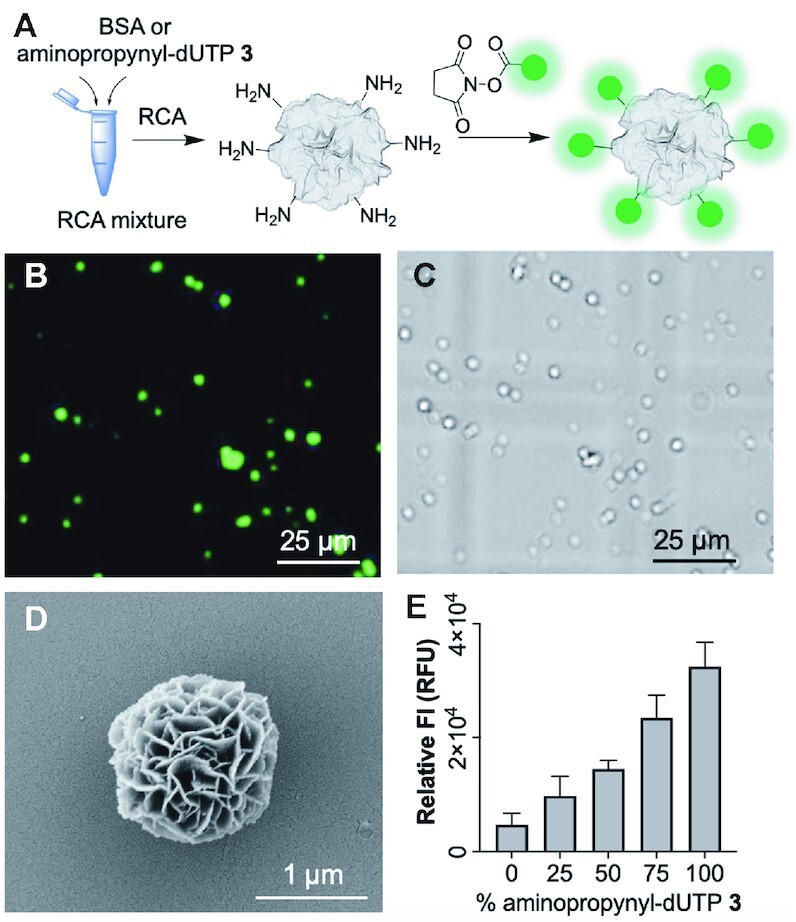
NHS ester labelling of H_2_N-DNFs. (**A**) Schematic of the method; (**B**) Fluorescence image of FAM functionalised H_2_N-DNFs under 475 nm excitation prepared using 50% aminopropynyl-dUTP **3**; (**C**) bright-field image of sample shown in (B). Some particle migration occurs as the samples are in water; (**D**) SEM image of FAM modified DNF prepared with 50% aminopropynyl-dUTP; (**E**) relative FAM fluorescence intensity (FI) of DNFs containing varying levels of aminopropynyl-dU labelled with FAM-NHS. Error bars represent the standard deviation of three repeat reactions. A ‘buffer only’ control was analysed, and the fluorescence value subtracted from each of the samples.

We first needed to identify a buffer for the functionalisation reactions that would not damage the DNFs by altering the DNF morphology or DNA content. Maintaining the pH between 7.2 and 9.0 is necessary for efficient NHS-ester labelling of amines, and as many organic labels have low aqueous solubility, we investigated DMF, DMSO and acetonitrile as organic co-solvents. We incubated DNFs (unmodified) in different buffers (PBS, 10 mM HEPES, and 10 mM Tris), and evaluated their structure by SEM imaging. After separating the buffer from the DNFs, we carried out agarose gel electrophoresis to determine if any DNA had escaped from the structures into the buffer. A suitable buffer for the labelling experiments proved to be 10 mM HEPES at pH 8 with 50% DMSO as the co-solvent at room temperature, and we included 10 mM Mg^2+^ to prevent the DNA from disassociating from the nanoflowers. In contrast, PBS led to increased loss of DNA from the DNFs (see Supplementary Figure S38). This is a crucial observation given that many DNF protocols utilise PBS for DNF storage and/or fluorescence assisted cell sorting (FACS) experiments (17). H_2_N-DNFs prepared using aminopropynyl-dUTP **3** were treated with fluorescein-NHS (FAM-NHS) (Figure 5) in the above labelling buffer.

Fluorescence microscopy showed punctate fluorescence in the relevant channel (Figure 5B and C) and SEM analysis revealed that no structural perturbation occurred (Figure 5D). Next, we prepared H_2_N-DNFs using different ratios of aminopropynyl-dUTP **3** to dTTP. We collected the DNFs by centrifugation, washed them with water, and labelled them with FAM-NHS. We then treated the particles with EDTA to release the DNA, measured the relative fluorescence and compared it to an untreated sample. Whilst an increase in labelling occurred in comparison with the unmodified DNFs, non-specific labelling also occurred (Figure 5E). A small amount of phi29 polymerase is incorporated in the DNFs during RCA (22) and we hypothesise that the background labelling is occurring on the lysine amino groups of the enzyme. To test this, we used sodium dodecyl sulfate polyacrylamide gel electrophoresis (SDS-PAGE) to separate out the components of unmodified DNFs which had been subjected to labelling conditions using sulfo-Cy-3 NHS ester (FAM-NHS caused aggregation of phi29) (Supplementary Figure S39). As controls, unmodified phi29 and phi29 labelled with sulfo-Cy-3 NHS ester using the same labelling conditions were included on the gel. We observed bands with similar migration for both the DNFs and the sulfo-Cy-3-NHS labelled phi29. These migrated slightly further than the unmodified phi29 which was in line with the 75 kD ladder band. We attribute the broadness and faster migration relative to unmodified phi29 to variations in the number of labels on individual proteins as well as replacement of the positive charge from the lysines with a negative charge from the sulfo groups. These results suggest that the non-specific labelling was not occurring on the DNA, but rather on the encapsulated phi29 protein (Supplementary Figure S39). Expanding on this hypothesis we added varying quantities of bovine serum albumin (BSA), a lysine-rich protein which is known to be encapsulated into DNFs (22), during the RCA reaction. We then successfully labelled the particles with sulfo-Cy-3-NHS and analysed the resulting DNFs (Supplementary Figure S40). The DNFs with BSA were visibly pink and SDS-PAGE revealed a corresponding fluorescent band for sulfo-Cy3 labelled BSA. This indicates that labelling of DNFs can be achieved via addition of a lysine rich protein (the low amount of phi29 used during RCA does not result in a suitably high level of labelling). This BSA incorporation strategy is useful for the preparation of functionalised DNFs where the DNA component remains unmodified.

#### SPAAC labelling

**Figure 6.**
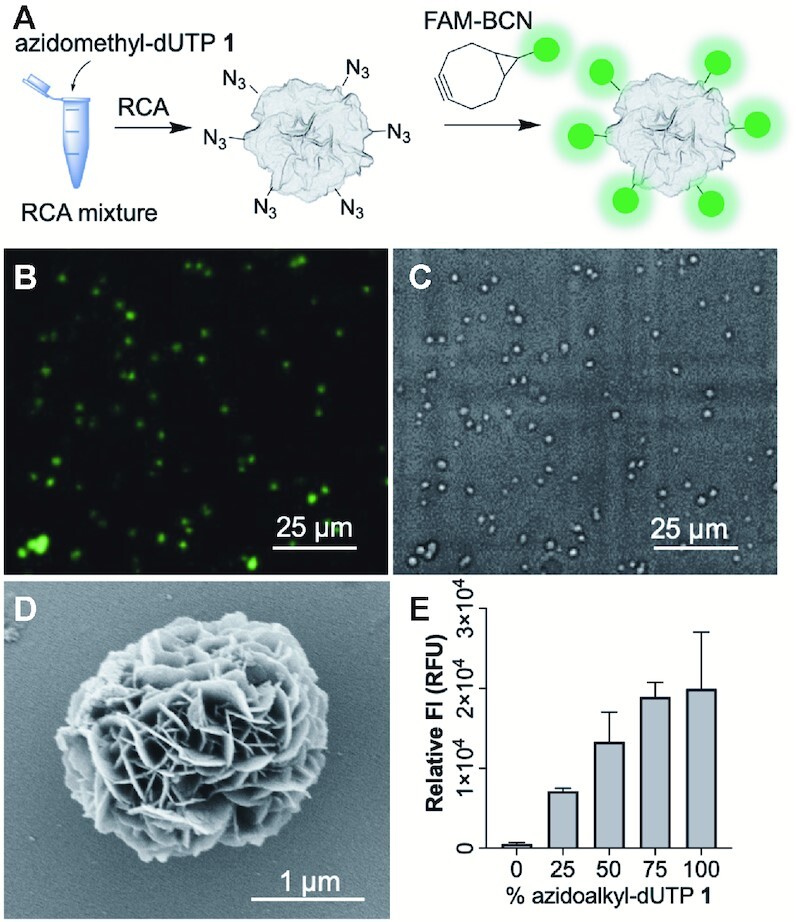
SPAAC labelling of N_3_-DNFs (azidomethyl-dU DNFs). (**A**) Schematic overview of methodology; (**B**) fluorescence image under 475 nm excitation of FAM functionalised N_3_-DNFs prepared using 50% azidomethyl-dUTP **1**; (**C**) bright-field image of sample shown in B. Some particle migration occurs as the samples are in water; (**D**) SEM images of FAM modified DNF prepared with 50% azidomethyl-dUTP **1**; (**E**) relative FAM fluorescence observed for DNFs containing varying levels of azidomethyl-dUTP **1** and labelled with FAM-BCN. Error bars represent the standard deviation of three repeat reactions. We also measured the fluorescence of a ‘buffer only’ control and subtracted the value from each of the samples.

Azide modified DNFs (N_3_-DNFs) were prepared using 50% azidomethyl-dUTP **1** and 50% dTTP during the RCA reaction. Initial labelling attempts using Cy5-BCN proved unsuccessful; we observed a fluorescent signal for both modified and unmodified DNFs treated under the same conditions, suggesting that the hydrophobic dye was sticking non-specifically to the particles (Supplementary Figure S41). To avoid this, we treated the N_3_-DNFs with the more polar fluorescein-BCN (FAM-BCN) in labelling buffer for 2 h (Figure 6A). The FAM labelled N_3_-DNFs showed absorption and emission spectra indicative of successful click labelling, whilst the non-modified DNFs gave no observable fluorescence, confirming that non-specific FAM-labelling was not occurring (Supplementary Figure S42). Fluorescence microscopy showed punctate fluorescence (Figure 6B), agarose gel electrophoresis indicated that the dye was successfully linked to the DNA component of the particles (Supplementary Figure S43), and SEM analysis confirmed that the labelling step did not alter DNF structure (Figure 6D). The degree of fluorescent labelling correlated with the concentration of azidomethyl-dUTP **1** used in the RCA reaction (Figure 6E), demonstrating that this approach can be used to control the degree of chemical modification in DNFs. Unlike the previous NHS ester-amine labelling experiments, no background labelling occurred, as none of the components present in a DNF (pyrophosphate anions, encapsulated proteins, and unmodified DNA) can react with an alkyne.

#### CuAAC labelling

**Figure 7.**
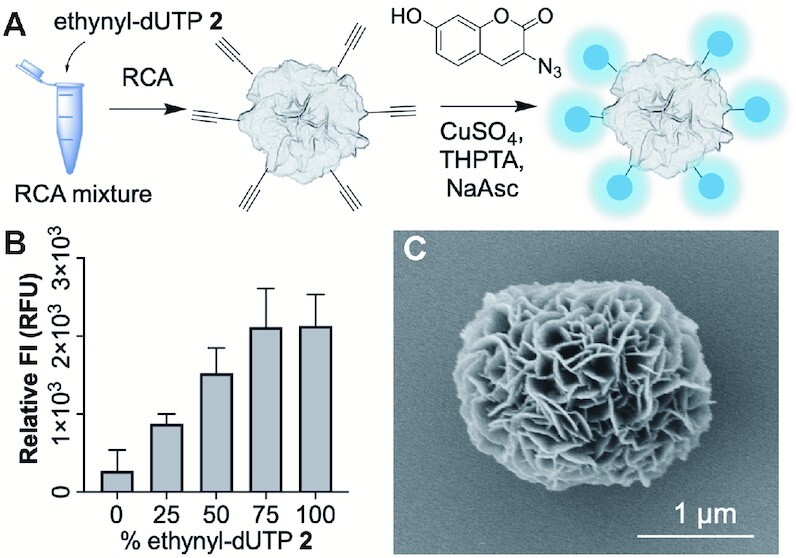
CuAAC labelling of ≡-DNFs (ethynyl-dU DNFs). (**A**) Schematic overview of the methodology; (**B**) Relative coumarin fluorescence (Excitation wavelength: 404 nm, Emission wavelength: 477 nm) of ≡-DNFs prepared with varying amounts of ethynyl-dUTP **2** after CuAAC click labelling with N_3_-coumarin. Error bars represent the standard deviation of three repeat reactions. We also measured a ‘buffer only’ control and subtracted the value from each of the samples. (**C**) SEM image of a ≡-DNF prepared with 100% ethynyl-dUTP and labelled with N_3_-coumarin.

Next, we carried out Cu(I)-catalysed click labelling experiments on alkyne functionalised DNFs (≡-DNFs) prepared using ethynyl-dUTP **2**. The DNFs were labelled with N_3_-coumarin (3-azido-7-hydroxycoumarin), a weakly-fluorescent dye that becomes highly fluorescent following 1,3-cycloaddition to an alkyne to form a triazole (Figure 7). The ≡-DNFs were incubated with N_3_-coumarin, CuSO_4_, THPTA and sodium ascorbate for 2 h. As expected, the fluorescence was ‘turned-on’ in proportion to the amount of ethynyl-dUTP added during RCA (Figure 7B), confirming that covalent labelling was occurring. We observed a levelling off in fluorescence between 80% and 100% ethynyl-dUTP, probably due to quenching between neighbouring fluorophores when they are present at high density. As with the SPAAC reaction, we observed very little background labelling of DNFs that did not contain alkyne groups.

### Combining modified dNTPs with differently modified dNTPs or proteins to attach multiple functionalities to DNFs

The methods described above are suitable for dual labelling of the same DNF construct. Dual labelling can be achieved by adding to the RCA mixture two modified dNTPs with different functionality or using an alkyne or azide-modified dNTP and a lysine rich protein (BSA). This produces DNFs with azide or alkyne groups for click reactions as well as amines for NHS labelling. Using this concept, we simultaneously labelled DNFs prepared using 25% azidomethyl-dUTP **1** and 25% aminopropynyl-dCTP **6** with TAMRA-NHS and FAM-BCN (Supplementary Figure S44). We also carried out dual labelling by a different method, adding both BSA and azidomethyl-dUTP to the RCA mixture and labelling the resulting DNFs with TAMRA-NHS and FAM-BCN (Supplementary Figure S45).

**Figure 8.**
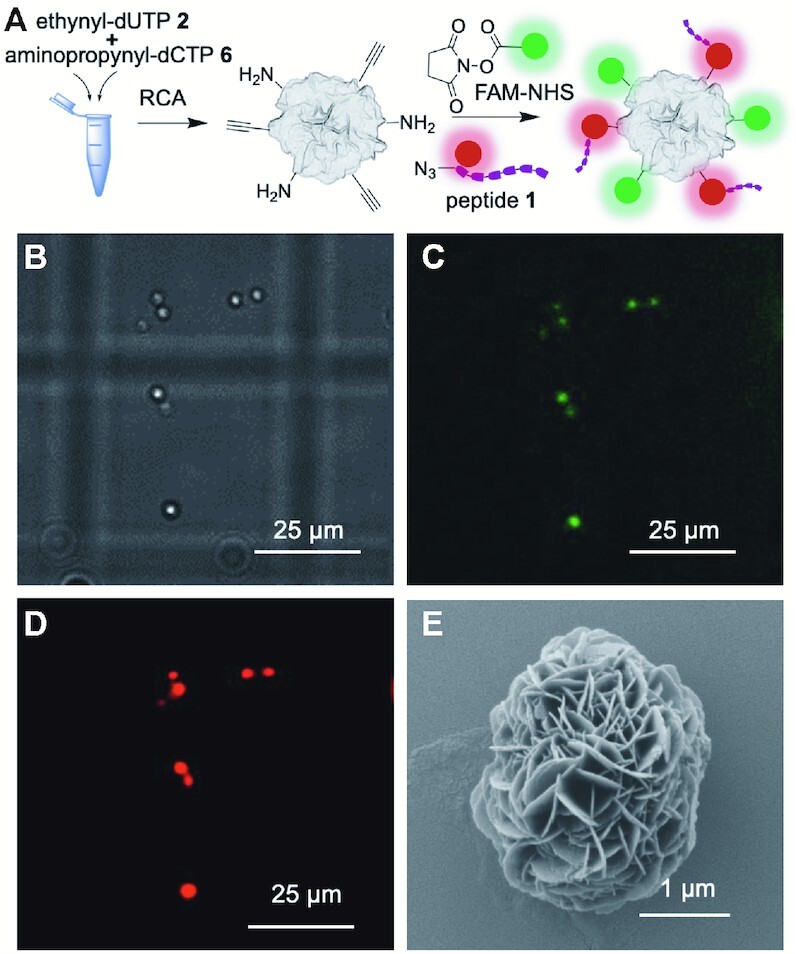
Dual labelling of DNFs prepared using 25% ethynyl-dUTP **2** and 25% aminopropynyl-dCTP **6** then simultaneous functionalisation with an azide/TAMRA modified peptide and FAM-NHS. (**A**) Schematic of the method; (**B**) bright-field image of dual labelled DNFs; (**C**) fluorescence image under 475 nm excitation confirming attachment of FAM; (**D**) fluorescence image at 542 nm excitation confirming attachment of the TAMRA peptide; (**E**) SEM image of dual labelled construct.

To further demonstrate the versatility of this dual labelling approach, we attached a fluorescently labelled peptide to DNFs (Figure 8, peptide **1**, Supplementary Table S2, Supplementary Figure S46). The sequence of amino acids in peptide **1** has been reported to bind HER2 (41), and the peptide was synthesised with a TAMRA dye for visualisation and an N-terminal azide to allow attachment to DNFs. DNFs were prepared using both aminopropynyl-dCTP **6** and ethynyl-dUTP **2** in the RCA reaction mixture, yielding DNFs with amine and alkyne handles. We treated these DNFs with a combination of FAM-NHS and azide-functionalised peptide in a labelling buffer containing CuSO_4_, THPTA, and sodium ascorbate. SEM imaging indicated no perturbation in structure (Figure 8E), and fluorescence microscopy revealed co-localised dyes, which demonstrates that dual labelling had successfully occurred (Figure 8B–D).

### Construction of HER2 targeting nanomaterials

**Figure 9.**
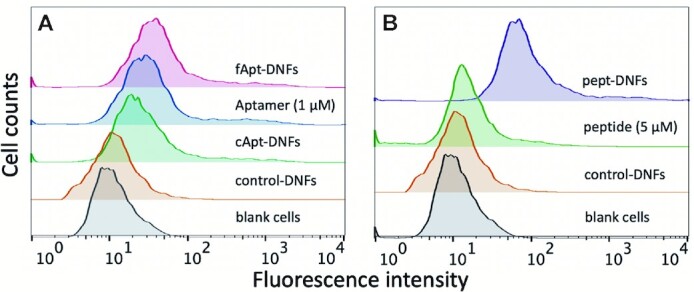
(**A**) Flow cytometry studies comparing the binding of aptamer-DNFs to a HER2^+^ SKBR-3 cell line. Aptamer-DNFs were prepared using the conventional approach (cApt-DNFs) with those prepared using our alternative labelling method (fApt-DNFs). These results demonstrate that chemically attaching the aptamer to the surface of DNFs (fApt-DNFs) results in slightly higher cell binding than when the same aptamer is templated into the DNF (cApt-DNFs); (**B**) DNFs functionalised with a HER2 binding peptide (pept-DNFs) successfully bind to HER2^+^ SKBR-3 cells and perform significantly better than free peptide.

Next, we sought to demonstrate that our DNF labelling methodology can be used to prepare DNFs with selective cell recognition properties. We modified the surface of DNFs with a HER2 binding aptamer (25) and compared them in a HER2^+^ SKBR-3 cell-binding assay with DNFs incorporating the same aptamer prepared by the pure RCA-based approach. The binding of the HER2 aptamer to HER2^+^ SKBR-3 cells has been well characterised previously (42,43), providing a good model system for this study. Conventional aptamer-DNFs (cApt-DNFs), where the DNFs are entirely formed from a concatemer of the HER2 aptamer, were prepared using azidomethyl-dUTP **1** and a cyclic template which encodes for the aptamer during the RCA step (Supplementary Table S1, template 2). We subsequently labelled these DNFs with FAM-BCN. We also prepared functionalised aptamer-DNFs (fApt-DNFs), where the surface of the DNFs is decorated with pendant aptamers. This was done as follows: we used a circular template with a randomly generated ‘non-aptamer’ sequence and azidomethyl-dUTP to prepare non-aptamer N_3_-DNF (Supplementary Table S1, template 3). We then functionalised the DNFs with the HER2 aptamer (synthesised with both FAM and alkyne moieties) using CuAAC labelling, and further treated them with FAM-BCN to enhance their fluorescence (Supplementary Figure S47). SEM was carried out and the results confirmed that the CuAAC labelling step did not perturb DNF structure (Supplementary Figure S48). In addition, we prepared control-DNFs where we labelled the ‘non-aptamer’ DNFs with FAM-BCN instead of the aptamer. We evaluated the binding of these DNFs to HER2^+^ SKBR-3 cells by flow cytometry. The fApt-DNFs showed slightly higher binding than both the cApt-DNFs and control-DNFs (Figure 9A), indicating attachment of aptamers on the surface of DNFs can result in superior cell binding. It is likely that post-synthesis labelling with HER2 aptamers avoids extensive intermolecular interactions between individual aptamers, maintains their complementary shape for receptor binding, and ensures their presentation on the surface of the DNFs where they are needed.

Importantly, as discussed above, our functionalisation approach enables the attachment of non-nucleic acid moieties including peptides to DNFs. To demonstrate the potential of such DNF/peptide constructs, we attached a FAM/azide labelled analogue of A9 (44), a HER2 specific peptide (peptide **2**, Supplementary Table S2, Supplementary Figure S49), to the surface of the DNFs using CuAAC chemistry, generating A9 peptide-modified DNFs (pept-DNFs). We used peptide **2** rather than peptide **1** because preliminary FACS studies using peptide **1** alone (without the DNFs) showed no binding towards SKBR-3 cells, which we presume is due to our additional attachment of TAMRA and the azide. In contrast, peptide **2** alone showed slight binding (Figure 9B). For the click reaction, we prepared the DNFs using the ‘non-aptamer’ template (Supplementary Table S1, template 3), and ethynyl-dUTP **2**. Flow cytometry showed that the pept-DNFs have a higher binding affinity to SKBR-3 cells than fApt-DNFs or cApt-DNFs (Figure 9B). Interestingly, the free peptide only showed weak affinity for the cells; however, when peptide **2** was immobilised on the surface of DNFs, enhanced binding occurred suggesting that multivalency is an important beneficial parameter. Together, these observations highlight the importance of the nanomaterial design and construction strategy when working with aptamers and ligands. They also demonstrate that our labelling methods can generate DNFs with improved properties compared to conventional approaches.

## CONCLUSION

RCA-generated nanostructures including DNFs have great potential as practical scaffolds in the generation of complex hierarchical materials and nanoconstructs, especially if assembled through selective covalent attachment of functional components, within or on the surface of the materials. We show that chemically modified nucleoside triphosphates with small reactive handles can be incorporated at a high-level during RCA without significantly impairing the length, structure or quantity of ssDNA and DNFs produced. We demonstrate that these chemical handles can be modified under mild conditions that do not alter the structural properties of the resulting nanomaterials. We also show that amine rich proteins can be incorporated into DNFs as platforms for functionalisation. Together, these methods offer complementary and orthogonal approaches to functionalise DNFs either on DNA or protein components. Our approach also allows the covalent attachment of functional moieties including aptamers, as well as components that are non-nucleic acid in nature such as peptides, thereby expanding the potential applications of these systems, including selective cell recognition. As such, we have demonstrated a post-assembly functionalisation method that greatly expands the chemistry available to generate novel DNA nanomaterials. Significantly this methodology makes use of commercially available modified dNTP building blocks, avoiding the need for multi-step organic synthesis. Many biologically relevant labels are already available with attached alkyne or azide functionality, or can be readily prepared, further expanding the versatility of this approach.

## DATA AVAILABILITY

The Flow Cytometry experiments generated during the current study are available at the FlowRepository.

## Supplementary Material

gkab720_Supplemental_File
