# Supplementary material for: Expanding the chemical functionality of DNA nanomaterials generated by rolling circle amplification
Source: Nucleic Acids Res. 2021 Aug 17;49(16):9042–52. doi: 10.1093/nar/gkab720 (PMC8450075; doi:10.1093/nar/gkab720)
Supplement: gkab720_Supplemental_File [file gkab720_supplemental_file.pdf]

Expanding the chemical functionality of DNA nanomaterials generated by rolling circle amplification

Ysobel R. Baker,§[a] Liyiwen Yuan,§[a] Jinfeng Chen,[a] Roman Belle,[a] Robert Carlisle,[b] Afaf H. El-Sagheer,[a,c] Tom Brown[a]*

[a] Chemistry Research Laboratory, University of Oxford, 12 Mansfield Road, Oxford, OX1 3TA, UK

[b] Institute of Biomedical Engineering, Old Road Campus Research Building, University of Oxford, Oxford OX3 7DQ, UK

[c] Chemistry Branch Department of Science and Mathematics, Faculty of Petroleum and Mining Engineering, Suez University, Suez 43721, Egypt

E-mail: tom.brown@chem.ox.ac.uk

§ These authors contributed equally to the work

Table of Contents

1. Experimental procedures *3*
1.1. General experimental 3
1.2. Oligonucleotide synthesis of templates and splints 3
1.3. Cyclisation of templates 3
1.4. General RCA procedure 4
1.5. Preparation of functionalised DNFs 4
1.6. Strain promoted alkyne-azide cycloaddition (SPAAC) labelling of N_3-DNFs 4
1.7. Copper catalysed alkyne-azide cycloaddition (CuAAC) labelling of N_3-DNFs and ≡-DNFs 4
1.8. N-Hydroxysuccinimide (NHS) ester labelling of H_2N-DNFs 4
1.9. Dual labelling of DNFs using CuAAC and NHS active ester labelling 4
1.10. Dual labelling of DNFs using SPAAC and NHS active ester labelling 5
1.11. Preparation of H_2N-DNFs using BSA 5
1.12. Labelling of phi29 and BSA with sulfoCy3-NHS 5
1.13. FAM-HER2 aptamer synthesis 5
1.14. TAMRA labelled peptide 1 synthesis 5
1.15. FAM labelled peptide 2 synthesis 6
1.16. Preparation of conventional aptamer-DNFs (cApt-DNFs) 6
1.17. Preparation of functionalised aptamer-DNFs (fApt-DNFs) 6
1.18. Preparation of Pept-DNFs 6
1.19. Preparation of Control-DNFs 7
2. Analysis techniques *7*
2.1. Evaluating the ability of phi29 to incorporate modified dNTPs 7
2.2. ImageJ measurements of particle size distribution 7
2.3. Fluorescence microscopy 7
2.4. Scanning electron microscopy 8
2.5. Measuring the fluoresence as a factor of % modified dNTP 8
2.6. Absorbance and emmision spectra 8
2.7. Cell culture and flow cytometry 8
3. Supplementary tables *9*
4. Supplementary figures *9*
4.1. Agarose gel electrophoresis 9
4.2. Scanning electron microscopy 13
4.3. ImageJ measurements of particles to compare size distribution 16
4.4. Choice of buffer experiments 29

4.5. **Labelling analysis** 29
4.6. **Dual labelling experiments** 32
4.7. **Peptide and aptamer synthesis** 33
5. Mass spectra of DNA templates, DNA splints and peptides used in this study *36*
6. References *41*

1. Experimental procedures

1.1. General experimental

NxGen Phi29 DNA polymerase (phi29) was purchased from Lucigen and T4 DNA ligase was purchased from Promega. Bovine serum albumin (BSA) was purchased from New England BioLabs (NEB). PCR grade sodium salts of dATP, dCTP, dGTP, and dTTP were purchased as 100 mM solutions from Roche. The sodium salts of C8-Alkyne-dUTP (octadiynyl-dUTP **4**) and 5-Ethynyl-dUTP (ethynyl-dUTP **2**) were purchased from Baseclick GmbH, and 5-propargylamino-dCTP (aminopropynyl-dCTP **6**) was purchased as 100 mM solutions from Jena Bioscience. 1 kb DNA ladder was purchased from New England Biolabs (UK). Sulfo-Cyanine3 NHS ester (sulfoCy3-NHS) was purchased from Lumiprobe. SYBR Gold was purchased from ThermoFischer Scientific. Solid supports, standard DNA phosphoramidites, and reagents were purchased from Link Technologies, Glen Research and Applied Biosystems Ltd. NAP-25 and NAP-10 columns were purchased from G.E. Healthcare Life Sciences. Scanning electron microscopy (SEM) consumables were purchased from Agar scientific and used without further treatment. All other chemicals were purchased from Sigma Aldrich. 10 X Tris-borate-EDTA (TBE) buffer was prepared using 108 g of Tris, 55 g of boric acid, 9.3 g of EDTA disodium salt in deionised water (final volume 1 L) and diluted 10 X with MilliQ water before use in denaturing polyacrylamide gel electrophoresis (PAGE), and 20 X for all other experiments.

1.2. Oligonucleotide synthesis of templates and splints

Single stranded oligonucleotides were synthesised using a standard 1.0 μmol phosphoramidite cycle of acid-catalysed detritylation, 50 s coupling, capping and iodine oxidation on an Applied Biosystems 394 automated DNA/RNA synthesiser. The 5′ phosphate was added using a 2-[2-(4,4'-dimethoxytrityloxy)ethylsulfonyl]ethyl-(2-cyanoethyl)-(*N*,*N*-diisopropyl)-phosphoramidite. Stepwise coupling efficiencies and overall yields were determined by automated trityl cation conductivity monitoring facility and in all cases were more than 98%. The oligonucleotides were cleaved from the solid support and deprotected by treatment with concentrated aqueous ammonia solution at room temperature for 1 h followed by heating in a sealed glass vial at 55 °C for 5 h. The aqueous solution of ammonia was then removed by evaporation prior to oligonucleotide purification.

Linear oligonucleotides were purified by reverse-phase high performance liquid chromatography (RP-HPLC) on a Gilson system using a Luna 10 μm C8 100 Å pore Phenomenex 10 x 250 mm column with a gradient of acetonitrile in triethylammonium bicarbonate increasing from 100% buffer A to 50% buffer B over 20 min with a flow rate of 4 mL/min (buffer A: 0.1 M triethylammonium bicarbonate, pH 7.5, buffer B: 0.1 M triethylammonium bicarbonate, pH 7.5 with 50% acetonitrile). The elution of oligonucleotides was monitored by UV absorption at 298 nm. After HPLC purification, oligonucleotides were analysed using RP ultraperformance liquid chromatography-mass spectrometry (UP-LCMS) on either a Bruker micrOTOFTM II focus ESI-TOF MS instrument in ES^- mode or a XEVO G2-QTOF MS instrument in ES^- mode and quantified based upon their absorption at 260 nm using the nearest neighbour molar extinction coefficient. Data were analysed using Waters MassLynx software.

1.3. Cyclisation of templates

Linear oligonucleotides were cyclised using T4 ligase as follows. A solution of the linear template with a 5′ phosphate (2.4 nmol) and splint (4.8 nmol) in 3.9 mL of 1X T4 ligase buffer (30 mM Tris-HCl, pH 7.8 at 25°C, 10 mM $MgCl_2$, 10 mM DTT, and 1 mM adenosine triphosphate (ATP)) was heated to 95 °C for 5 min before cooling to 25 °C at a rate of 0.5 °C/min. T4 DNA ligase (100 μL, 3U/μL) was added and the sample incubated at 25 °C for overnight. The enzyme was denatured by heating to 95 °C for 5 min. The volume of the sample was reduced to approximately 1 mL under reduced pressure and the sample separated by 8% denaturing PAGE. The circularised template band was cut, and the gel was crushed and soaked in water (10 mL) overnight at 37 °C with agitation. After filtration and evaporation of the water, the cyclised templates were desalted using two NAP-25 columns. The resulting circularised templates were analysed using RP-UPLC-MS liquid chromatography-mass spectrometry on either a Bruker micrOTOF^{TM} II focus ESI-TOF MS instrument in ES^- mode or a XEVO G2-QTOF MS instrument in ES^- mode and quantified based upon their absorption at 260 nm using the nearest neighbour molar extinction coefficient. Data were analysed using Waters MassLynx software.

1.4. General RCA procedure

A 10 µL solution containing cyclised template (6 pmol), splint (12 pmol), 2 µL of 10X NexGen phi29 reaction buffer (500 mM Tris-HCl, 100 mM $(NH_4)_2SO_4$, 40 mM DTT, 100 mM $MgCl_2$, pH 7.5 @ 25°C) and 1 µL of 100 mM $MgSO_{4\ aq}$ in MilliQ water in a 200 µL PCR tube was heated to 95 °C for 5 min before cooling to 25 °C at a rate of 0.5 °C/min. To this solution was added the dNTP mix in MilliQ water (9 µL) followed by phi29 (1 µL, 10 000 U/mL). The reaction was incubated in a thermocycler at 30 °C for 20 h, heated to 65 °C for 10 min, and cooled to room temperature.

1.5. Preparation of functionalised DNFs

Modified DNFs were synthesised following the general RCA procedure described above (section 1.4). The dNTP mix was prepared using 100 mM stock solutions of dATP, dTTP, dGTP, dCTP, and modified dNTP and the total volume adjusted to 9 µL with MilliQ water. For example, the dNTP mix for a 50% ethynyl-dUTP **2** DNF would consist of 0.4 µL of 100 mM dATP, 0.4 µL of 100 mM dGTP, 0.4 µL of 100 mM dCTP, 0.2 µL of 100 mM dTTP, 0.2 µL of 100 mM ethynyl-dUTP **2**, and 7.4 µL of water. To avoid errors associated with pipetting small volumes, dNTP mixes were prepared in larger volumes and 9 µL taken from this.

After the RCA step, the samples were diluted with water (80 µL) and transferred to a larger 1.5 mL Eppendorf tube. The PCR tube was washed with 200 µL of water which was added to the Eppendorf tube. This was repeated twice. The DNFs were then collected by centrifugation (20 min, 17 G) and the supernatant discarded. The resulting pellet was resuspended in MilliQ water (0.75 mL) and collected by centrifugation (20 min, 17 G). The process was repeated 3 times.

1.6. Strain promoted alkyne-azide cycloaddition (SPAAC) labelling of N_3-DNFs

DNFs were prepared using the appropriate N_3-dUTP (azidomethyl-dUTP **1** or azidoalkyl-dUTP **5)** as described in section 1.5. The DNFs were then suspended in 20 µL of labelling buffer (10 mM HEPES, pH 8.0, 10 mM $MgSO_4$, 50% v/v DMSO) containing 1 mM of bicyclo[6.1.0]nonyne (BCN) or dibenzocyclooctyne (DBCO) functionalised moiety and gently agitated at room temperature for 2 h. The DNFs were then collected by centrifugation (20 min, 17 G) and the supernatant discarded. The resulting pellet was resuspended in 100 µL of labelling buffer and collected by centrifugation (20 min, 17.0 G). The process was repeated twice with 50% v/v DMSO in MilliQ water and twice with MilliQ water to ensure all salts and small molecules were removed.

1.7. Copper catalysed alkyne-azide cycloaddition (CuAAC) labelling of N_3-DNFs and ≡-DNFs

DNFs were prepared using the appropriate dUTPs as described in section 1.5. The DNFs were then suspended in 20 µL of labelling buffer (10 mM HEPES, pH 8.0, 10 mM $MgSO_4$, 50% v/v DMSO) containing 1 mM alkyne or azide, 0.5 mM $CuSO_4$, 0.5 mM tris-hydroxypropyltriazolylmethylamine (THPTA) and 2.5 mM sodium ascorbate (NaAsc) and gently agitated at room temperature for 2 h. The DNFs were then collected by centrifugation (20 min, 17 G) and the supernatant discarded. The resulting pellet was resuspended in 100 µL of labelling buffer and collected by centrifugation (20 min, 17 G). The process was repeated twice with 50% v/v DMSO in MilliQ water and twice with MilliQ water to ensure all salts and small molecules were removed.

1.8. N-Hydroxysuccinimide (NHS) ester labelling of H_2N-DNFs

DNFs were prepared using the appropriate dUTPs as described in section 1.5. The H_2N-DNFs were suspended in 20 µL of labelling buffer (10 mM HEPES, pH 8.0, 10 mM $MgSO_4$, 50% v/v DMSO) containing 1 mM of the NHS active ester and this was incubated at room temperature for 4 h with gentle agitation. The DNFs were then collected by centrifugation (20 min, 17 G) and the supernatant discarded. The resulting pellet was resuspended in 100 µL of labelling buffer and collected by centrifugation (20 min, 17 G). The process was repeated twice with 50% v/v DMSO in MilliQ water and twice with MilliQ water to ensure all salts and small molecules were removed.

1.9. Dual labelling of DNFs using CuAAC and NHS active ester labelling

DNFs were prepared using the appropriate dUTPs as described in section 1.5. The DNFs were then suspended in 20 µL of labelling buffer (10 mM HEPES, pH 8.0, 10 mM $MgSO_4$, 50% v/v DMSO) containing 1 mM alkyne or azide, 1 mM of NHS active ester, 0.5 mM $CuSO_4$, 0.5 mM THPTA and 2.5 mM NaAsc and this was incubated at room temperature for 2 h with gentle agitation. The DNFs were then collected by centrifugation (20 min, 17 G) and the supernatant discarded. The resulting pellet was resuspended in 100 µL of labelling buffer and collected by centrifugation (20 min, 17 G). The process was repeated twice with 50% v/v DMSO in MilliQ water and twice with MilliQ water to ensure all salts and small molecules were removed.

1.10. Dual labelling of DNFs using SPAAC and NHS active ester labelling

DNFs were prepared using the appropriate dN*TPs as described in section 1.5. The DNFs were then suspended in 20 µL of labelling buffer (10 mM HEPES, pH 8.0, 10 mM $MgSO_4$, 50% v/v DMSO) containing 2.5 mM of BCN or DBCO functionalised moiety and 1 mM of NHS active ester and this was incubated at room temperature for 2 h with gentle agitation. The DNFs were then collected by centrifugation (20 min, 17 G) and the supernatant discarded. The resulting pellet was resuspended in 100 µL of labelling buffer and collected by centrifugation (20 min, 17 G). The process was repeated twice with 50% v/v DMSO in MilliQ water and twice with MilliQ water to ensure all salts and small molecules were removed.

1.11. Preparation of H_2N-DNFs using BSA

DNFs were prepared as described in section 1.5, including BSA (20 mg/mL stock solution) in the dNTP mix. A typical experiment used 0.75 µL of BSA solution (20 mg/mL) per 20 µL RCA volume.

1.12. Labelling of phi29 and BSA with sulfoCy3-NHS

0.1 mg of sulfoCy3-NHS in 25 µL of DMF was added to phi29 (2 µL, 10 000 U/µL) or BSA (1.25 µL, 10 mg/mL) dissolved in 40 uL of protein labelling buffer (62.5 mM HEPES, pH 8, 125 mM $MgSO_4$) and the solution was incubated at 25 °C (500 rmp) for 2 h. This was used for comparison without purification.

1.13. FAM-HER2 aptamer synthesis

The FAM labelled HER2 aptamer was prepared as shown in Figure S47. The modified oligonucleotide was synthesised using automated solid-phase oligonucleotide synthesis on 1.0 µmol scale involving cycles of acid-catalysed detritylation, coupling, capping, and iodine oxidation using an Applied Biosystems 394 synthesiser. Standard DNA phosphoramidites were coupled for 50 s, whereas extended coupling time of 10 min was used for the modified phosphoramidites. The 5′ hexynol was added using a 5-hexyn-1-yl-(2-cyanoethyl)-(*N*,*N*-diisopropyl)-phosphoramidite (Glen Research) and a 5'-(4,4'-dimethoxytrityl)-5-[*N*-(trifluoroacetylaminohexyl)-3- acrylamido]-2'-deoxyuridine, 3'-[(2-cyanoethyl)-(*N*,*N*-diisopropyl)]-phosphoramidite (Link) was used to install the amino C6-dT. Stepwise coupling efficiencies and overall yields were determined by automated trityl cation conductivity monitoring facility and in all cases were more than 98%. The resin was treated with a 10% solution of diethylamine in MeCN (1 mL) for 10 min, washed with MeCN (5 x 1 mL), dried under argon and then cleaved from the solid support and deprotected by treatment with concentrated aqueous ammonia solution by heating in a sealed glass vial at 55 °C for 5 h. The aqueous solution of ammonia was then removed by evaporation prior to oligonucleotide purification. The oligonucleotide was purified by RP-HPLC on a Gilson system using a Luna 10 µm C8 100 Å pore Phenomenex 10 x 250 mm column with a gradient of acetonitrile in triethylammonium bicarbonate increasing from 100% buffer A to 50% buffer B over 20 min with a flow rate of 4 mL/min (see section 1.2 for details). The elution of the oligonucleotide was monitored by UV absorption at 298 nm. After HPLC purification, the oligonucleotide was lyophilised.

FAM-NHS labelling was achieved as follows. 5(6)-FAM-NHS (10 eq) in DMSO (5 µL) was added to 10 nmol oligonucleotide dissolved 1:1 DMSO:carbonate buffer (0.5 M $NaHCO_3$/Na_2CO_3, pH 8.75, 20 µL) and the mixture was incubated at room temperature (RT) for 2 h. The mixture was desalted using a NAP gel filtration column and purified by RP-HPLC as before to give the FAM-labelled alkyne HER2 aptamer which was analysed using RP-UPLC-MS on a XEVO G2-QTOF MS instrument in ES^- mode and quantified based upon their absorption at 260 nm using the nearest neighbour molar extinction coefficient. Data were analysed using Waters MassLynx software.

After purification 40 µM aptamer was annealed in 100 mM K^+ buffer (10 mM PO_4^{3-}, 100 mM KCl, pH 7.2) by heating to 95 °C for 5 min and cooled to 20 °C at a rate of 0.5 °C/min before being used in click reactions.

1.14. TAMRA labelled peptide 1 synthesis

The peptide was prepared as shown in Figure S46. The peptide backbone was synthesised on a 100 µmol scale by standard Fmoc solid phase peptide synthesis on rink amide resin using a Liberty Blue automated microwave peptide synthesiser (CEM Corporation) equipped with commercially available amino acids including Fmoc-Ahx-OH (Sigma). After synthesis 20 mg of resin was washed with DMF before a solution of TAMRA-NHS active ester (5 mg) and DIPEA (100 µL) in DMF (1 mL) was added and the resin agitated at RT overnight. The resin was washed with DMF (3 x 1mL), CH_2Cl_2 (3 x 1 mL) and Et_2O (2 x 1 mL) before drying under vacuum. The peptide was then cleaved from the resin using a 95:2.5:2.5 mix of TFA:triisopropylsilane:H_2O (2 mL) for 4 h and precipitated with ice-cold Et_2O. After lyophilisation the peptide was purified using a Perkin Elmer HPLC system equipped with a 250 x 21.2 mm Phenomenex Gemini NX-C18 10 u column using a gradient of 0 – 80% buffer D in buffer C over 30 min with a flow rate of 6 mL/min (buffer C: 0.1% TFA in H_2O, buffer D: 0.1% TFA in acetonitrile). The elution of the peptide was monitored by UV absorption at 254 nm and lyophilised. A solution of 6-azidohexanoic acid NHS ester (1 mg) in DMF (100 µL) was added to a solution of *N*,*N*-diisopropylethylamine (10 µL) and peptide (0.1 µmol) dissolved in DMF (100 µL) and the reaction was incubated at 55 °C for 4 h. The reaction was then evaporated to dryness, purified using the same HPLC gradient as above, and lyophilised to give a dark purple powder. The peptide was then analysed using Waters LCT (TOF) LCMS using a gradient of MeCN in water with 0.1% formic acid.

1.15. FAM labelled peptide 2 synthesis

The peptide was prepared as shown in Figure S49. Standard Fmoc solid phase peptide synthesis on rink amide resin using a Liberty Blue automated microwave peptide synthesiser (CEM Corporation) equipped with commercially available amino acids including Fmoc-azidolysine (Fluorochem) and Fmoc-amino-3,6 dioxaoctanoic acid (Fluorochem) was used to synthesise the peptide backbone on a 100 µmol scale. Resin equivalent to a 1 µmol synthesis of the peptide was then cleaved from the resin using a 95:2.5:2.5 mix of TFA:triisopropylsilane:H_2O (2 mL) for 4 h and precipitated with ice-cold Et_2O. After lyophilisation the peptide was purified using a on a Perkin Elmer HPLC system equipped with a 250 x 21.2 mm Phenomenex Gemini NX-C18 10 u column using a gradient of 0 – 80% buffer D in buffer C over 30 min with a flow rate of 6 mL/min (buffer C: 0.1% TFA in H_2O, buffer D: 0.1% TFA in acetonitrile). The elution of the peptide was monitored by UV absorption at 254 nm. The fractions containing the peptide were combined, lyophilised and re-dissolved in 90 µL of carbonate buffer (0.5 M $NaHCO_3$/Na_2CO_3, pH 8.7) and 90 µL of 20 mM FAM-NHS in DMF was added. The reaction was incubated at RT for overnight. Free dye and salts were removed using a NAP-10 column (eluting with 20% aqueous MeCN), the sample was purified using the same HPLC gradient as above, and lyophilised to give a dark orange powder. The peptide was then analysed using Waters LCT (TOF) LCMS using a gradient of MeCN in water with 0.1% formic acid. The peptide was freeze dried and dissolved in Dulbecco's phosphate buffered saline (DPBS) prior to cell work or dissolved in DMSO for DNF labelling experiments. The concentration of the peptide was determined using the Beer-Lambert law and measuring the absorbance at 495 nm.

1.16. Preparation of conventional aptamer-DNFs (cApt-DNFs)

A 10 µL solution containing cyclised template 2 (6 pmol), splint 2 (12 pmol), 2 µL of 10X NexGen phi29 reaction buffer (500 mM Tris-HCl, 100 mM $(NH_4)_2SO_4$, 40 mM DTT, 100 mM $MgCl_2$, pH 7.5 @ 25°C) and 3 µL of 100 mM $MgSO_{4\,aq}$ in MilliQ water in a 200 µL PCR tube was heated to 95 °C for 5 min before cooling to 25 °C at a rate of 0.5 °C/min. To this solution was added the dNTP mix (0.4 µL of 100 mM dATP, 0.4 µL of 100 mM dGTP, 0.4 µL of 100 mM dCTP, 0.3 µL of 100 mM dTTP, 0.1 µL of 100 mM azidomethyl-dUTP **1**, and 7.4 µL of water in MilliQ water) followed by phi29 (1 µL, 10 000 U/mL). The reaction was incubated in a thermocycler at 30 °C for 6 h, heated to 65 °C for 10 min, and cooled to RT.

After the RCA step, the samples were diluted with water (80 µL) and transferred to a larger 1.5 mL Eppendorf tube. The PCR tube was washed with 200 µL of water which was added to the Eppendorf tube. This was repeated twice. The DNFs were then collected by centrifugation (20 min, 17 G) and the supernatant discarded. The resulting pellet was resuspended in MilliQ water (0.75 mL) and collected by centrifugation (20 min, 17 G). The process was repeated 4 times. The N_3-DNFs were then labelled with FAM-BCN as outlined in section 1.6.

1.17. Preparation of functionalised aptamer-DNFs (fApt-DNFs)

A 10 µL solution containing cyclised template 3 (6 pmol), splint 3 (12 pmol), 2 µL of 10X NexGen phi29 reaction buffer (500 mM Tris-HCl, 100 mM $(NH_4)_2SO_4$, 40 mM DTT, 100 mM $MgCl_2$, pH 7.5 @ 25°C) and 3 µL of 100 mM $MgSO_{4\,aq}$ in MilliQ water in a 200 µL PCR tube was heated to 95 °C for 5 min before cooling to 25 °C at a rate of 0.5 °C/min. To this solution was added the dNTP mix (0.4 µL of 100 mM dATP, 0.4 µL of 100 mM dGTP, 0.4 µL of 100 mM dCTP, 0.3 µL of 100 mM dTTP, 0.1 µL of 100 mM azidomethyl-dUTP **1**, and 7.4 µL of water in MilliQ water) followed by phi29 (1 µL, 10 000 U/mL). The reaction was incubated in a thermocycler at 30 °C for 6 h, heated to 65 °C for 10 min, and cooled to room temperature.

After the RCA step, the samples were diluted with water (80 µL) and transferred to a larger 1.5 mL Eppendorf tube. The PCR tube was washed with 200 µL of water which was added to the Eppendorf tube. This was repeated twice. The DNFs were then collected by centrifugation (20 min, 17 G) and the supernatant discarded. The resulting pellet was resuspended in MilliQ water (0.75 mL) and collected by centrifugation (20 min, 17 G). The process was repeated 4 times.

The N_3-DNFs were then suspended in 20 µL of labelling buffer (10 mM HEPES, pH 8.0, 10 mM $MgSO_4$, 50% v/v DMSO) containing 0.5 mM $CuSO_4$, 0.5 mM THPTA and 2.5 mM NaAsc and 20 µL of the 40 µM annealed aptamer solution (section 1.13) was added. The solution was gently agitated at RT for 2 h. The DNFs were then collected by centrifugation (20 min, 17 G) and the supernatant discarded. The resulting pellet was resuspended in 100 µL of labelling buffer and collected by centrifugation (20 min, 17 G). The process was repeated twice with MilliQ water to ensure all salts and small molecules were removed. The DNFs were then treated with FAM-BCN as outlined in section 1.6. This second labelling was performed to functionalise any remaining azides that may not have been accessible by the aptamer and improve the brightness of the particles.

1.18. Preparation of Pept-DNFs

A 10 µL solution containing cyclised template 3 (6 pmol), splint 3 (12 pmol), 2 µL of 10X NexGen phi29 reaction buffer (500 mM Tris-HCl, 100 mM $(NH_4)_2SO_4$, 40 mM DTT, 100 mM $MgCl_2$, pH 7.5 @ 25°C) and 3 µL of 100 mM $MgSO_{4\,aq}$ in MilliQ water in a 200 µL PCR tube was heated to 95 °C for 5 min before cooling to 25 °C at a rate of 0.5 °C/min. To this solution was added the dNTP mix (0.4 µL of 100 mM dATP, 0.4 µL of 100 mM dGTP, 0.4 µL of 100 mM dCTP, 0.3 µL of 100 mM dTTP, 0.1 µL of 100 mM ethynyl-dUTP **2**, and 7.4 µL of water in MilliQ water) followed by phi29 (1 µL, 10 000 U/mL). The reaction was incubated in a thermocycler at 30 °C for 6 h, heated to 65 °C for 10 min, and cooled to room temperature.

After the RCA step, the samples were diluted with water (80 µL) and transferred to a larger 1.5 mL Eppendorf tube. The PCR tube was washed with 200 µL of water which was added to the Eppendorf tube. This was repeated twice. The DNFs were then collected by centrifugation (20 min, 17 G) and the supernatant discarded. The resulting pellet was resuspended in MilliQ water (0.75 mL) and collected by centrifugation (20 min, 17 G). The process was repeated 4 times.

The HER2 peptide **2** (section 1.15) was then clicked to the ≡-DNFs as outlined in section 1.7. The resulting DNFs were then further treated with FAM-N_3 as described in section 1.7. This second labelling was performed to functionalise any remaining alkynes that may not have been accessible by the aptamer and improve the brightness of the particles.

1.19.Preparation of Control-DNFs

A 10 µL solution containing cyclised template 3 (6 pmol), splint 3 (12 pmol), 2 µL of 10X NexGen phi29 reaction buffer (500 mM Tris-HCl, 100 mM $(NH_4)_2SO_4$, 40 mM DTT, 100 mM $MgCl_2$, pH 7.5 @ 25°C) and 3 µL of 100 mM $MgSO_{4\ aq}$ in MilliQ water in a 200 µL PCR tube was heated to 95 °C for 5 min before cooling to 25 °C at a rate of 0.5 °C/min. To this solution was added the dNTP mix (0.4 µL of 100 mM dATP, 0.4 µL of 100 mM dGTP, 0.4 µL of 100 mM dCTP, 0.3 µL of 100 mM dTTP, 0.1 µL of 100 mM azidomethyl-dUTP **1**, and 7.4 µL of water in MilliQ water) followed by phi29 (1 µL, 10 000 U/mL). The reaction was incubated in a thermocycler at 30 °C for 20 h, heated to 65 °C for 10 min, and cooled to room temperature.

After the RCA step, the samples were diluted with water (80 µL) and transferred to a larger 1.5 mL Eppendorf tube. The PCR tube was washed with 200 µL of water which was added to the Eppendorf tube. This was repeated twice. The DNFs were then collected by centrifugation (20 min, 17 G) and the supernatant discarded. The resulting pellet was resuspended in MilliQ water (0.75 mL) and collected by centrifugation (20 min, 17 G). The process was repeated 4 times. The N_3-DNFs were then labelled with FAM-BCN as outlined in section 1.6.

2. Analysis techniques

2.1. Evaluating the ability of phi29 to incorporate modified dNTPs

RCA reactions were performed as described in the general RCA procedure above, using cyclised template 1 and splint 1 (Table S1). These were diluted with 80 µL of water (5X dilution) and then thoroughly mixed with a pipette. The precipitate was separated by centrifugation (20 min, 17 G) and the supernatant transferred to a fresh tube. The precipitate was then washed with MilliQ water (3 x 100 µL) and stored in 100 µL of TBE buffer overnight, breaking down the particles and releasing the DNA. These solutions were analysed as described below.

To compare the amount of precipitated DNA, 5 µL of the particle solution was added to 100 µL of TBE buffer containing 1X SYBR Gold and 0.1% Triton X-100 in a 96 well plate (F-bottom, black, Fluotrac, med. Binding Greiner). The plate was loaded into a CLARIOstar microplate reader and incubated for 20 min with intermittent shaking (300 rpm, 1 min, 5 times) before taking a reading (Excitation: 495-8; Dichroic filter: 514.2) The same gain and focal height were used for all readings and all experiments were performed in triplicate. Data is plotted as a percentage fluorescence signal relative to DNFs prepared with unmodified dNTPs.

To compare the samples using agarose gel electrophoresis 10 µL of each solution was mixed with 2 µL of 6X blue loading dye NEB) and visualised using 0.8% agarose gel cast with TBE buffer containing 0.25X SYBR Gold dye. The gels were run at room temperature (126 V) and imaged using a G:Box (Syngene).

2.2. ImageJ measurements of particle size distribution

All DNFs with clearly identifiable edges were measured using ImageJ software along the horizontal axis at their widest point. DNFs where the edges could not be clearly identified (due to stacking) were not included in the analysis.

2.3. Fluorescence microscopy

Fluorescence microscopy was performed using a API DeltaVision Core Widefield Microscope using either the FITC channel (λ_{Ex} = 455 nm, bandwidth = 28nm; (λ_{Em} = 525 nm, bandwidth = 48) or the TRITC channel (λ_{Ex} = 542 nm, bandwidth 27; (λ_{Em} = 597 nm, bandwidth = 45). A suspension of DNFs in MilliQ water was loaded into a glass haemocytometer prior to imaging.

2.4. Scanning electron microscopy

Scanning electron microscopy (SEM) analysis was performed using a Zeiss Sigma 300 Field Emission Gun Scanning Electron Microscope (FEG-SEM). DNFs were suspended in MilliQ water and 10 µL was added to the surface of a silicon wafer chip which was dried at 50 °C for 15 min. This was then splutter coated with gold before imaging.

2.5. Measuring the fluoresence as a factor of % modified dNTP

The labelled DNFs (section 1.6-1.8) were stored in 50 µL of TBE buffer overnight, breaking down the particles and releasing the DNA. 10 µL of this solution was added to 40 µL of TBE buffer containing 0.1% Triton X-100 in a 96 well plate (F-bottom, black, Fluotrac, med. Binding Greiner). The plate was loaded into a CLARIOstar microplate reader and incubated for 20 min with intermittent shaking (300 rpm, 1 min, 5 times) before taking a reading. The same gain and focal height were used for all readings and all experiments were performed in triplicate. The setting used for the FAM labelling were: Excitation 483-14, Emission: 530-30; For TAMRA Excitation: 535-20, Emission: 585-20; and for Coumarin: Excitation: 404-15, Emission: 477-20.

2.6. Absorbance and emmision spectra

The labelled DNFs (section 1.6-1.8) were stored in 50 µL of TBE buffer overnight, breaking down the particles and releasing the DNA. 10 µL of this solution was added to 40 µL of TBE buffer containing 0.1% Triton X-100 in a 96 well plate (F-bottom, black, Fluotrac, med. Binding Greiner). Before each measurement the 96-well plate was shaken three times 10 minutes apart (1 min, 300 rpm). The fluorescence spectra were obtained by scanning the excitation and emission wavelengths. The parameters used were Excitation: scanned from 420 to 520 nm, with a bandwidth of 10.0 nm; Emission: scanned from 500 to 600 nm, with a bandwidth of 10.0 nm.

2.7. Cell culture and flow cytometry

SKBR-3 Cells were purchased from ATCC and cultured in McCoy's 5A (Modified) medium supplemented with 15% (v/v) fetal bovine serum (FBS) (heat-inactivated) in a cell culture incubator at 37 °C with 5% CO_2. For flow cytometry, 5 x 10^5 SK-BR3 cells were seeded into a 12-well plate in cell culture medium supplemented with 15% (v/v) FBS and left overnight to attach. Then the cells were washed with DPBS before being blocked by incubation in 1% BSA (w/v) in 460 µL of DPBS at 37 °C for 5 min. 40 µL of labelled RCA product was then added to each well and the cells were incubated at 37 °C for 40 min with agitation. The cells were rinsed with DPBS 5 times, gently scraped off the plate, and suspended in 500 µL of DPBS for flow cytometry analysis. Flow cytometry was performed using a Becton Dickinson FACSCalibur flow cytometer using the FITC channel settings and the data was analysed using FlowJo software.

3. Supplementary tables

Table S1. Sequences of oligonucleotides used in this study. P represents a 5' phosphate, X a 5' hexynol modification and Y an amino C6-dT modifier labelled with FAM-NHS (See Figure S47). Template 1 and splint 1 have been reported previously (1) and were used for all experiments unless otherwise stated. We have used template 2, template 3, splint 2, and splint 3 previously. (2) See figure S50-59 for HPLC traces and mass spectra for oligonucleotides and the cyclised templates.

Oligonucleotide	Sequence	Calculated mass	Found mass
template 1	PCATGTGCTATAGCCTGCTGCTGCAGCGATACGCGTATCGCTATGGCATATCGTACGATATGCCGCAGC AGCAGTCGTTTTACC	25597	25601
template 2	PTTACCCCACACCGCTGCCCCCACACCGCTGCCCCCACACCGCTGCCTTAC	15012	15014
template 3	PTCCCAATTGGGTACGCAGTACCCACAGCAGATGTGACTGTGAATCGTGAC	15473	15474
splint 1	GGCTATAGCACATGGGTAAAACGAC	7724	7724
splint 2	TGGGGTAAGTAAGGCA	5010	5011
splint 3	CAATTGGGAGTCACGATT	5539	5540
5'-alkyne, FAM-modified-HER2 aptamer	XTTTYGCAGCGGTGTGGGGGCAGCGGTGTGGGGGCAGCGGTGTGGGG	15216	15217

Table S2. Sequences of peptides used in this study. Both peptides have C-terminal amides. For detailed structures see Figures S46 and S49. TAMRA = tetramethylrhodamine, FAM = fluorescein, Ahx = 6-aminohexanoic acid residue. Lys($C6N_3$) represents a lysine residue labelled with 6-azidohexanoic acid NHS. See figure S59 and S60 for HPLC traces and mass spectra of the modified peptides. The amino acid sequences for both peptides have been reported previously but different modifications were used. (3,4)

Peptide	Sequence	Calculated mass	Found mass
peptide 1	TAMRA-Lys($C6N_3$)-Ahx-LTVSPWY	1655.9	1655.3
peptide 2	FAM-Lys(N_3)-PEG-QDVNTAVAW	1658.7	1657.8

4. Supplementary figures

4.1. Agarose gel electrophoresis

The ability of phi29 to incorporate each of the modified dNTPs during RCA was also evaluated using agarose gel electrophoresis. (Figure S1-6). After RCA the DNFs were separated from the RCA mixture by centrifugation. The 'free' DNA (i.e. DNA not incorporated into DNFs) and DNA in the DNFs were then separated. In these experiments the ratio of modified to canonical dNTP was varied keeping the total concentration at 2 mM. As expected the RCA reactions all produced large, slow migrating products that appear in the wall of the gel well. A second discrete faster moving band is often observed which we have assigned as double stranded products. A proposed mechanism for this is given in our recent paper (2) and is shown below. Loading the gels with the very long DNA constructs is difficult as they do not enter the matrix readily due to their size. Therefore, the fluorescence data in Figure 3 (manuscript) is more quantitative, but does not give information on the size of the RCA products.

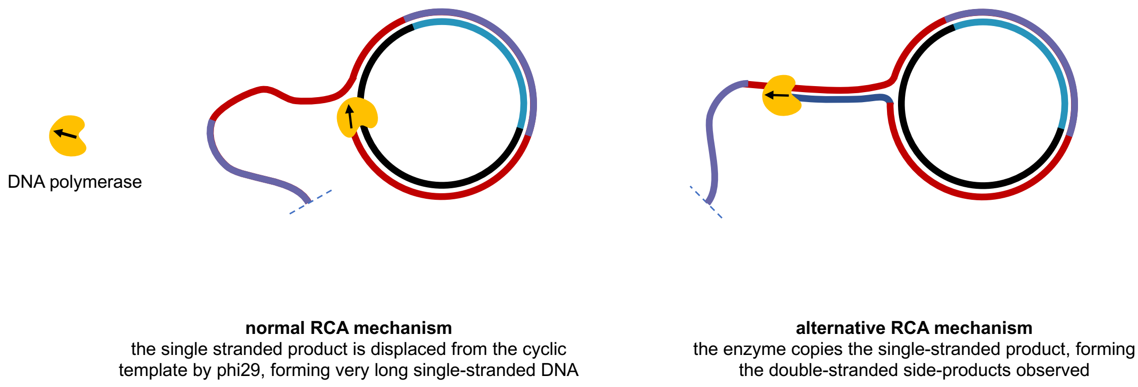

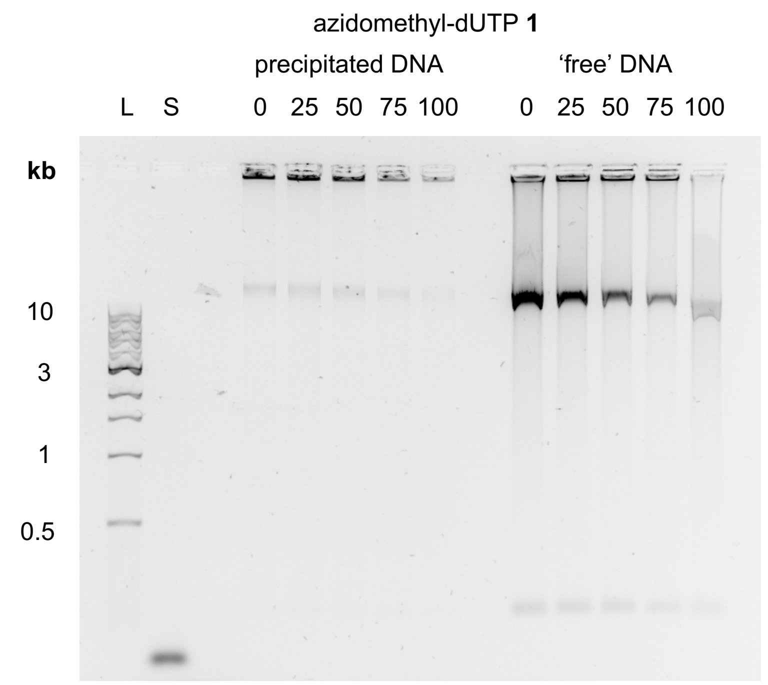

Figure S1. Agarose gel electrophoresis analysis of RCA reactions when dTTP was substituted with different percentages of 5-azidomethyl-dUTP **1** as indicated above the lanes. In all cases the total dNTP concentration was kept at 2 mM. L) 1 kb DNA ladder; S) splint 1; These gels show how the modified dUTP affects phi29 efficiency and processivity.

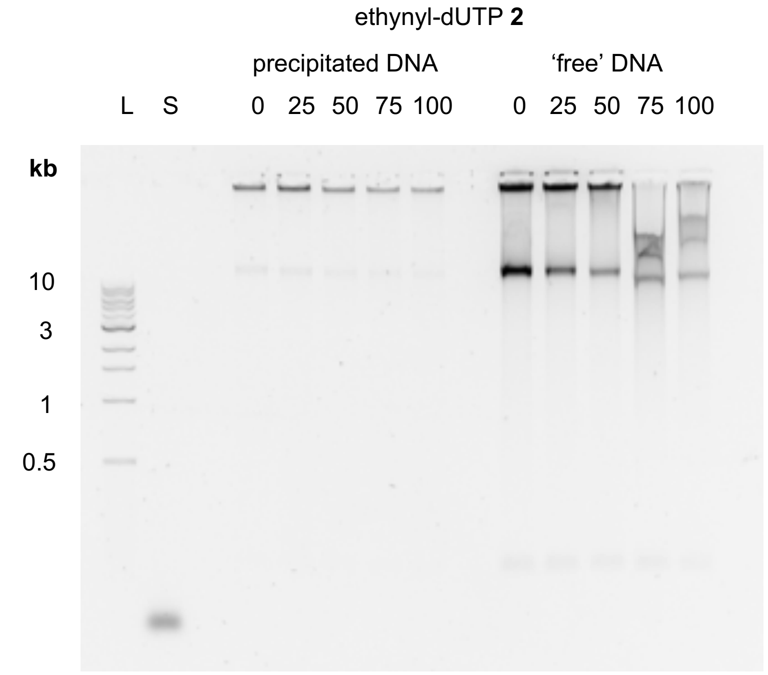

Figure S2. Agarose gel electrophoresis analysis of RCA reactions when dTTP was substituted with different percentages of ethynyl-dUTP **2** as indicated above the lanes. In all cases the total dNTP concentration was kept at 2 mM. L) 1 kb DNA ladder; S) splint 1; These gels show how the modified dUTP affects phi29 efficiency and processivity.

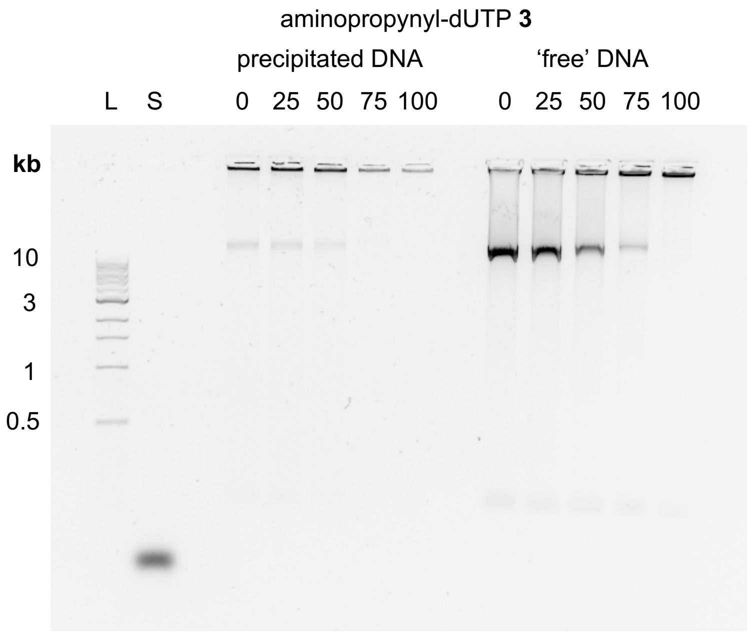

Figure S3. Agarose gel electrophoresis analysis of RCA reactions when dTTP was substituted with different percentages of aminopropynyl-dUTP **3** as indicated above the lanes. In all cases the total dNTP concentration was kept at 2 mM. L) 1 kb DNA ladder; T) an annealed mixture of template 1 and splint 1; These gels show how the modified dUTP affects phi29 efficiency and processivity.

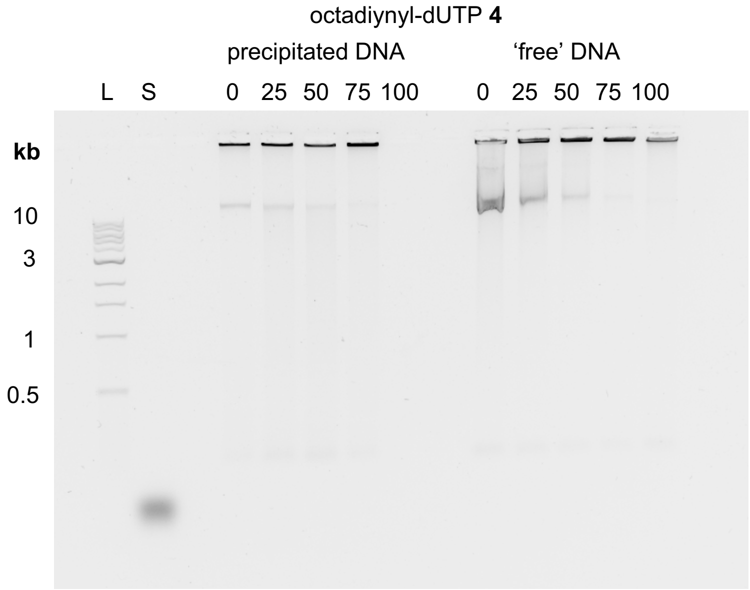

Figure S4. Agarose gel electrophoresis analysis of RCA reactions when dTTP was substituted with different percentages of octadiynyl-dUTP **4** as indicated above the lanes. In all cases the total dNTP concentration was kept at 2 mM. L) 1 kb DNA ladder; S) splint 1; These gels show how the modified dUTP affects phi29 efficiency and processivity.

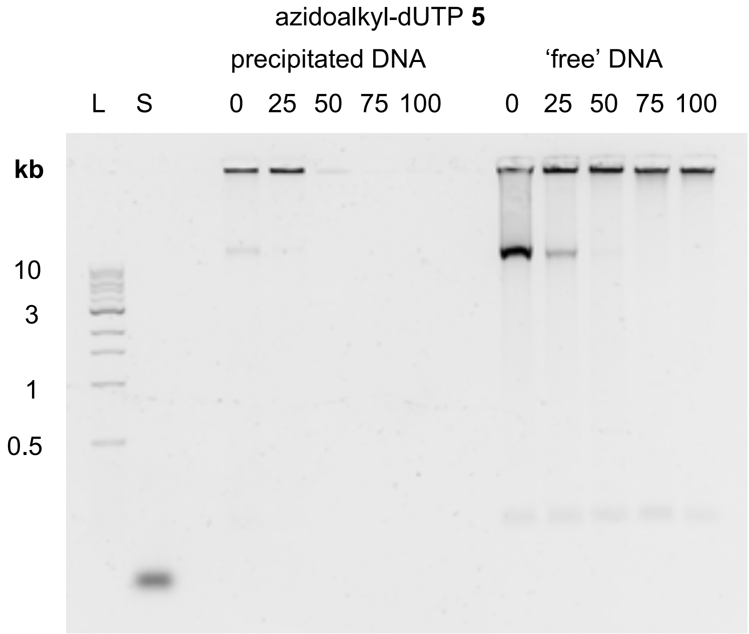

Figure S5. Agarose gel electrophoresis analysis of RCA reactions when dTTP was substituted with different percentages of azidoalkyl-dUTP **5** as indicated above the lanes. In all cases the total dNTP concentration was kept at 2 mM. L) 1 kb DNA ladder; S) splint 1; These gels show how the modified dUTP affects phi29 efficiency and processivity.

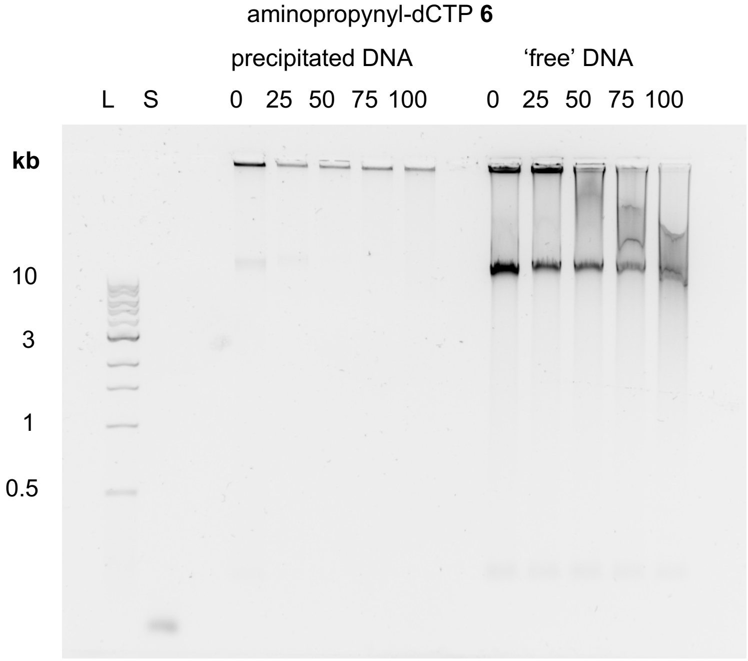

Figure S6. Agarose gel electrophoresis analysis of RCA reactions when dCTP was substituted with different percentages of aminopropynyl-dCTP **6** as indicated above the lanes. In all cases the total dNTP concentration was kept at 2 mM. L) 1 kb DNA ladder; S) splint 1; These gels show how the modified dCTP affects phi29 efficiency and processivity.

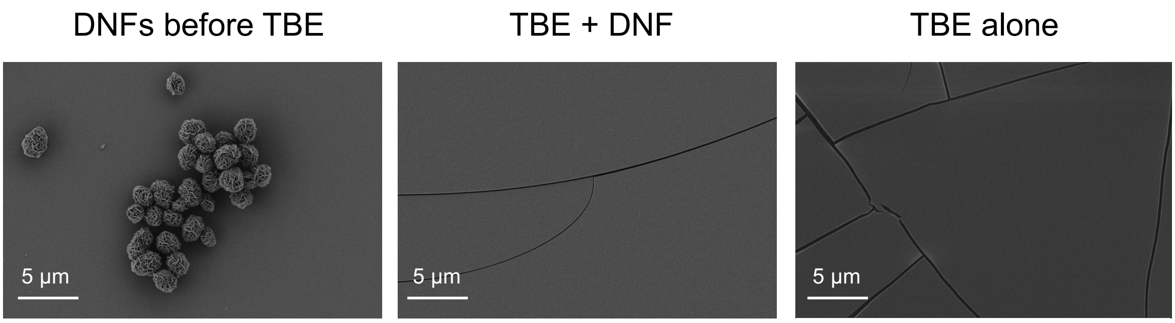

Figure S7. SEM images of a DNF before and after treatment with TBE. No structures were observed following TBE treatment.

4.2. Scanning electron microscopy

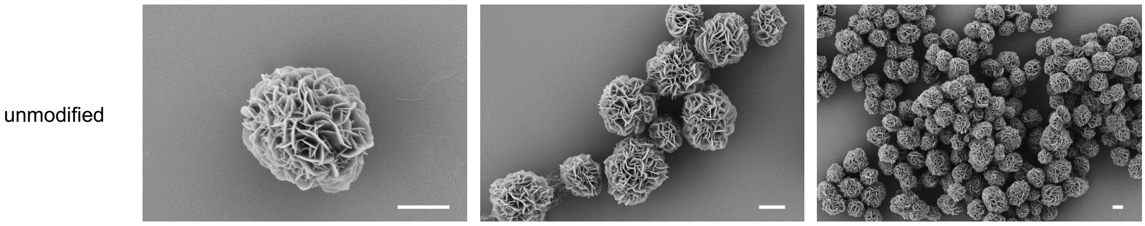

Figure S8. SEM images of unmodified DNFs at different magnifications. Scale bar represents 1 μm.

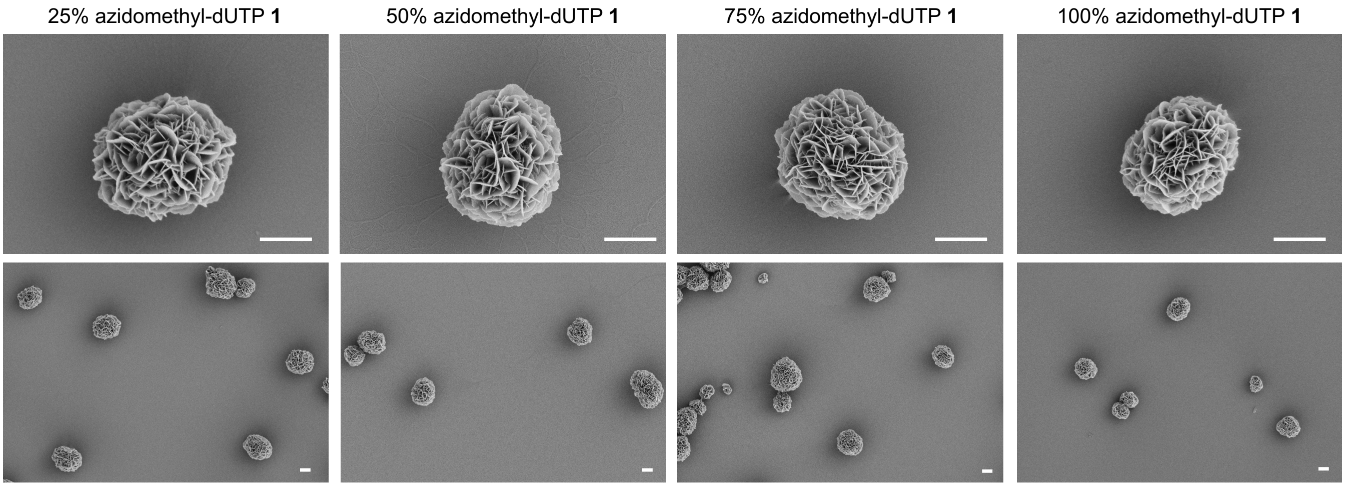

Figure S9. SEM images of DNFs produced with different proportions of azidomethyl-dUTP **1** to dTTP. In all cases the total dNTP concentration was kept at 2 mM and the % azidomethyl-dUTP **1** is indicated above the images. Figure S8 shows a typical unmodified DNF. Scale bars represent 1 μm.

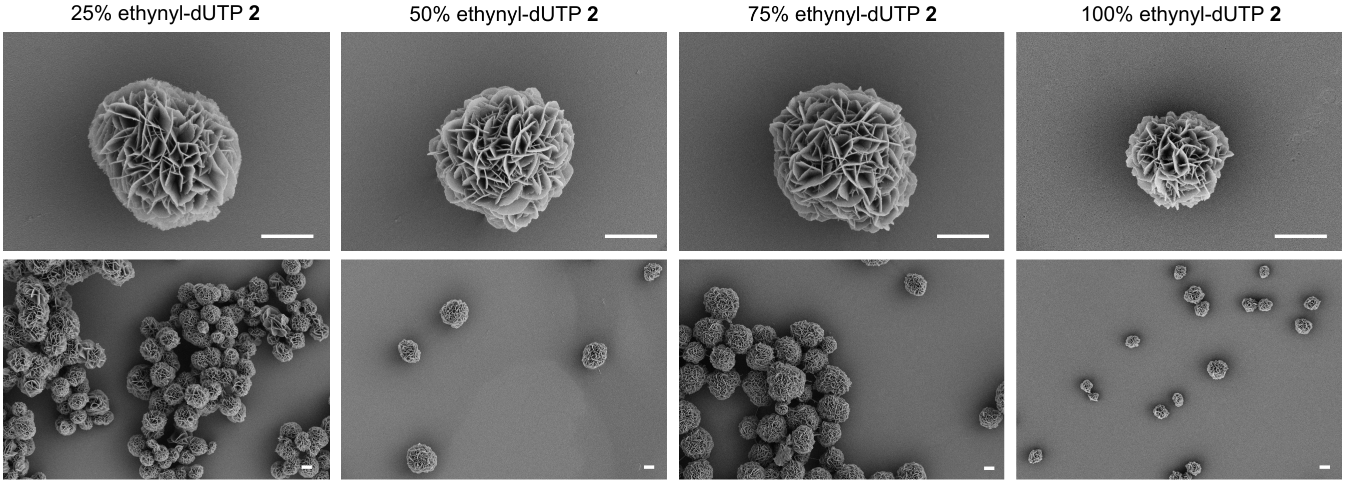

Figure S10. SEM images of DNFs produced with different proportions of ethynyl-dUTP **2** to dTTP. In all cases the total dNTP concentration was kept at 2 mM and the % ethynyl-dUTP **2** is indicated above the images. Figure S8 shows a typical unmodified DNF. Scale bars represent 1 μm.

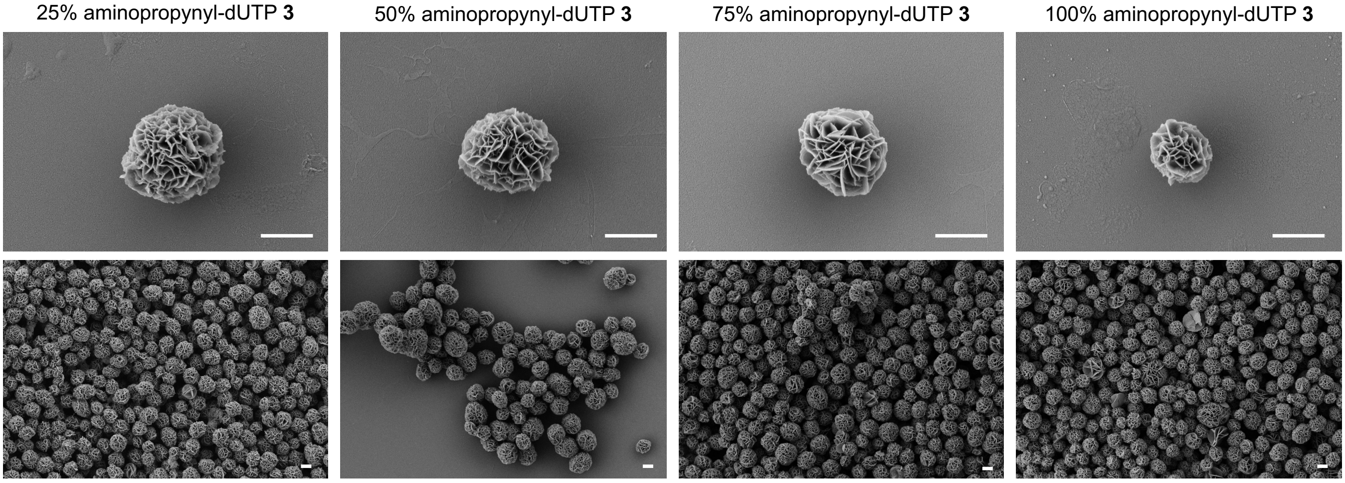

Figure S11. SEM images of DNFs produced with different proportions of aminopropynyl-dUTP **3** to dTTP. In all cases the total dNTP concentration was kept at 2 mM and the % aminopropynyl-dUTP **3** is indicated above the images. Scale bars represent 1 μm.

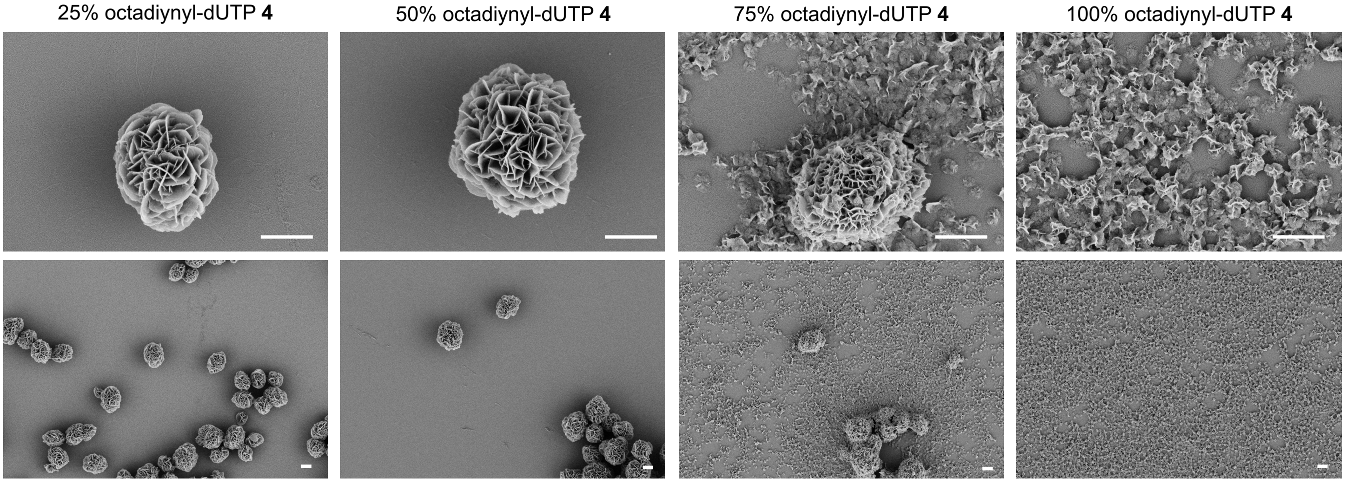

Figure S12. SEM images of DNFs produced with different proportions of octadiynyl-dUTP **4** to dTTP. In all cases the total dNTP concentration was kept at 2 mM and the % octadiynyl-dUTP **4** is indicated above the images. Scale bars represent 1 μm.

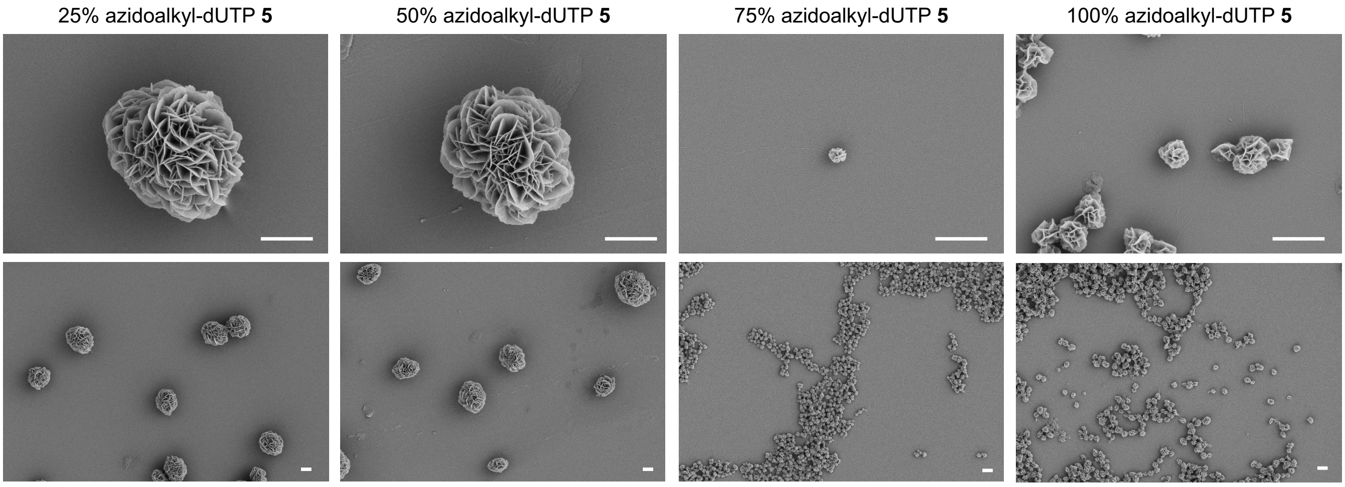

Figure S13. SEM images of DNFs produced with different proportions of azidoalkyl-dUTP **5** to dTTP. In all cases the total dNTP concentration was kept at 2 mM and the % azidoalkyl-dUTP **5** is indicated above the images. Scale bars represent 1 μm.

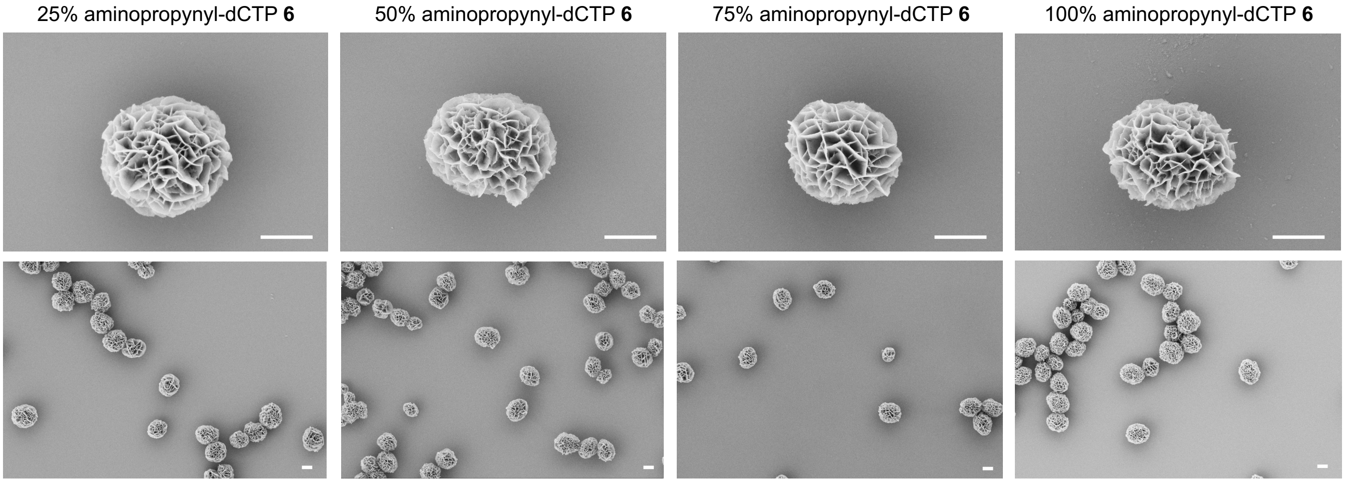

Figure S14. SEM images of DNFs produced with different proportions of aminopropynyl-dCTP **6** to dCTP. In all cases the total dNTP concentration was kept at 2 mM and the % aminopropynyl-dCTP **6** is indicated above the images. Figure S8 shows a typical unmodified DNF. Scale bars represent 1 μm.

4.3. ImageJ measurements of particles to compare size distribution

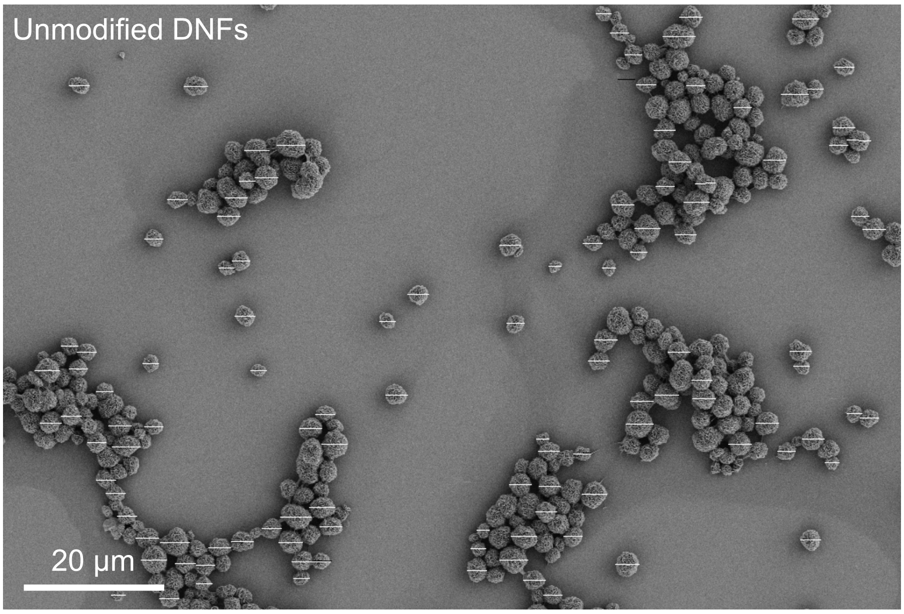

Figure S15. Example SEM image of precipitate formed during phi29 catalysed RCA using unmodified triphosphates. Average size of particles measured using ImageJ: 2.81 ± 0.53 µm, n = 120. White lines indicate the individual measurements taken from this image for analysis.

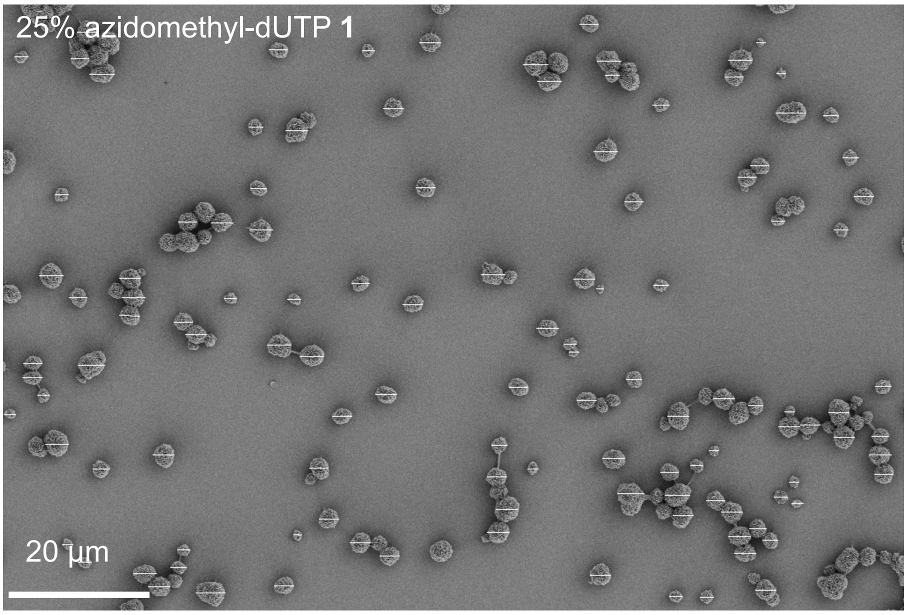

Figure S16. Example SEM image of precipitate formed during phi29 catalysed RCA using 25% azidomethyl-dUTP **1**. Average size of particles measured using ImageJ: 2.48 ± 0.64 µm, n = 119. White lines indicate the individual measurements taken from this image for analysis.

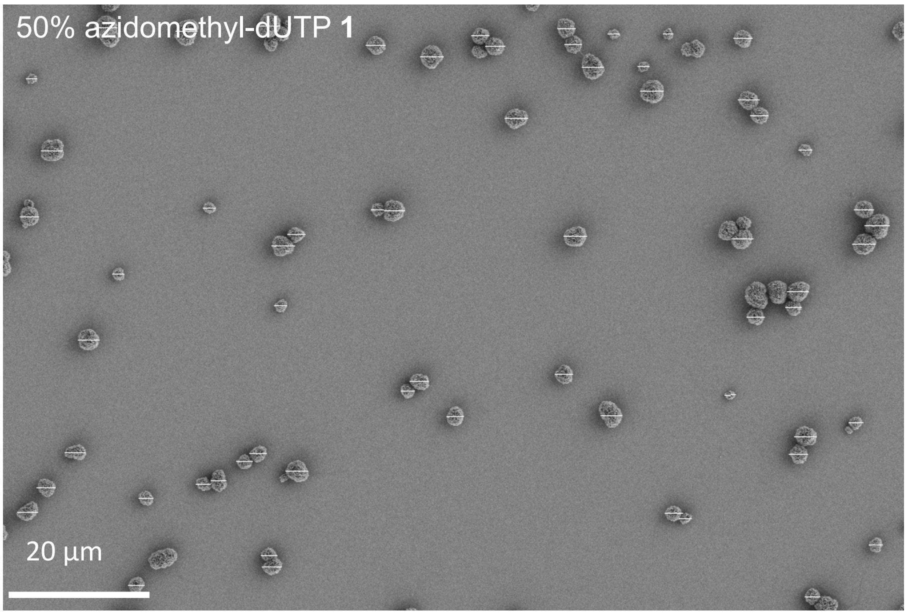

Figure S17. Example SEM image of precipitate formed during phi29 catalysed RCA using 50% azidomethyl-dUTP **1**. Average size of particles measured using ImageJ: 2.45 ± 0.54 µm, n = 64. White lines indicate the individual measurements taken from this image for analysis.

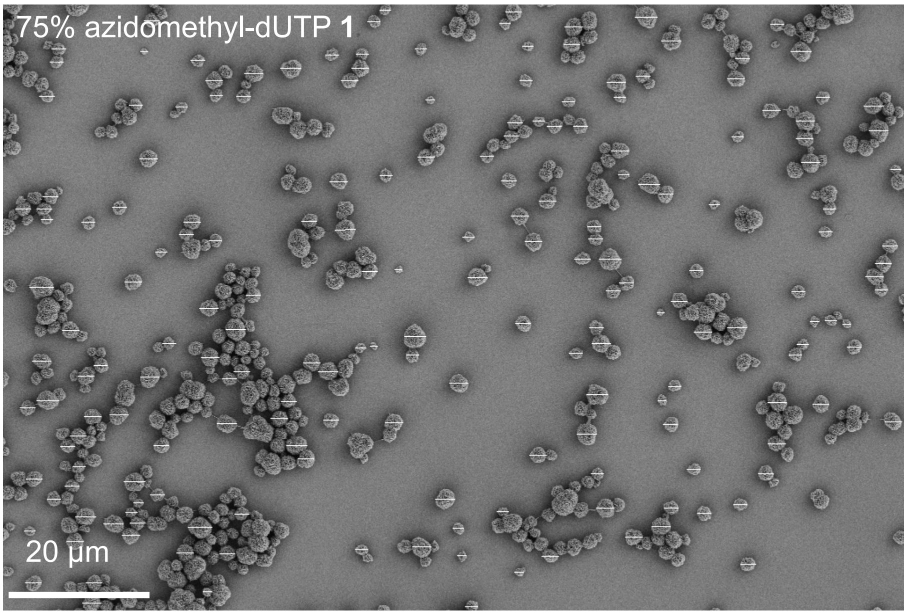

Figure S18. Example SEM image of precipitate formed during phi29 catalysed RCA using 75% azidomethyl-dUTP **1**. Average size of particles measured using ImageJ: 2.08 ± 0.47 µm, n = 196. White lines indicate the individual measurements taken from this image for analysis.

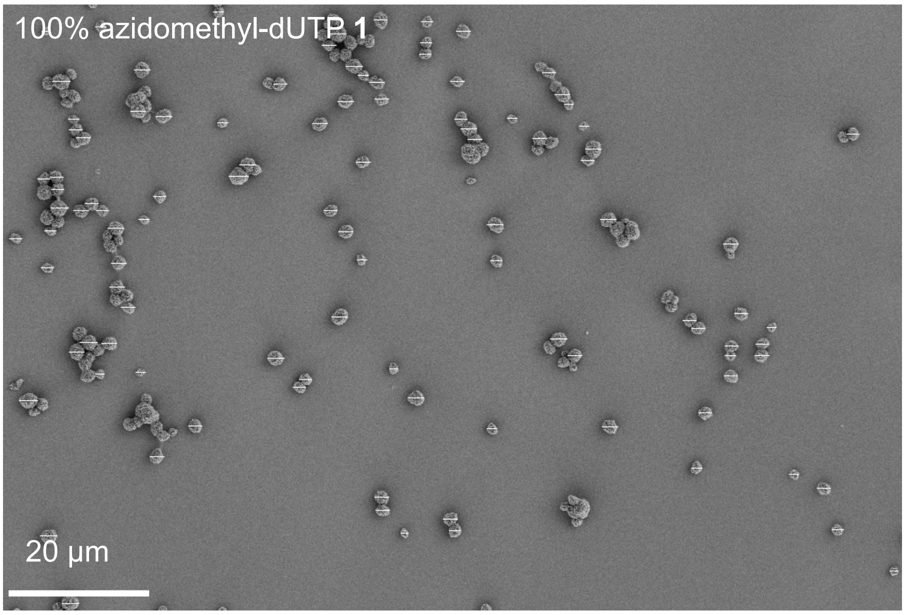

Figure S19. Example SEM image of precipitate formed during phi29 catalysed RCA using 100% azidomethyl-dUTP **1**. Average size of particles measured using ImageJ: 1.83 ± 0.35 μm, n = 106. White lines indicate the individual measurements taken from this image for analysis.

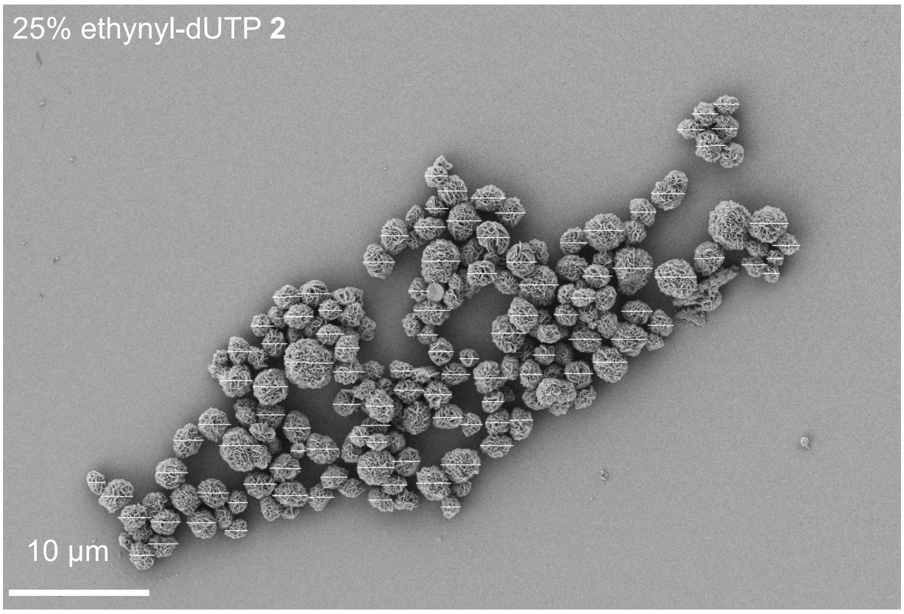

Figure S20. Example SEM image of precipitate formed during phi29 catalysed RCA using 25% ethynyl-dUTP **2**. Average size of particles measured using ImageJ: 2.02 ± 0.42 μm, n = 109. White lines indicate the individual measurements taken from this image for analysis.

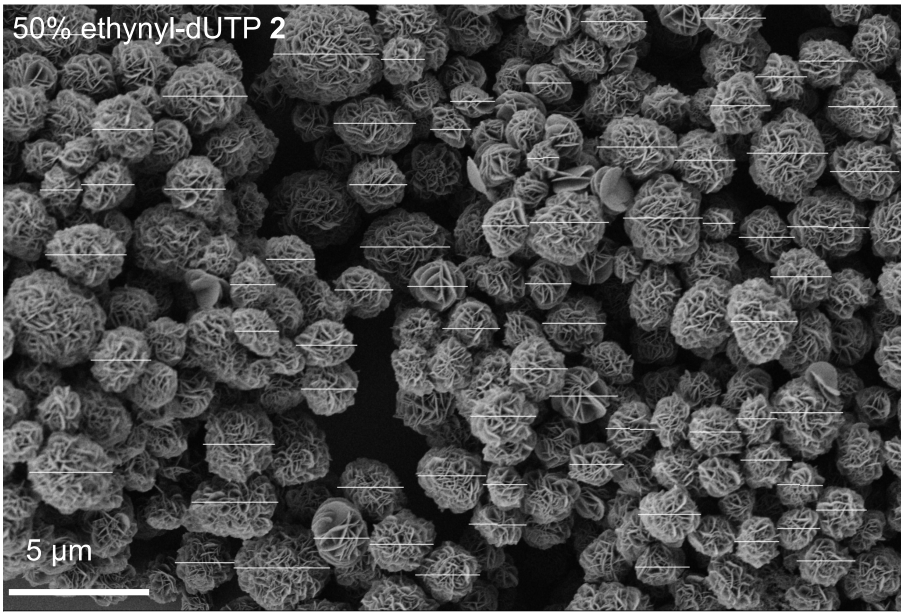

Figure S21. Example SEM image of precipitate formed during phi29 catalysed RCA using 50% ethynyl-dUTP **2**. Average size of particles measured using ImageJ: 2.12 ± 0.53 µm, n = 74. White lines indicate the individual measurements taken from this image for analysis.

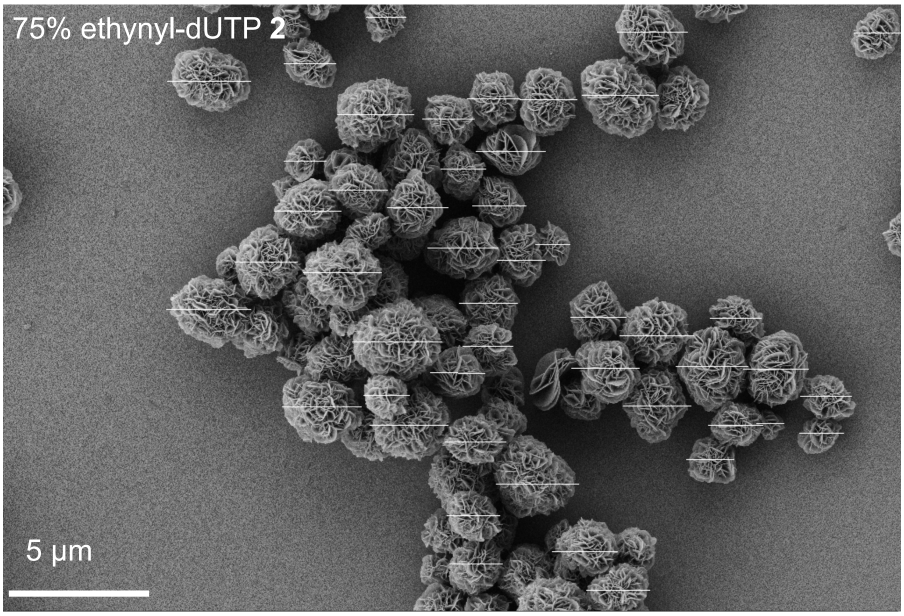

Figure S22. Example SEM image of precipitate formed during phi29 catalysed RCA using 75% ethynyl-dUTP **2**. Average size of particles measured using ImageJ: 2.02 ± 0.47 µm, n = 48. White lines indicate the individual measurements taken from this image for analysis.

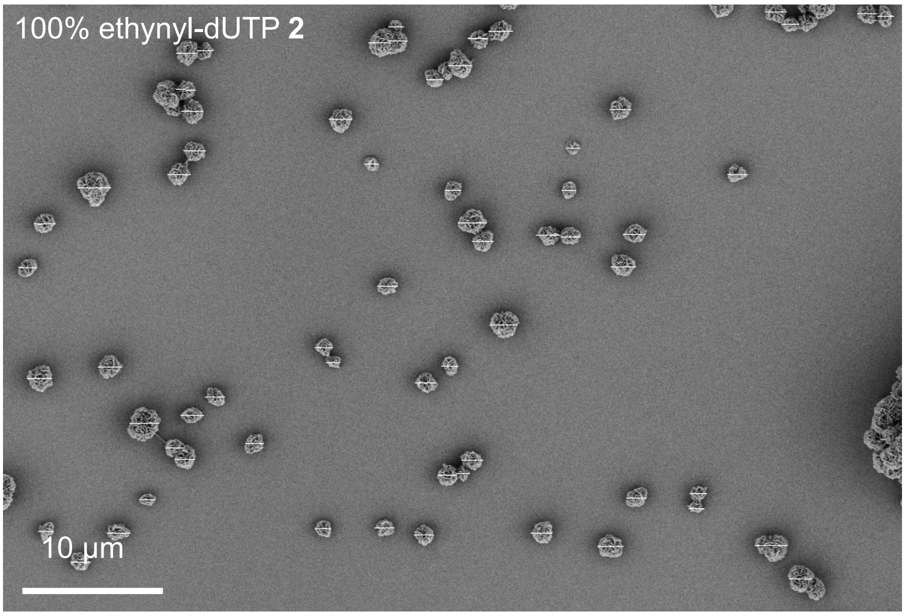

Figure S23. Example SEM image of precipitate formed during phi29 catalysed RCA using 100% ethynyl-dUTP **2**. Average size of particles measured using ImageJ: 1.4 ± 0.30 µm, n = 64. White lines indicate the individual measurements taken from this image for analysis.

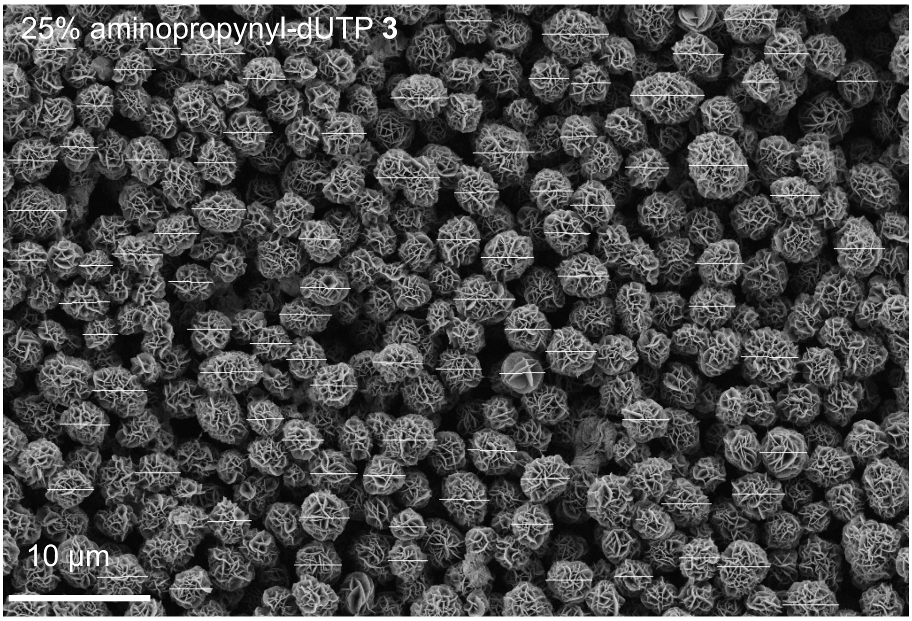

Figure S24. Example SEM image of precipitate formed during phi29 catalysed RCA using 25% aminopropynyl-dUTP **3**. Average size of particles measured using ImageJ: 1.64 ± 0.27 µm, n = 95. White lines indicate the individual measurements taken from this image for analysis.

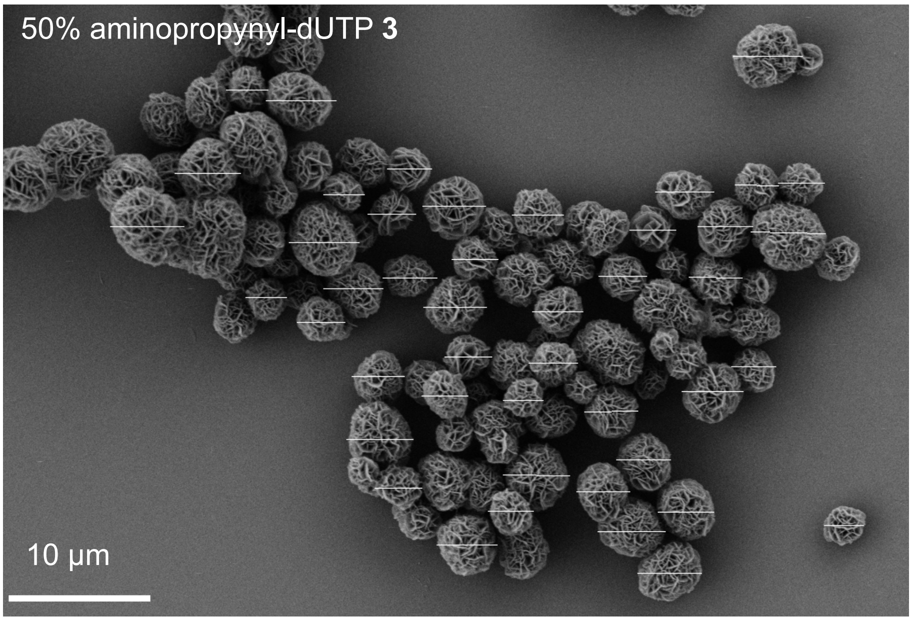

Figure S25. Example SEM image of precipitate formed during phi29 catalysed RCA using 50% aminopropynyl-dUTP **3**. Average size of particles measured using ImageJ: 1.86 ± 0.33 µm, n = 52. White lines indicate the individual measurements taken from this image for analysis.

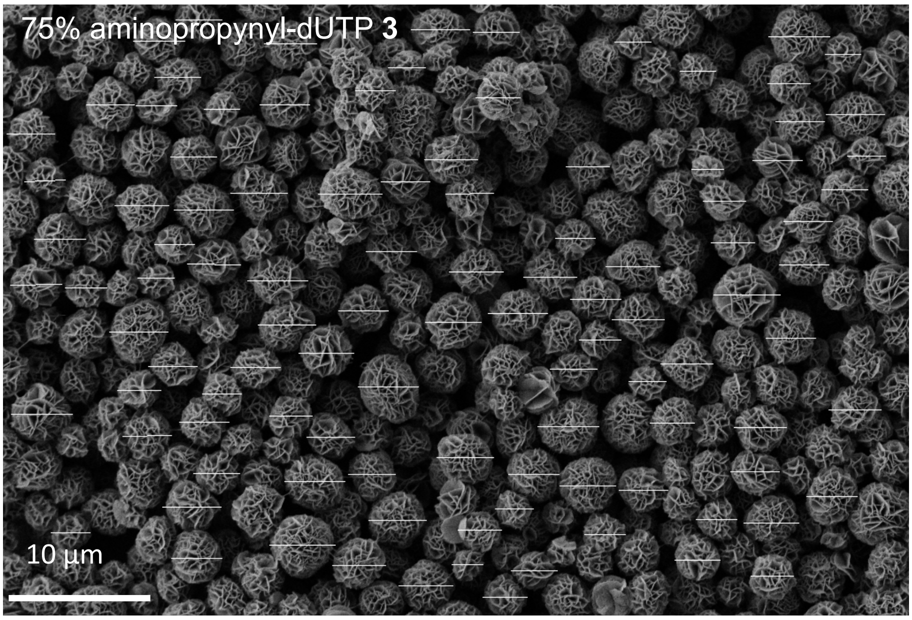

Figure S26. Example SEM image of precipitate formed during phi29 catalysed RCA using 75% aminopropynyl-dUTP **3**. Average size of particles measured using ImageJ: 1.69 ± 0.27 µm, n = 112. White lines indicate the individual measurements taken from this image for analysis.

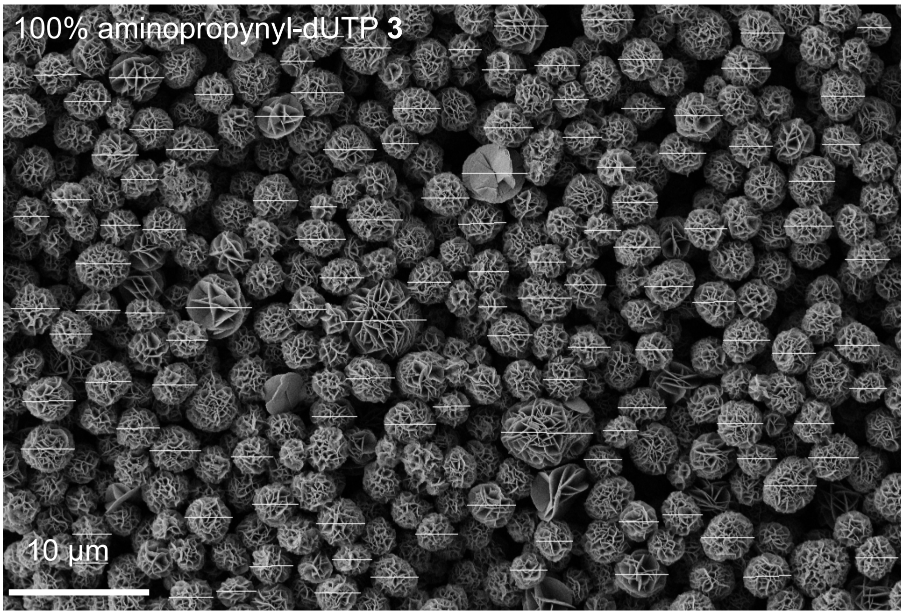

Figure S27. Example SEM image of precipitate formed during phi29 catalysed RCA using 100% aminopropynyl-dUTP **3**. Average size of particles measured using ImageJ: 1.51 ± 0.28 µm, n = 128. White lines indicate the individual measurements taken from this image for analysis.

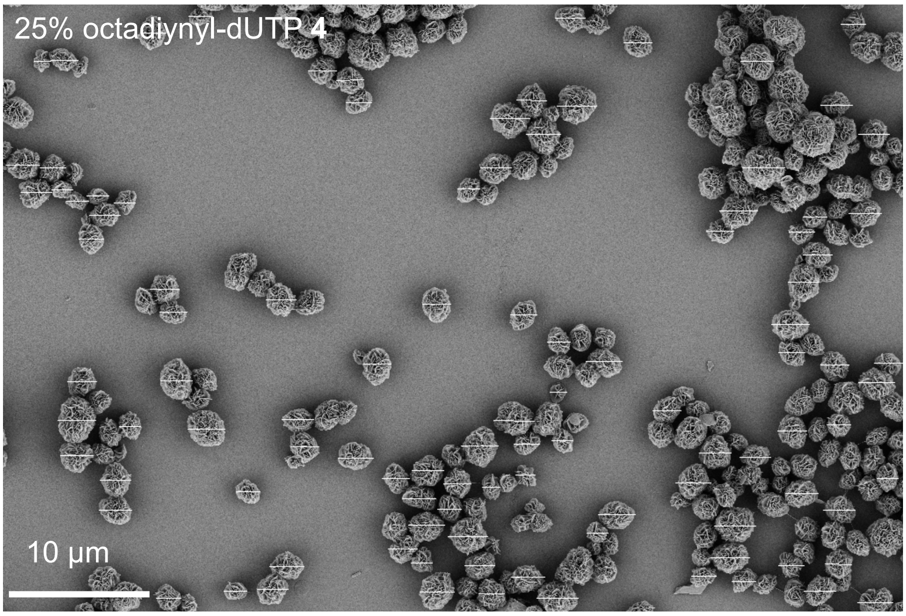

Figure S28. Example SEM image of precipitate formed during phi29 catalysed RCA using 25% octadiynyl-dUTP **4**. Average size of particles measured using ImageJ: 2.07 ± 0.41 µm, n = 108. White lines indicate the individual measurements taken from this image for analysis.

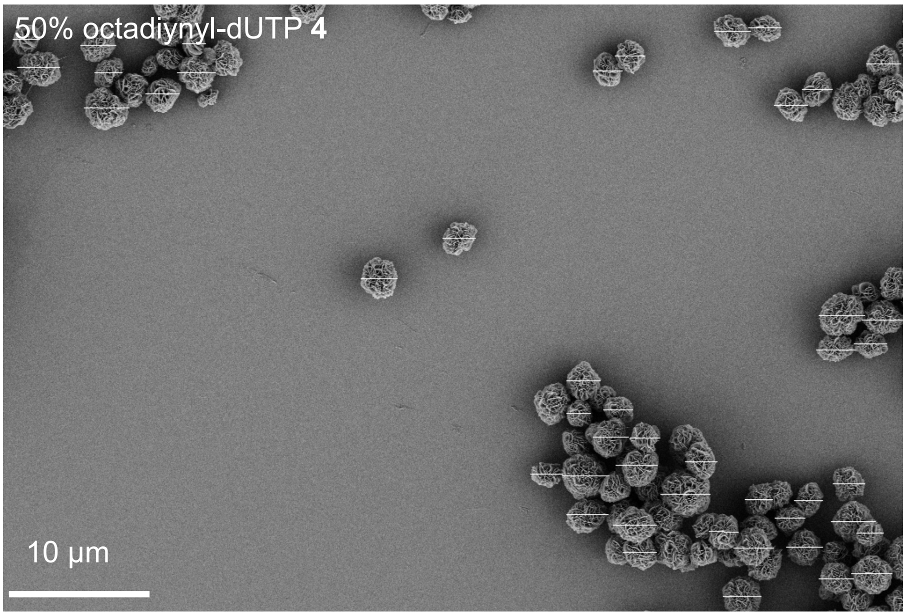

Figure S29. Example SEM image of precipitate formed during phi29 catalysed RCA using 50% octadiynyl-dUTP **4**. Average size of particles measured using ImageJ: 2.45 ± 0.44 µm, n = 46. White lines indicate the individual measurements taken from this image for analysis.

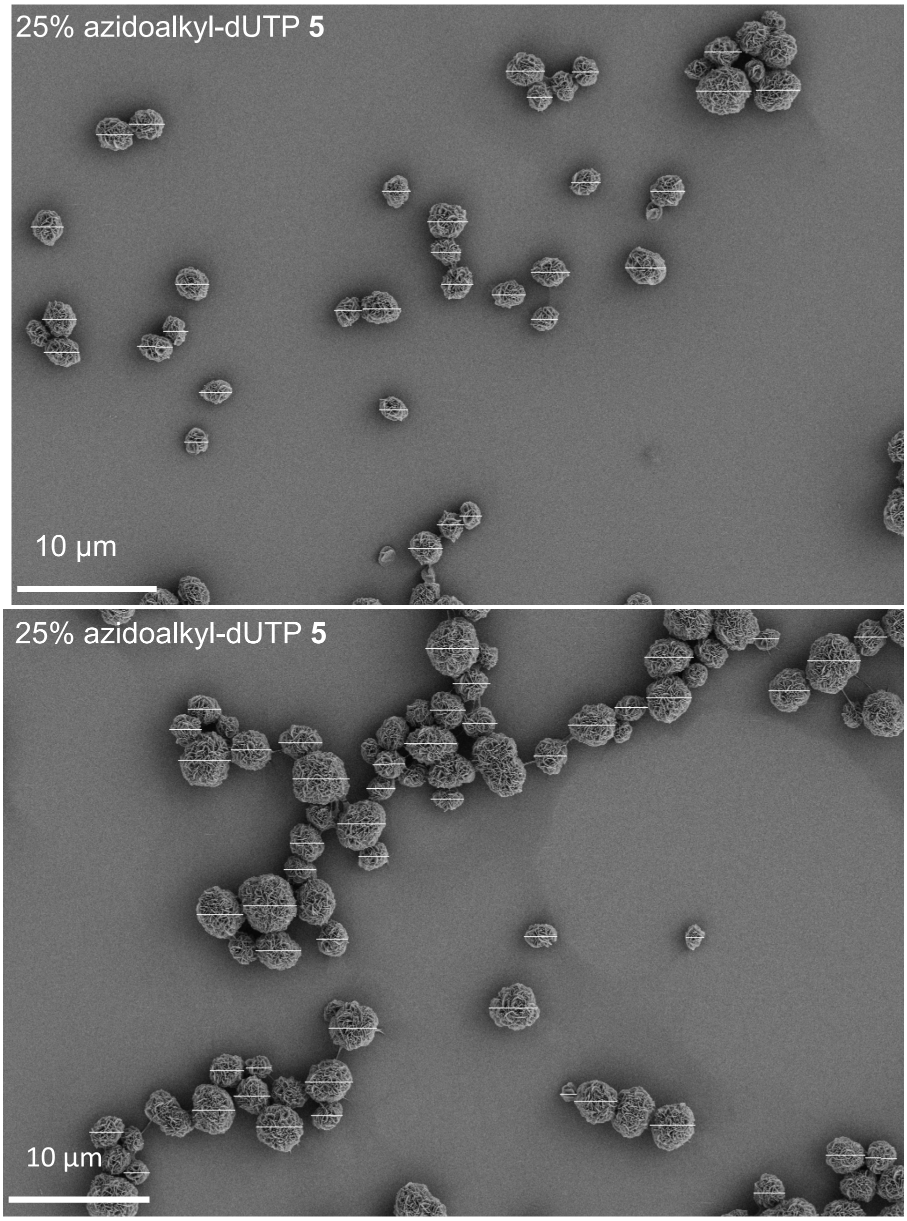

Figure S30. Example SEM images of precipitate formed during phi29 catalysed RCA using 25% azidoalkyl-dUTP **5**. Average size of particles measured using ImageJ: 2.57 ± 0.64 µm, n = 83. White lines indicate the individual measurements taken from this image for analysis. Two images were used to increase the number of particles counted.

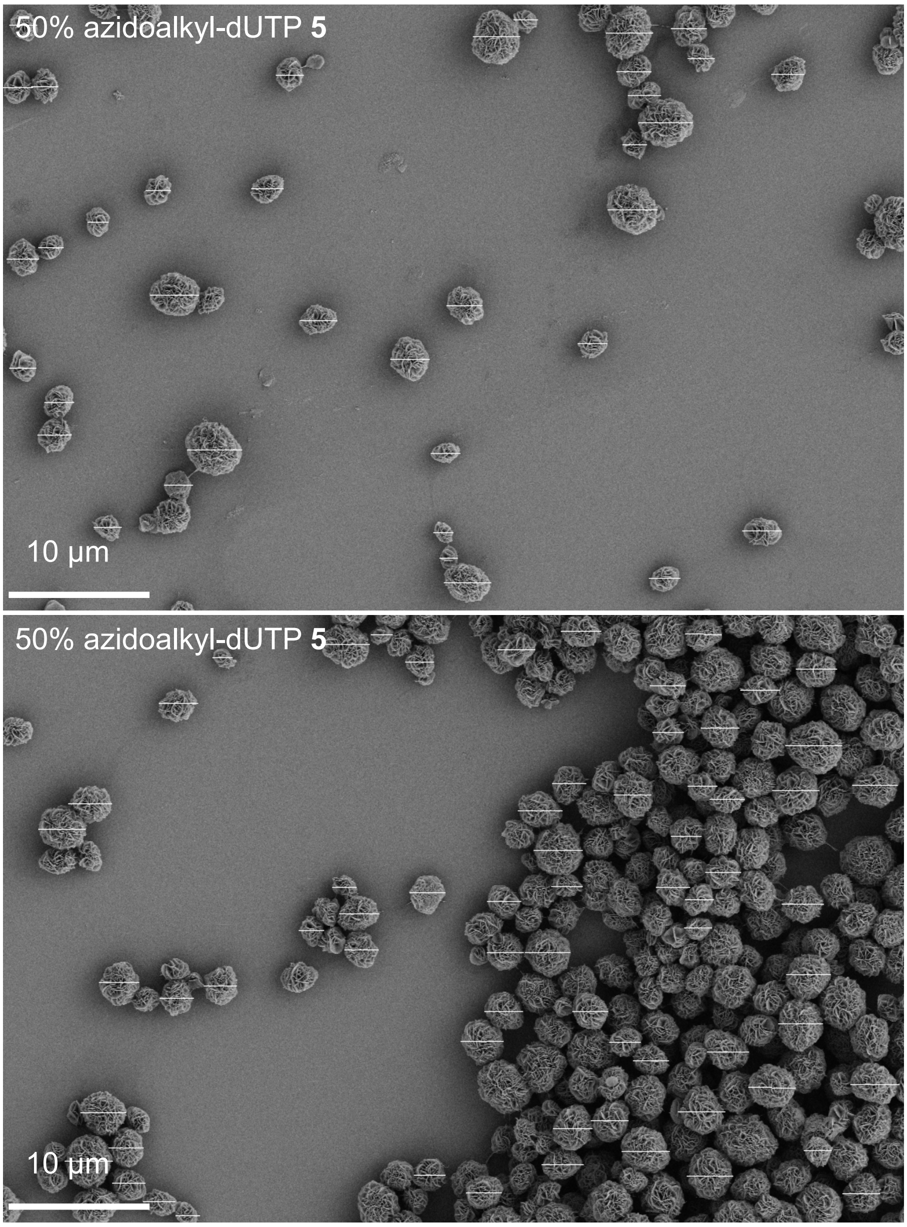

Figure S31. Example SEM image of precipitate formed during phi29 catalysed RCA using 50% azidoalkyl-dUTP **5**. Average size of particles measured using ImageJ: 2.51 ± 0.59 µm, n = 107. White lines indicate the individual measurements taken from this image for analysis. Two images were used to increase the number of particles counted.

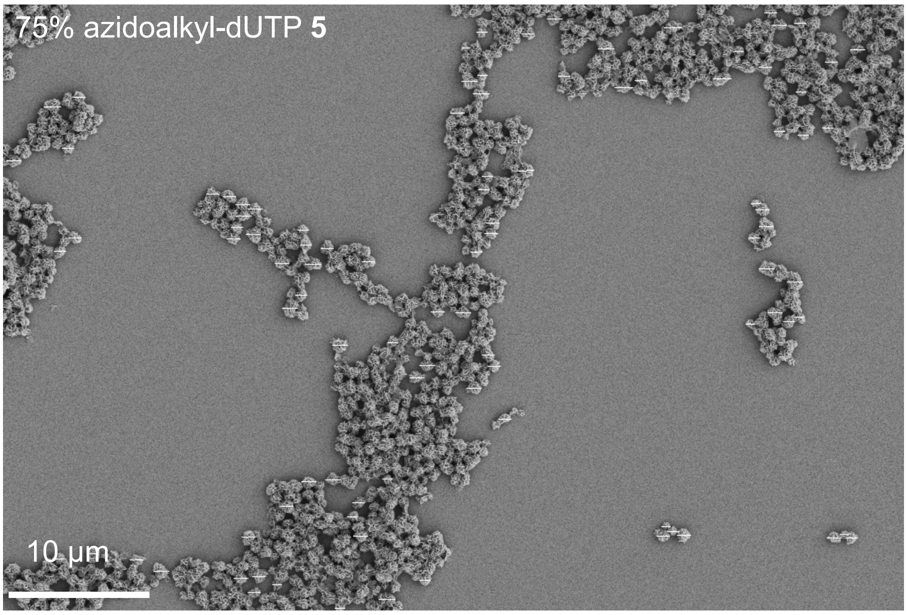

Figure S32. Example SEM image of precipitate formed during phi29 catalysed RCA using 75% azidoalkyl-dUTP **5**. Average size of particles measured using ImageJ: 0.42 ± 0.07 µm, n = 99. White lines indicate the individual measurements taken from this image for analysis.

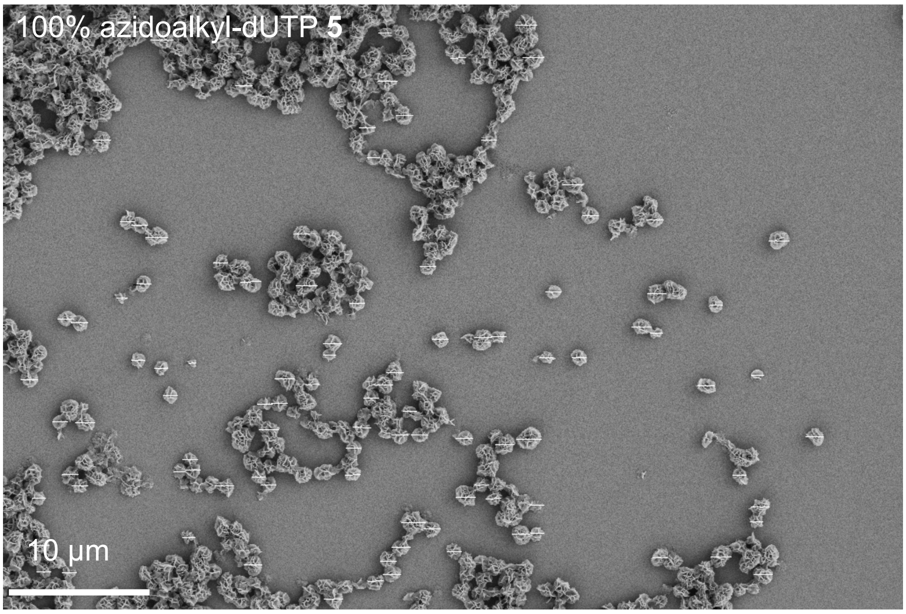

Figure S33. Example SEM image of precipitate formed during phi29 catalysed RCA using 100% azidoalkyl-dUTP **5**. Average size of particles measured using ImageJ: 0.53 ± 0.10 µm, n = 105. White lines indicate the individual measurements taken from this image for analysis.

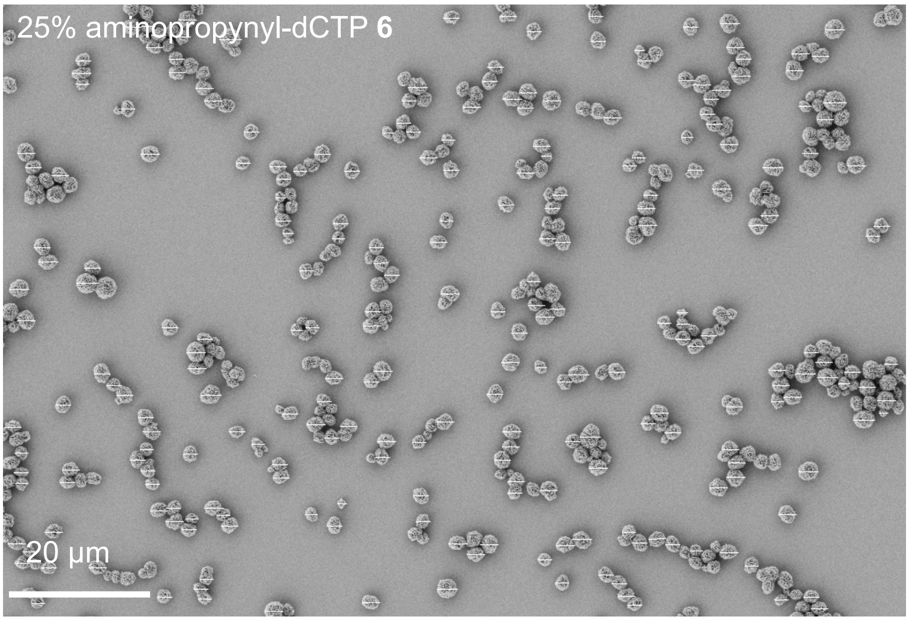

Figure S34. Example SEM image of precipitate formed during phi29 catalysed RCA using 25% aminopropynyl-dCTP **6**. Average size of particles measured using ImageJ: 2.18 ± 0.53 µm, n = 252. White lines indicate the individual measurements taken from this image for analysis.

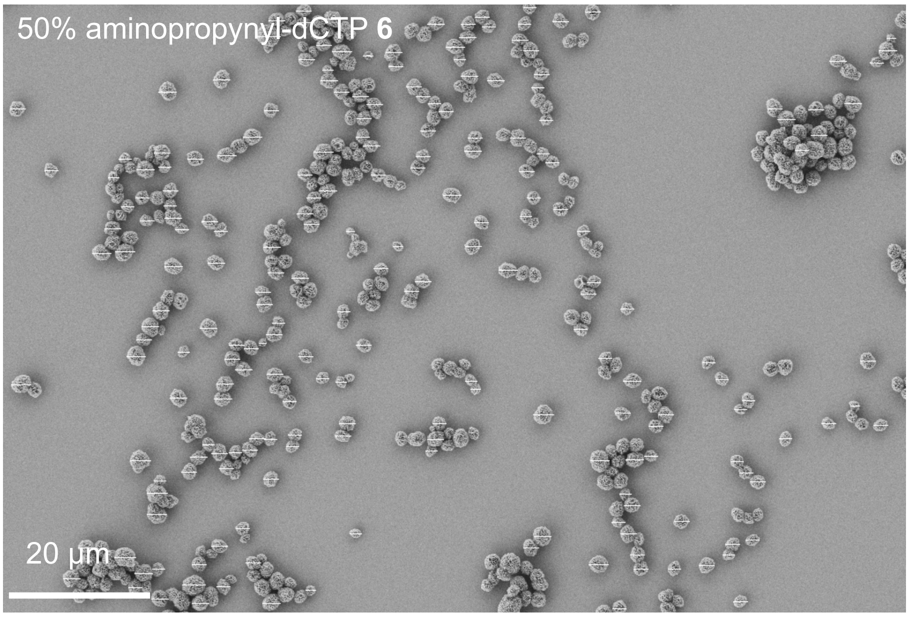

Figure S35. Example SEM image of precipitate formed during phi29 catalysed RCA using 50% aminopropynyl-dCTP **6**. Average size of particles measured using ImageJ: 2.02 ± 0.34 µm, n = 219. White lines indicate the individual measurements taken from this image for analysis.

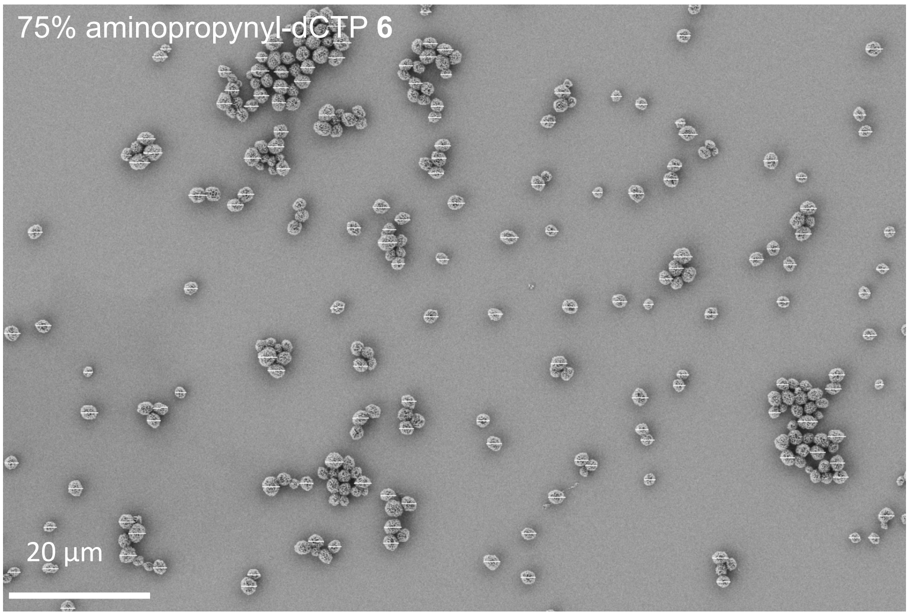

Figure S36. Example SEM image of precipitate formed during phi29 catalysed RCA using 75% aminopropynyl-dCTP **6**. Average size of particles measured using ImageJ: 1.98 ± 0.31 µm, n = 156. White lines indicate the individual measurements taken from this image for analysis.

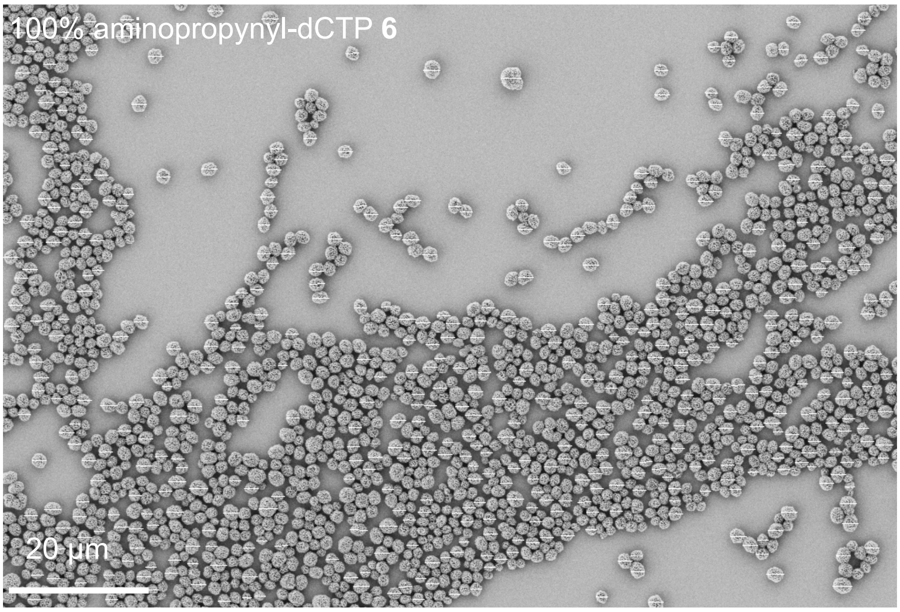

Figure S37. Example SEM image of precipitate formed during phi29 catalysed RCA using 100% aminopropynyl-dCTP **6**. Average size of particles measured using ImageJ: 1.84 ± 0.26 µm, n = 510. White lines indicate the individual measurements taken from this image for analysis.

4.4. Choice of buffer experiments

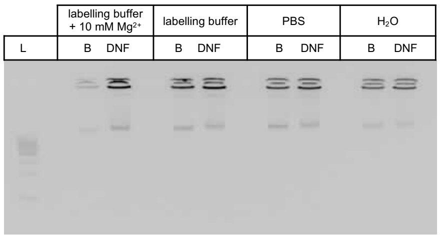

Figure S38. The choice of buffer can lead to DNA leaching from DNFs. DNFs were incubated in the different buffers overnight indicated above the gel. The samples were centrifuged to separate the DNF from the buffer. The DNA present in the DNFs and in the buffer (B) was analysed by 0.8% agarose gel electrophoresis. The presence of DNA in the buffer indicates that the DNA falls off the surface of the DNFs. L) 1 kb DNA ladder; B) buffer. Labelling buffer = 50% DMSO, 10 mM HEPES, pH 8.0, 10 mM $MgSO_4$.

4.5. Labelling analysis

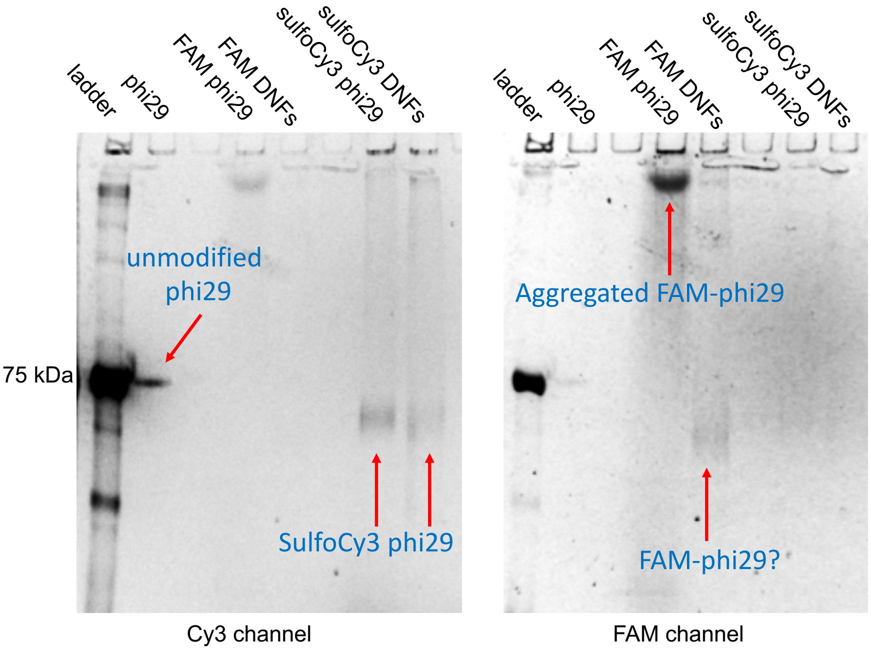

Figure S39. 8% SDS-PAGE analysis of the protein content of unmodified DNFs after NHS labelling with either FAM-NHS or sulfo-Cy3-NHS using the conditions described in section 1.8. DNFs were dissolved in TBE buffer before SDS-PAGE and the samples compared to the phi29 labelled as described in section 1.12. FAM appeared to cause phi29 to aggregate; however, the sulfo-Cy3 experiment suggests that the non-specific labelling was occurring on phi29. We hypothesise that the phi29 present in DNFs did not aggregate as some of the amines would be protected by interaction with the DNF reducing the dye:protein ratio.

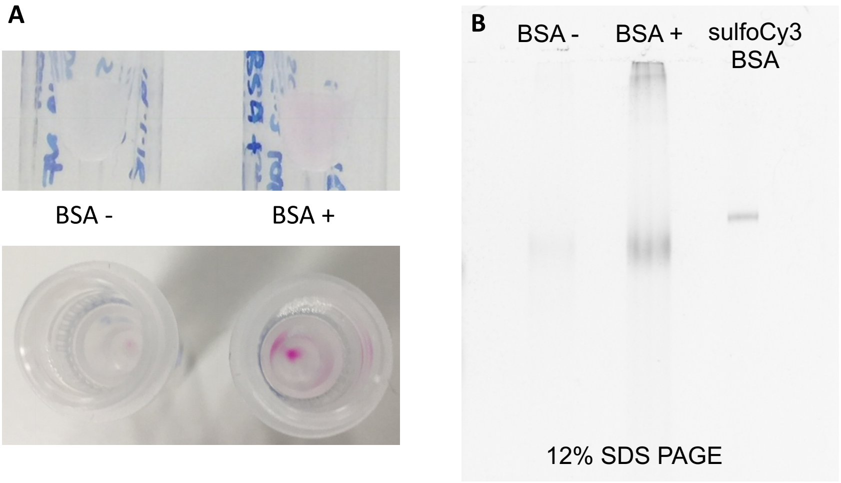

Figure S40. Using encapsulated protein as a labelling platform. (A) Appearance of DNFs with (BSA +) or without (BSA -) present in the amplification buffer after labelling with sulfo-Cy3-NHS; (B) 12% SDS-PAGE of the same samples. We chose BSA for this proof-of-principle study because it has 59 lysine residues, of which 30-35 have primary amines that would be available for functionalisation, and also because BSA is encapsulated into DNFs. (5) After RCA, the DNFs were collected and labelled as described in the main text. Sulfo-Cy3-NHS was used as it was found to separate best by SDS-PAGE with the phi29 experiments (Figure S39). As a control BSA was also labelled with sulfoCy3-NHS. The BSA-DNFs showed a visibly strong pink colour when compared with DNFs prepared in the absence of BSA but treated using the same conditions (A). The DNFs were then analysed by SDS-PAGE (B). A streaky band was observed, which we attributed to the protein interacting with the polyanionic DNA during electrophoresis.

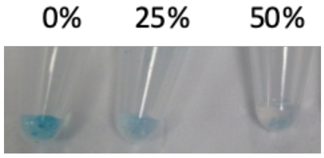

Figure S41. Non-specific labelling of N_3-DNFs. DNFs prepared with varying ratios of azidoalkyl-dUTP **5** (as indicated above the samples) and incubated with Cy5-BCN. Less material is observed in the 50% sample, as this triphosphate significantly reduces the RCA yield (discussed in paper). 0% modified appears more intense as far more precipitate is produced.

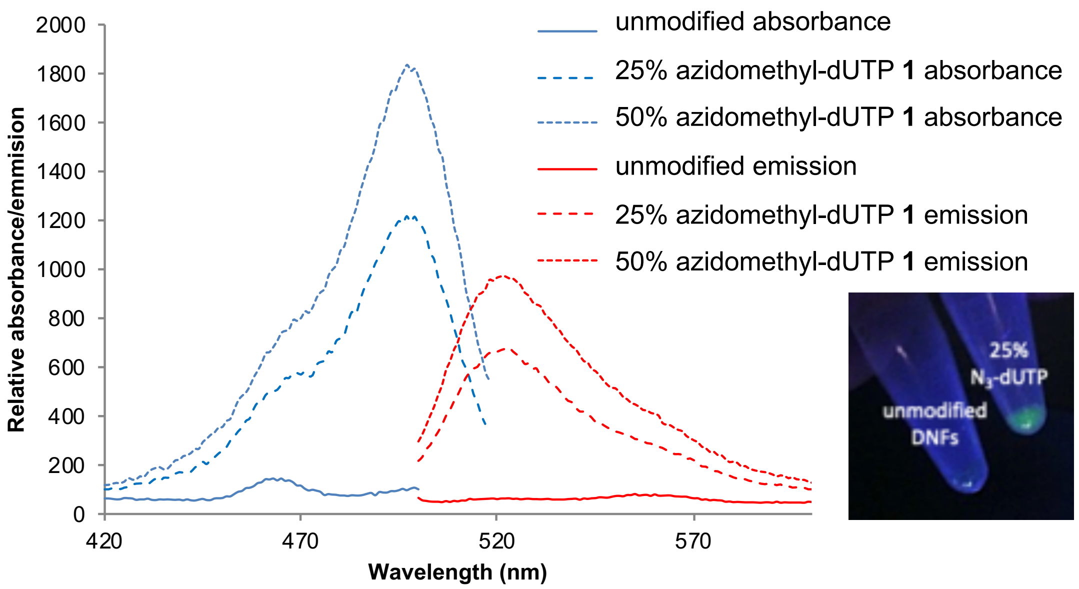

Figure S42. Absorbance (blue) and emission (red) spectra of N_3-DNFs after treatment with BCN-FAM measured using a ClarioStar plate reader. Inset) Unmodified DNFs and N_3-DNFs prepared using 25% azidomethyl-dUTP **1** treated with FAM-BCN and visualised using a TLC lamp (N_3-dUTP = azidomethyl-dUTP **1**). These results confirm that non-specific labelling was not occurring.

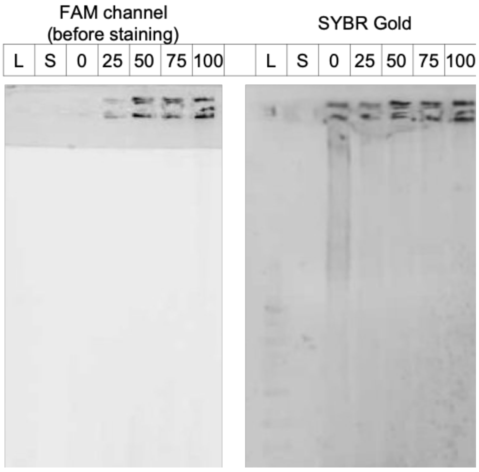

Figure S43. Agarose gel electrophoresis of FAM-BCN labelled N_3-DNFs confirming that the dye is attached to the DNA. Different percentages of azidomethyl-dUTP **1** were used to prepare the DNFs as indicated above the lanes. In all cases the total dNTP concentration was kept at 2 mM. L) 1 kb DNA ladder; S) splint 1. The gel was first imaged using a SYNGENE G-box using the EpiBlue (460 nm) excitation and short wavelength (516-600 nm) emission settings, stained with SYBR Gold, and then reimaged using the Transilluminator excitation setting and the EtBr/UV (572-626 nm) emission setting.

4.6. Dual labelling experiments

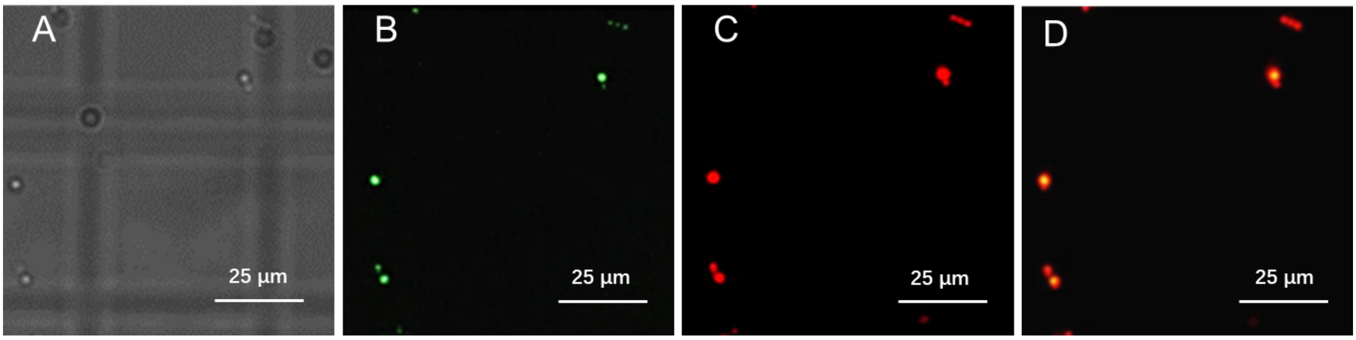

Figure S44. Dual labelled DNFs. DNFs were prepared using 25% azidomethyl-dUTP **1** and 25% aminopropynyl-dCTP **6** and simultaneously labelled with FAM-BCN and TAMRA-NHS. A) Brightfield image of dual labelled DNFs; B) Fluorescence image under 475 nm excitation confirming attachment of FAM; C) Fluorescence image under 542 nm excitation confirming attachment of TAMRA; D) Overlay of B and C.

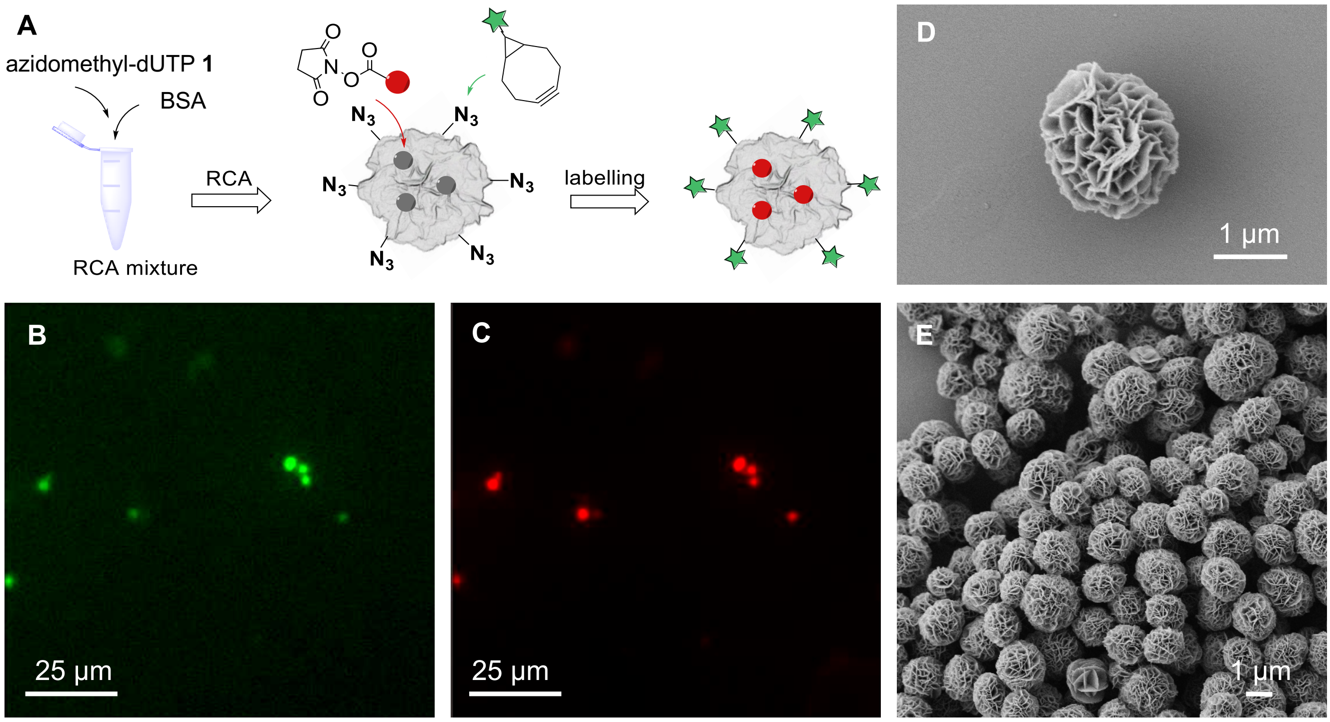

Figure S45. A) Dual labelling approach utilising the encapsulated protein (BSA) as an amine source and 50% azidomethyl-dUTP **1** as the azide source; B) Fluorescence image under 475 nm excitation confirming attachment of FAM; C) Fluorescence image under 542 nm excitation confirming attachment of TAMRA; D and E) SEM image of dual labelled construct. These results confirm that it is possible to functionalise BSA with TAMRA-NHS and the DNA modified with azidomethyl-dUTP **1** with FAM-BCN.

4.7. Peptide and aptamer synthesis

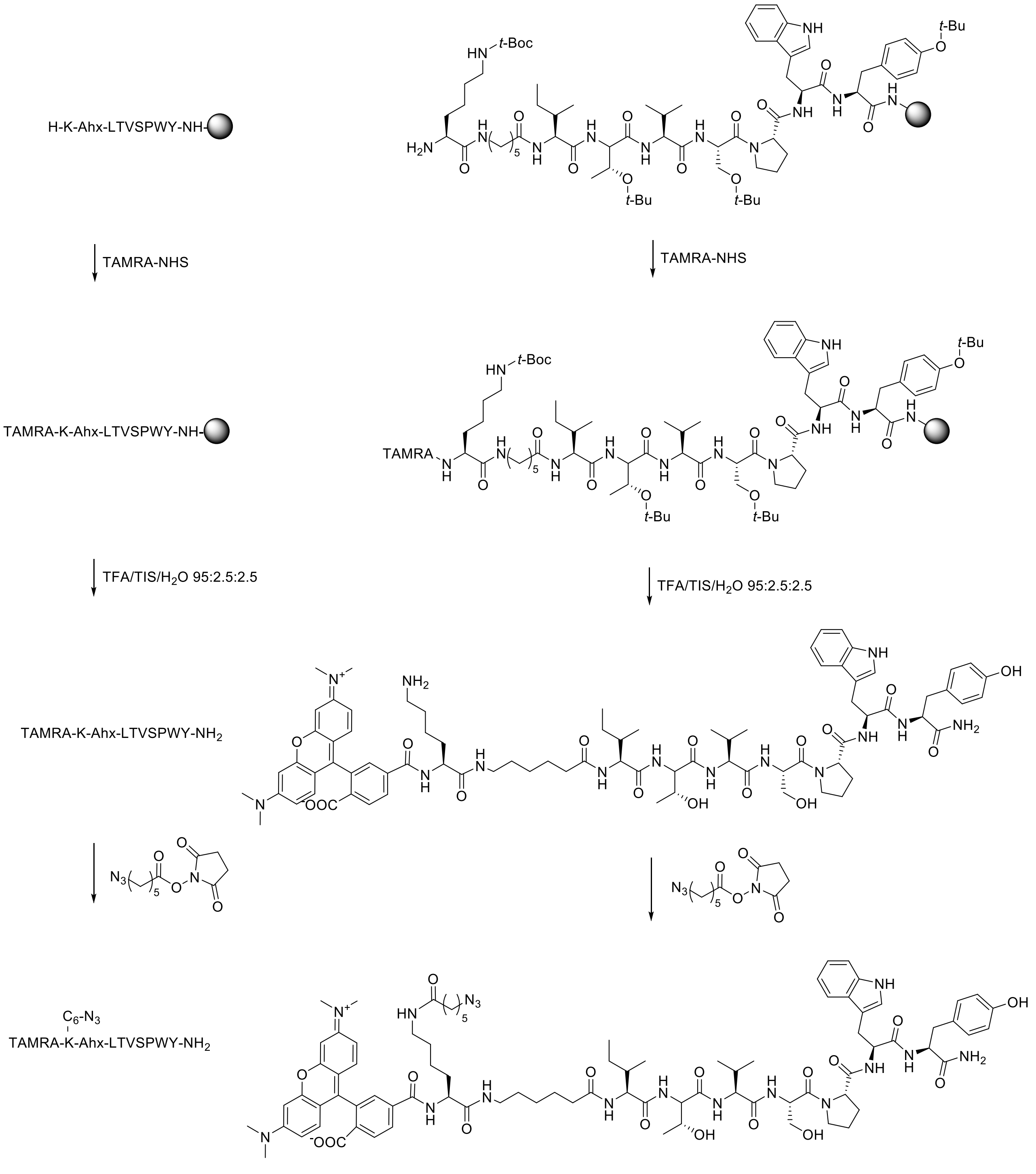

Figure S46. Synthesis of TAMRA labelled azide-functionalised peptide **1** used in this study.

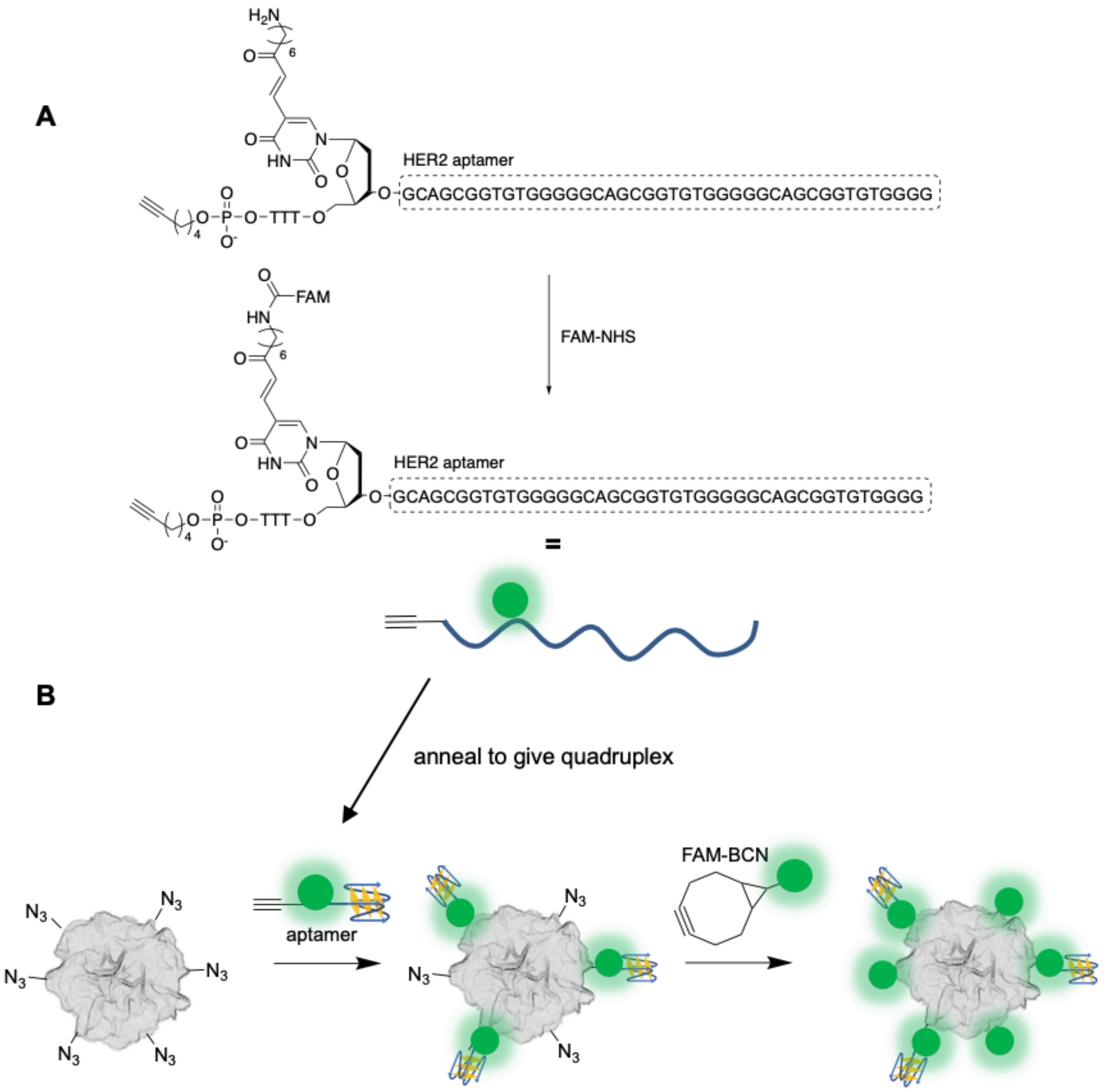

Figure S47. A) Synthesis of HER2 aptamer and B) subsequent fApt-DNF construction. FAM-BCN was added in a second step as it was found to increase the fluorescence of the particles. This could be because some azides were inaccessible to the relatively large alkyne functionalised aptamer.

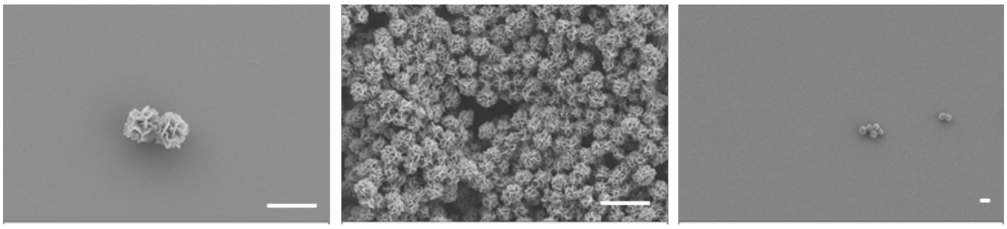

Figure S48. SEM images of fAPt-DNFs produced with 25% azidomethyl-dUTP **1**, 25 mM Mg^{2+}, and labelled with HER2 aptamer. Scale bars represent 1 µm. 25 mM Mg^{2+} was used to reduce the particle size for the FACS experiments.

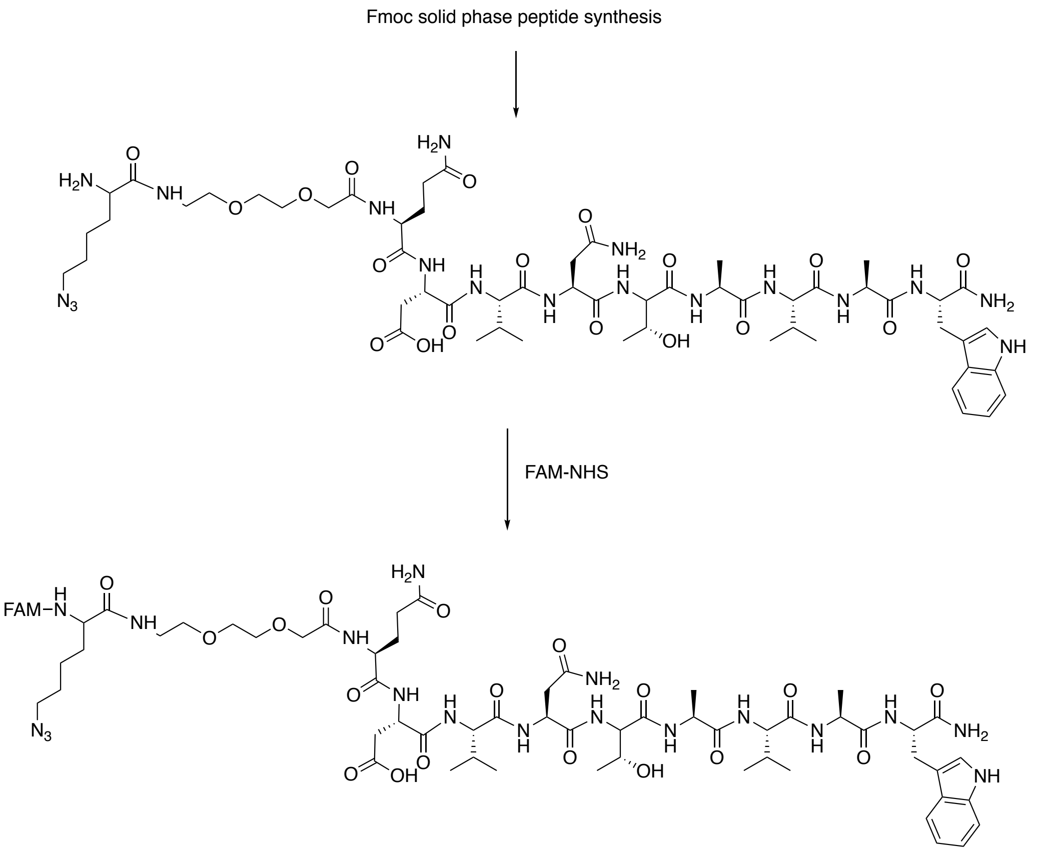

Figure S49. Synthesis of FAM labelled HER2 peptide **2** for the preparation of pept-DNFs.

5. Mass spectra of DNA templates, DNA splints and peptides used in this study

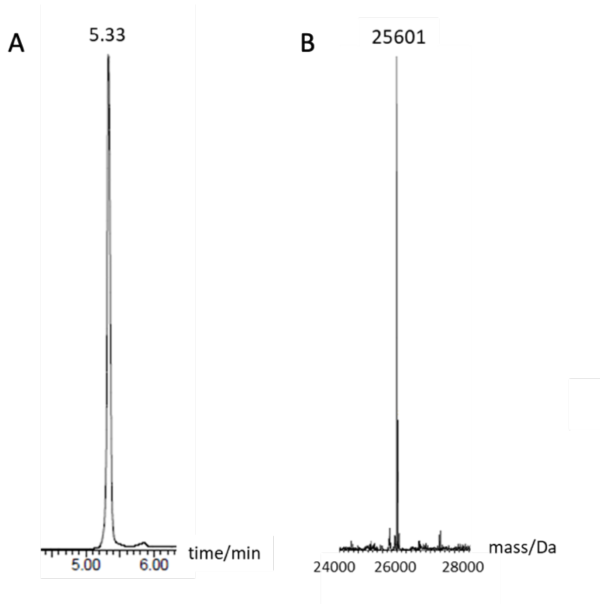

Figure S50. (A) RP-UPLC (UV abs at 260 nm) and (B) mass spectrum (ES^-) of template 1, calculated 25597 Da, found 25601 Da.

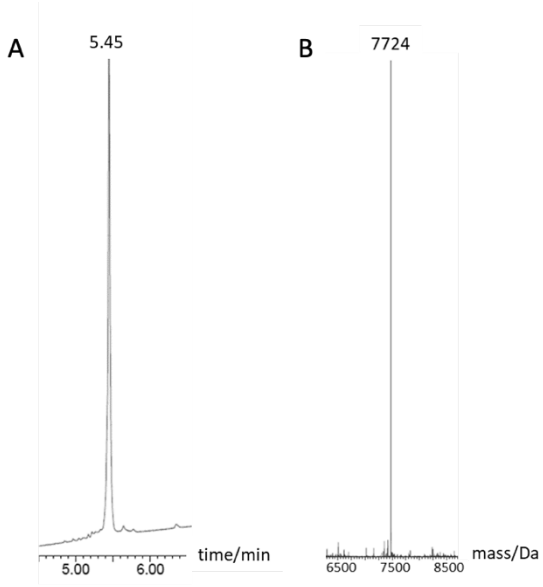

Figure S51. (A) RP-UPLC (UV abs at 260 nm) and (B) mass spectrum (ES^-) of splint 1, calculated 7724 Da, found 7724 Da.

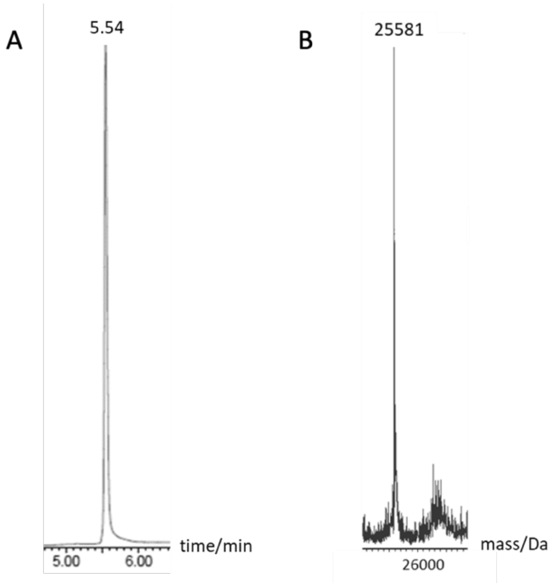

Figure S52. (A) RP-UPLC (UV abs at 260 nm) and (B) mass spectrum (ES$^-$) of cyclic template 1, calculated 25579 Da, found 25581 Da.

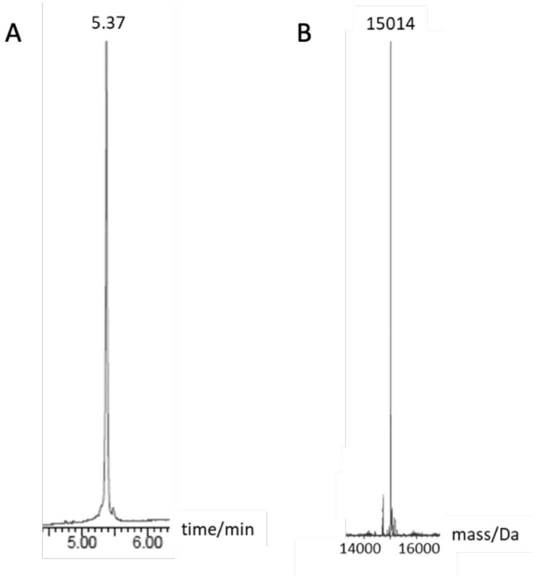

Figure S53. (A) RP-UPLC (UV abs at 260 nm) and (B) mass spectrum (ES$^-$) of template 2, calculated 15012 Da, found 15014 Da.

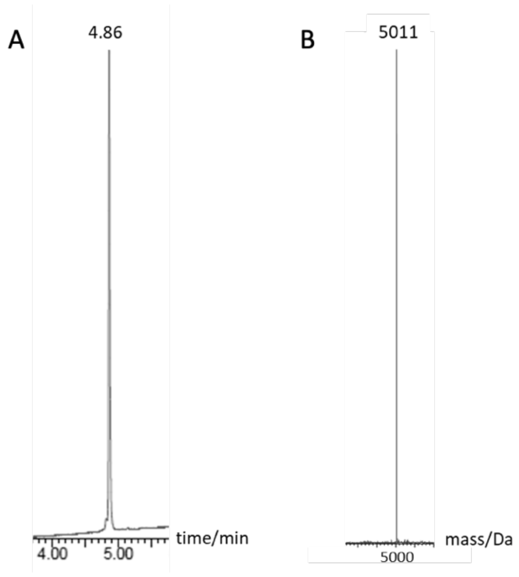

Figure S54. (A) RP-UPLC (UV abs at 260 nm) and (B) mass spectrum (ES$^-$) of splint 2, calculated 5010 Da, found 5011 Da.

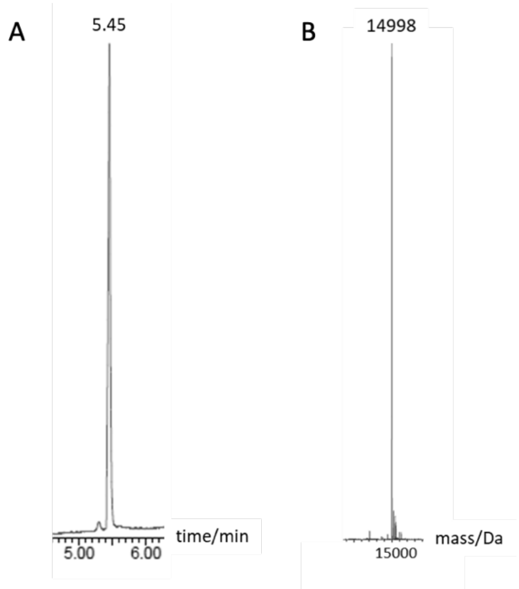

Figure S55. (A) RP-UPLC (UV abs at 260 nm) and (B) mass spectrum (ES-) of cyclic template 2, calculated 14994 Da, found 14998 Da.

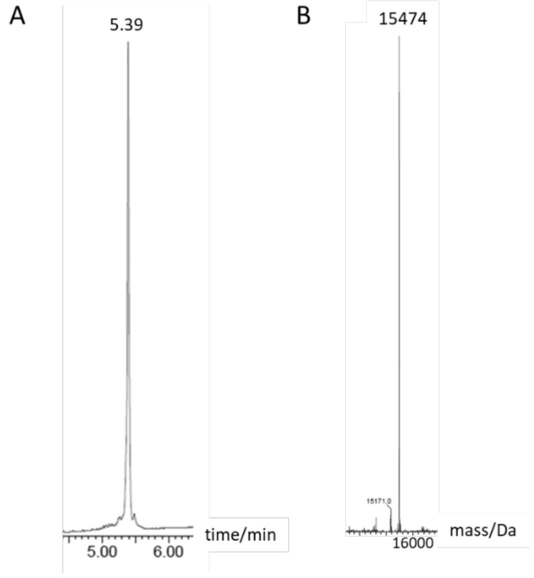

Figure S56. (A) RP-UPLC (UV abs at 260 nm) and (B) mass spectrum (ES-) of template 3, calculated 15473 Da, found 15474 Da.

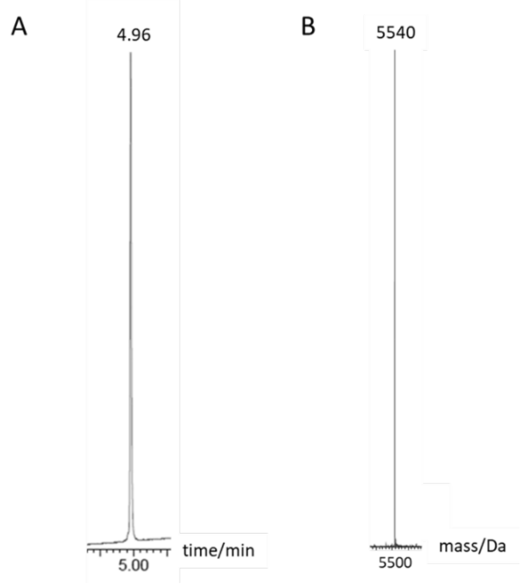

Figure S57. (A) RP-UPLC (UV abs at 260 nm) and (B) mass spectrum (ES-) of splint 3, calculated 5539 Da, found 5540 Da.

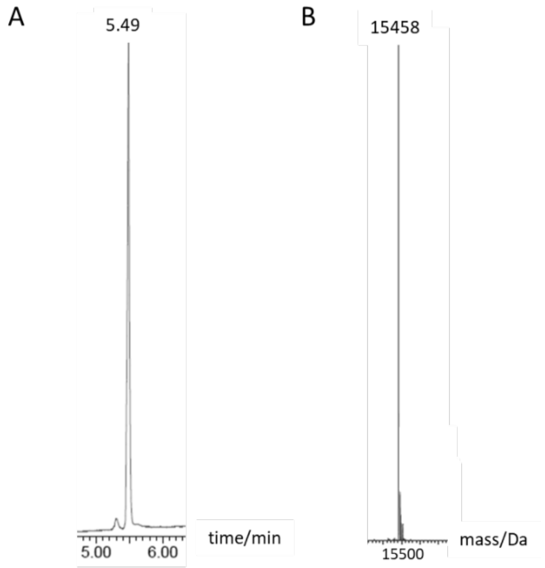

Figure S58. (A) RP-UPLC (UV abs at 260 nm) and (B) mass spectrum (ES-) of cyclic template 3, calculated 15456 Da, found 15458 Da.

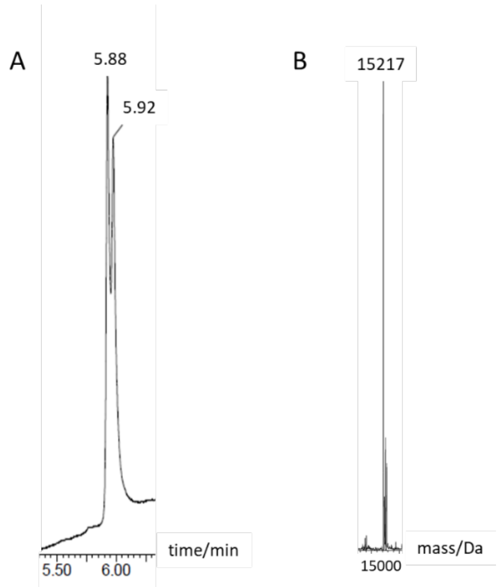

Figure S59. (A) RP-UPLC (UV abs at 260 nm) and (B) mass spectrum (ES-) of 5′-alkyne, FAM modified HER2 template, calculated 15216 Da, found 15217 Da. Two peaks are observed in the RP-UPLC trace due to a mixture of the 5 and 6 isomers of FAM.

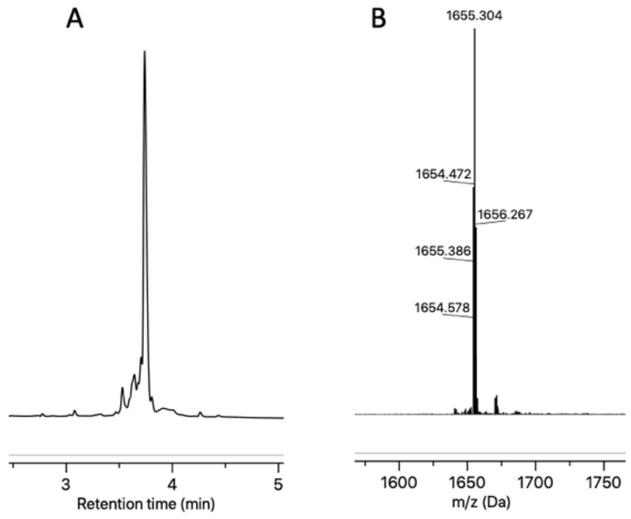

Figure S60. (A) RP-UPLC (UV abs at 254 nm) and (B) mass spectrum (ES^+) TAMRA labelled peptide 1, calculated 1655.9 Da, found 1655.3 Da.

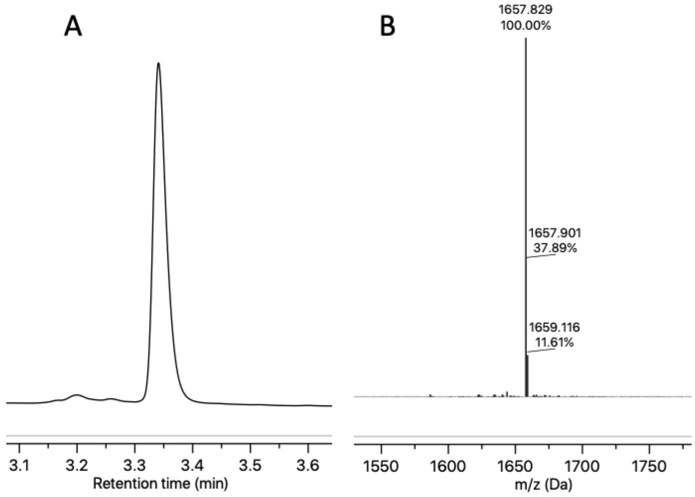

Figure S61. (A) RP-UPLC (UV abs at 254 nm) and (B) mass spectrum (ES^+) of HER2 targeting FAM labelled peptide 2 calculated 1658.7 Da, found 1657.8 Da.

6. References

1. Hu, R., Zhang, X., Zhao, Z., Zhu, G., Chen, T., Fu, T. and Tan, W. (2014) DNA nanoflowers for multiplexed cellular imaging and traceable targeted drug delivery. *Angew. Chem. Int. Ed. Engl.*, **53**, 5821-5826.
2. Chen, J., Baker, Y.R., Brown, A., El-Sagheer, A.H. and Brown, T. (2018) Enzyme-free synthesis of cyclic single-stranded DNA constructs containing a single triazole, amide or phosphoramidate backbone linkage and their use as templates for rolling circle amplification and nanoflower formation. *Chem Sci*, **9**, 8110-8120.
3. Shadidi, M. and Sioud, M. (2003) Identification of novel carrier peptides for the specific delivery of therapeutics into cancer cells. *FASEB J.*, **17**, 256-258.
4. Calce, E., Monfregola, L., Sandomenico, A., Saviano, M. and De Luca, S. (2013) Fluorescence study for selecting specific ligands toward HER2 receptor: An example of receptor fragment approach. *European Journal of Medicinal Chemistry*, **61**, 116-121.
5. Kim, E., Zwi-Dantsis, L., Reznikov, N., Hansel, C.S., Agarwal, S. and Stevens, M.M. (2017) One-Pot Synthesis of Multiple Protein-Encapsulated DNA Flowers and Their Application in Intracellular Protein Delivery. *Adv. Mater.*, **29**, 1701086.
